# Evaluation of Substituted Pyrazole-Based Kinase Inhibitors in One Decade (2011–2020): Current Status and Future Prospects

**DOI:** 10.3390/molecules27010330

**Published:** 2022-01-05

**Authors:** Mohammed I. El-Gamal, Seyed-Omar Zaraei, Moustafa M. Madkour, Hanan S. Anbar

**Affiliations:** 1 Department of Medicinal Chemistry, College of Pharmacy, University of Sharjah, Sharjah 27272, United Arab Emirates; 2 Sharjah Institute for Medical Research, University of Sharjah, Sharjah 27272, United Arab Emirates; holor@live.com (S.-O.Z.); moustafatrika22@hotmail.com (M.M.M.); 3 Department of Medicinal Chemistry, Faculty of Pharmacy, University of Mansoura, Mansoura 35516, Egypt; 4 Department of Clinical Pharmacy and Pharmacotherapeutics, Dubai Pharmacy College for Girls, Dubai 19099, United Arab Emirates

**Keywords:** anticancer, anti-inflammatory, kinase inhibitor, neurodegenerative disorders, pyrazole

## Abstract

Pyrazole has been recognized as a pharmacologically important privileged scaffold whose derivatives produce almost all types of pharmacological activities and have attracted much attention in the last decades. Of the various pyrazole derivatives reported as potential therapeutic agents, this article focuses on pyrazole-based kinase inhibitors. Pyrazole-possessing kinase inhibitors play a crucial role in various disease areas, especially in many cancer types such as lymphoma, breast cancer, melanoma, cervical cancer, and others in addition to inflammation and neurodegenerative disorders. In this article, we reviewed the structural and biological characteristics of the pyrazole derivatives recently reported as kinase inhibitors and classified them according to their target kinases in a chronological order. We reviewed the reports including pyrazole derivatives as kinase inhibitors published during the past decade (2011–2020).

## 1. Introduction

Pyrazole derivatives have attracted much attention during the last decades due to their interesting pharmacological properties and manifold applications. They are among the most extensively investigated groups of compounds amid the azole family [1]. The pyrazole ring can provide solutions for pharmacodynamic and pharmacokinetic issues. Vast numbers of pyrazole derivatives exhibit a broad-spectrum of therapeutic effects including anti-bacterial, anti-convulsant, analgesic, antimicrobial, anti-inflammatory, antidiabetic, sedative, antirheumatic, anticancer, and antitubercular activities [2,3].

**Figure 1:**
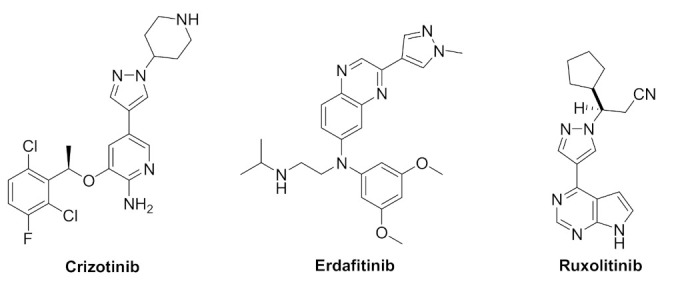
Structures of crizotinib, erdafitinib, and ruxolitinib.

The phosphorylation reactions were first discovered in glycogen metabolism and glycogen phosphorylase was identified in the 1960s [4]. Since then, kinases have been interesting therapeutic targets due to their involvement in a variety of cellular functions such as metabolism, cell cycle regulation, survival, and differentiation. A protein kinase is an enzyme that phosphorylates other protein substrates by chemically adding the terminal γ-phosphate group of adenosine triphosphate (ATP) to serine, threonine or tyrosine residues. Phosphorylation leads to a conformational change and thereby activates the functionality of the substrate proteins. There are >500 known protein kinases [5]. Indeed, deregulation of kinase function plays a fundamental role in cancer as well as immunological, inflammatory, degenerative, metabolic, cardiovascular and infectious diseases, and here arises the need for kinase inhibitors. Kinase inhibition is an interesting therapeutic avenue but clinical safety of the inhibitors must be considered [6]. There are tens of kinase inhibitors approved for marketing to date [6]: some of them bearing pyrazole moiety such as crizotinib [7], erdafitinib [8], and ruxolitinib [9] (Figure 1).

In this article, the recently reported pyrazole-based kinase inhibitors discussed in articles published during the past decade (2011–2020) have been reviewed. They have been classified according to their different kinase targets, and are presented herein according to the alphabetical order of the kinases’ names.

## 2. Pyrazole-Based Akt Kinase Inhibitors

### 2.1. Compound ***1***

**Figure 2:**
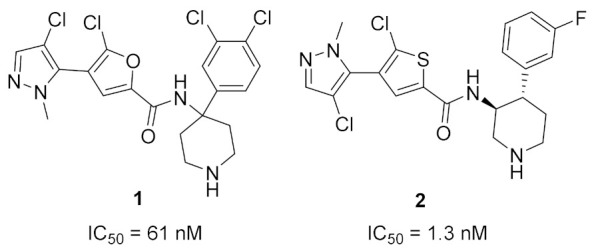
Structures of pyrazole-based Akt inhibitors and their IC_50_ values.

**Figure 3:**
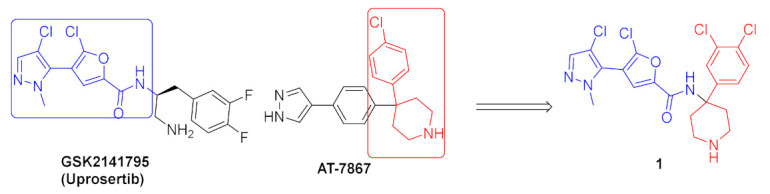
Structures of **G-SK2141795** and **AT-7867** and the hybridization to yield compound **1**.

Akt kinase, which is also named protein kinase B, is a serine-threonine kinase that is involved in PI3K-Akt-mTOR signaling pathway. This pathway is important for cell survival, apoptosis, proliferation, and metabolism. There are three known isozymes of Akt: Akt1, 2, and 3. Akt inhibitors are potential anticancer agents. Compound **1** (Figure 2) is a conformationally restricted analogue of GSK2141795 (uprosertib) (Figure 3), a known pyrazole-based Akt inhibitor. This was done with the aim of optimizing biological activity, selectivity, and metabolic stability. The structure of AT-7867 (Figure 3), another old pyrazole-based Akt inhibitor, inspired that research group to hybridize both structures in order to obtain compound **1** and its analogues. Compound **1** was tested against a 23-kinase panel and it exerted selectivity towards the Akt family. Its IC_50_ value against Akt1 is 61 nM, while the IC_50_ value of GSK2141795 is 18 nM. Western blotting showed that compound **1** decreased the level of phosphorylation of GSK3β in PC-3 cells, a substrate of Akt. In addition, compound **1** reduced the level of p-PRAS40 in LNCaP cells with IC_50_ equal to 30.4 nM, which is more potent than GSK2141795 (IC_50_ = 75.63 nM). Compound **1** demonstrated antiproliferative activity against HCT116 and OVCAR-8 cell lines (IC_50_ = 7.76 and 9.76 µM, respectively). Dichlorophenyl moiety was the best option for the highest biological activity. Any other halogen substitution other than the dichloro led to lower potency [10].

**Figure 4:**
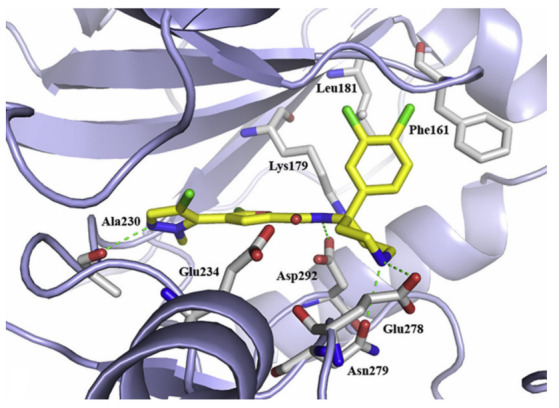
In silico binding interactions of compound **1** with Akt1 kinase crystal structure [10].

Docking of compound **1** into the crystal structure of Akt1 showed its position in the ATP binding site (Figure 4). The non-methylated nitrogen of pyrazole ring accepts a hydrogen bond from Ala230 backbone *NH*. The amide hydrogen donates another hydrogen bond to Asp292. In addition, the piperidine *NH* donates two hydrogen bonds to Glu278 and Asn279. Moreover, the dichlorophenyl ring occupies a lipophilic pocket under the P-loop and forms hydrophobic interactions with Phe161 and Leu181 [10].

### 2.2. Compound ***2***

**Figure 5:**
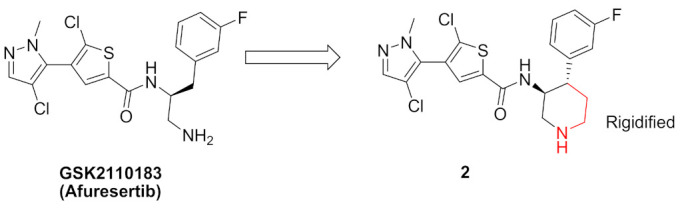
Structures of G-SK2110183 and the rigidification site to yield compound **2**.

Compound **2** (Figure 2) was designed as a rigid analogue of GSK2110183 (afuresertib) (Figure 5). Afuresertib is a pyrazole-based Akt1 kinase inhibitor whose K_i_ value is 0.08 nM. It possesses a flexible part in its structure, which was constrained in compound **2**. Its IC_50_ value against Akt1 is 1.3 nM. In addition, it showed antiproliferative activity against HCT116 colon cancer cell line (IC_50_ = 0.95 µM, 1.84-fold more potent than uprosertib). Compound **2** induced apoptosis in HCT116 cells and arrested their cell cycle at S phase. More interestingly, compound **2** demonstrated much higher potency over leukemia cell lines. For example, its IC_50_ values against MM1S, CEM-C1, and CCRF-CEM cell lines are 0.002, 0.007, and 0.008 µM, respectively. In the MM1S xenograft model, compound **2** could reduce tumor growth by 42%. Upon oral administration of 10 mg/kg of compound **2** in rats, its oral bioavailability is 52.5%. Moreover, compound **2** had a moderate inhibitory effect on hERG (40% inhibition when tested at 3 µM concentration). The SAR shows that the two chloro and one fluoro atoms in the structure of compound **2** are the best for activity. Replacement of any of them with any other group weakened the potency [11].

**Figure 6:**
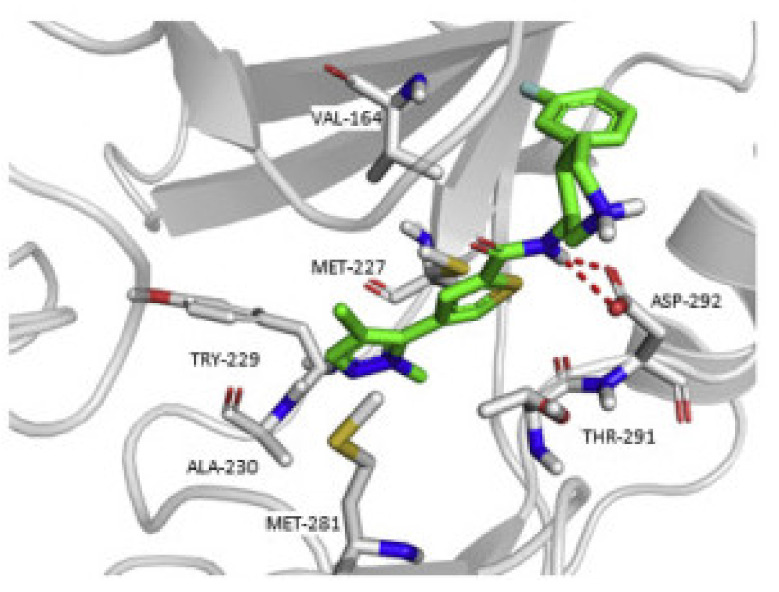
In silico binding interactions of compound **2** with Akt1 kinase crystal structure [11].

The putative binding interactions of compound **2** with Akt1 crystal structure are illustrated in Figure 6. The fluorophenyl ring occupies a hydrophobic pocket and forms hydrophobic interactions with Gly162, Val164, Lys179, and Leu181. In addition, the amide hydrogen donates a hydrogen bond to Asp292 [11].

## 3. Pyrazole-Based ALK Kinase Inhibitors

### Compound ***3***

**Figure 7:**
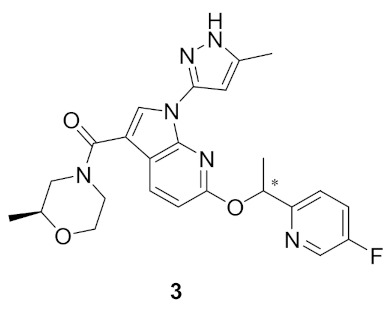
Structure of compound **3**.

**Figure 8:**
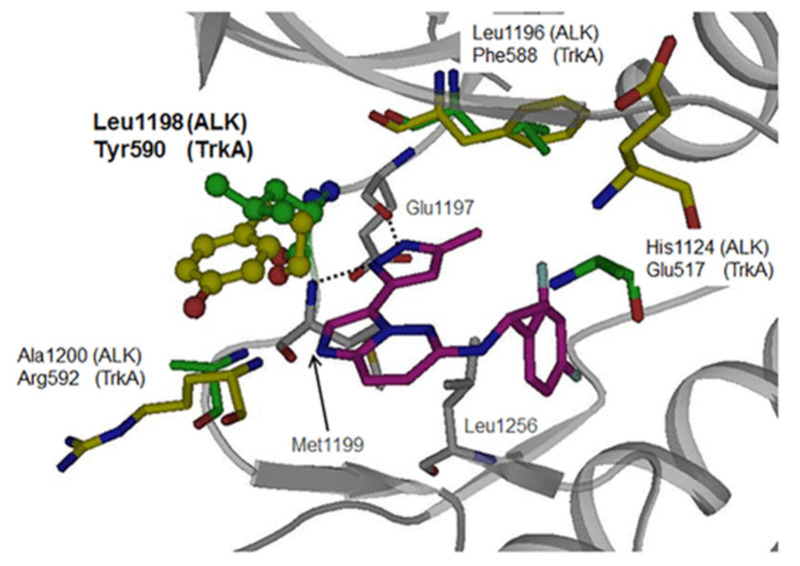
Cocrystal structure of compound **A** bound ALK protein. Different residues in ALK (green) and TrkA (yellow) around compound **A** (magenta) [12].

**Figure 9:**
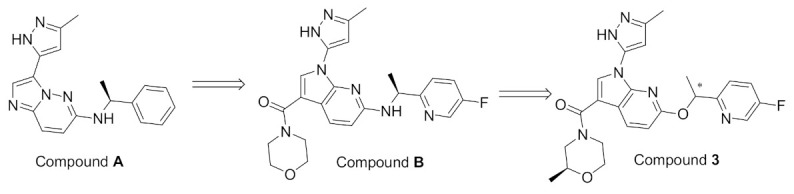
A schematic view of the development of compound **3** and the key changes to structure from the lead compound.

Inspired by the lack of brain-penetrant ALK inhibitor for elucidation of ALK’s role and mechanism in brain functions, Fushimi et al., developed compound **3** (Figure 7) [12]. The development stage started with lead discovery through high-throughput screening to find a potent brain-penetrant ALK inhibitor. The lead possesses imidazo[1,2-*b*]pyridazine scaffolded and with further SAR, compound **A** (Figure 8 and Figure 9), which had a potency comparable to crizotinib but lacked selectivity (potent TrkA inhibition and moderate kinase activity), was developed. To achieve selectivity to ALK over TrkA, a cocrystal structure of compound in ALK’s active site was generated. The cocrystal structure indicated an interaction between pyrazole and Glu1197 and Met1199 in the hinge region, the *N* at position 5 forcing the pyrazole to adopt a conformation optimal for interaction due to a steric clash with hydrogen at position 4 of pyrazole. The interaction between Leu1256 and imidazole ring, the pyridazine ring, and the 2,4-difluorophenyl group stabilized the L shaped confirmation (Figure 9).

**Figure 10:**
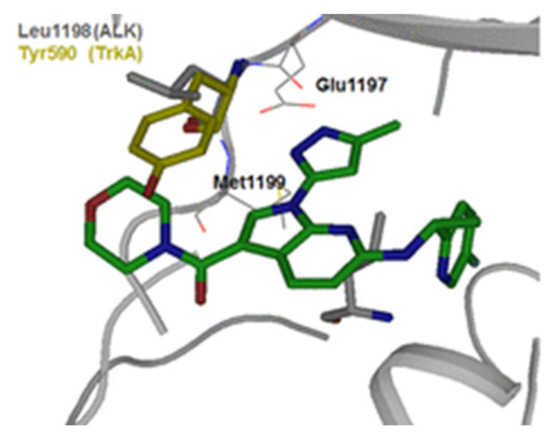
Cocrystal structure of compound **B** in ATP binding site of human ALK protein (gray). The relative position of Tyr590 based on crystal structure is shown in yellow [12].

Focusing on Leu1198 in the hinge region of ALK is a key towards selectivity against ALK than TrkA. The corresponding amino acid in TrkA is the bulkier Tyr590, which means narrower binding space. A substituent at position 1 of imidazo[1,2-*b*]pyridazine nucleus would be beneficial to access the Leu1198 region. Since there is no possibility of introducing a substituent, the imidazopyridazine nucleus of compound **A** was replaced with 1*H*-pyrrolo[2,3-*b*]pyridine. Moreover, sulfonyl and carbonyl moieties with varieties of substituents were introduced to position 3 in order to clash with Tyr590 of TrkA and achieve the desired selectivity. The modified series verified the hypothesis and revealed an improvement of selectivity to ALK over TrkA. Compound **B** with morpholinylamide group at position 3 (Figure 9) had an IC_50_ of 2.5 nM against ALK enzymatic assay and 23 nM in ALK cellular assay while having moderate inhibitory activity against TrkA (250 nM). To further develop the lead compound, a cocrystal structure of compound **B** with ALK aligned with TrkA was obtained (Figure 10).

Although compound **B** achieved the desired activity and selectivity on ALK, it was found that it had a high P-gp efflux (MDR1 BA/AB ratio = 18), most probably due to its increased polarity. Compound **3** has an excellent kinase profile with high selectivity and almost no activity against any kinase except focal adhesion kinase (FAK) at 100 nM (10-fold selectivity towards ALK over FAK). The IC_50_ of compound **3** against ALK kinase in cell-free and cellular kinase assays are 2.9 and 27 nM, respectively. CNS penetration was evaluated in mice and revealed brain concentration and plasma partition coefficient (K_p_) values being the highest 1 h post-administration. In addition, plasma protein binding and brain tissue binding calculated as fraction unbound were measured to be 0.08 and 0.017, respectively [12].

## 4. Pyrazole-Based Apoptosis Signal-Regulating Kinase Inhibitors

### Compound ***4***

**Figure 11:**
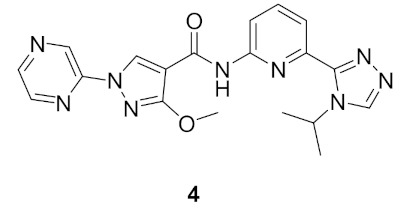
Structure of compound **4**.

**Figure 12:**
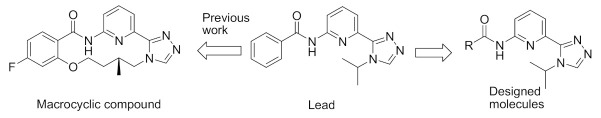
A scheme depicting macrocyclic compound, the lead compound, and a general structure of the target compounds [13].

Compound **4** (Figure 11) is a pyrazole derivative reported as ASK1 kinase inhibitor. ASK1 regulates both apoptosis and inflammation, and it has been involved in some diseases such as amyotrophic lateral sclerosis (ALS) and multiple sclerosis (MS). To study ASK1 modulation’s implication on neurodegenerative diseases, Xin et al., discussed the design and synthesis of ASK1 inhibitors based on a previous work that identified a macrocyclic compound (cell IC_50_ = 95 nM) obtained from the lead compound (IC_50_ = 607 nM, cell IC_50_ > 20 μM) (Figure 12). In this work, modifications on the distal phenyl ring were performed to improve potency of the lead towards ASK1. Docking of the lead into ASK1 revealed that the phenyl ring is placed in the solvent exposed area, and that explains the weak potency of the lead [13].

**Figure 13:**
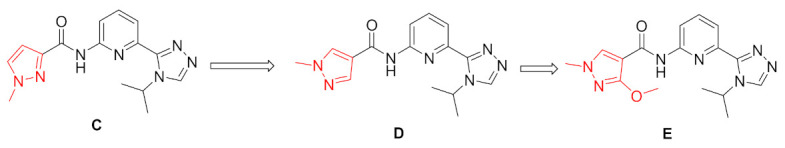
A scheme depicting compounds **C**, **D**, and **E**.

Potency was improved by replacing the hydrophobic phenyl ring with a more polar heterocycle such as the five-membered pyrazole. The first analogue, **C** (Figure 13) showed a 20-fold increase in potency compared to the lead compound (IC_50_ = 29 nM) on ASK1. Further investigations led to **D** (Figure 13), the positional isomer of **C**. Compound **D** was superior to **C** cell’s assay cell (**D**’s IC_50_ = 6.8 μM vs. **C**’s, cell IC_50_ > 20 μM). To mask the *NH* of amide, substituents were introduced on the pyrazole ring. The modification introduced in **C** induces an important conformational distortion. Introduction of methoxy had a dramatic effect on the potency. Compound **E** (Figure 13) had an IC_50_ of 90 nM on cell assay but had a low in vivo clearance in a rat PK experiment [13].

**Figure 14:**
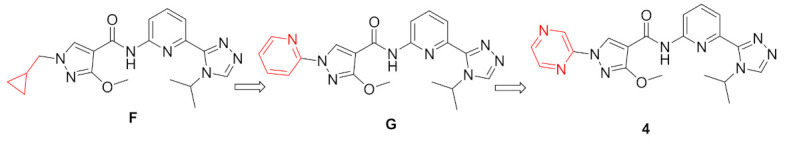
A scheme depicting compounds **F**, **G**, and **4**.

A number of modifications were done to improve the in vivo clearance, such as the macrocyclisation strategy which was employed in previous studies or reduction of the polar surface area (PSA). Results varied from improved potency but high in vivo clearance or high efflux ratio to loss of activity. The research group decided to increase the structural diversity of pyrazole compounds by adding different substituents on *N*’s pyrazole. The *N*-alkylated pyrazoles exhibited high potency (compound **F**, cell IC_50_ = 12 nM, Figure 14) yet all suffered from a high efflux rate. The *N*-pyridinyl derivatives showed a low efflux rate and acceptable potency (compound **G**’s IC_50_ = 299 nM, Figure 14). Continuous development and modification to the substituent attached to pyrazole led to the discovery of compound **4** (Figure 11 and Figure 14). It had a good balance of potency (cell IC_50_ = 138 nM) and an efflux rate of 5.0 [13].

## 5. Pyrazole-Based Aurora Kinase Inhibitors

### 5.1. Compound ***5***

**Figure 15:**
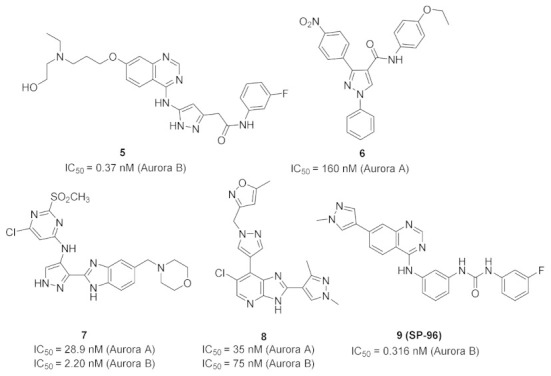
Structures of pyrazole-based Aurora kinase inhibitors and their IC_50_ values.

Aurora kinases are serine-threonine kinases that are involved in the mitosis process. Over-expression of Aurora kinases leads to cancer. Compound **5** (Barasertib, AZD1152) (Figure 15) is a highly selective Aurora B kinase inhibitor. Its IC_50_ value against Aurora B is 0.37 nM in a cell-free assay, which is over 3000-fold more selective toward Aurora B than Aurora A [14].

**Figure 16:**
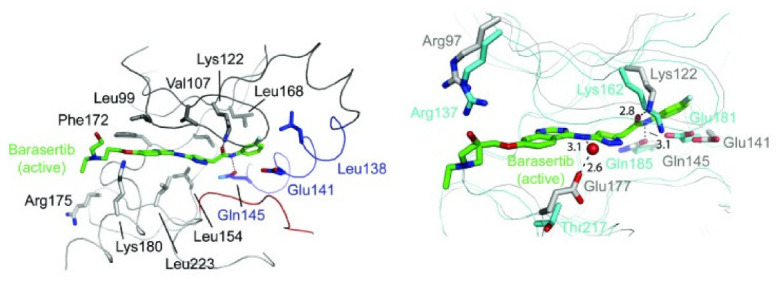
Binding interactions of compound **5** with Aurora B crystal structure [15].

The binding interactions of barasertib with Aurora B were studied by X-ray crystallography (Figure 16). It is reported that the compound occupies the interface between the small and the large lobes [15].

### 5.2. Compound ***6***

Li et al., have reported a series of pyrazole-based Aurora A kinase-inhibiting antiproliferative agents. Compound **6** (Figure 15) is the most promising among them. Its IC_50_ values against HCT116 colon cancer cell line, MCF7 breast cancer cell line, and Aurora kinase are 0.39, 0.46, and 0.16 µM, respectively. SAR study of this series showed that the nitro group is more optimal than hydrogen, methyl, methoxy, or chloro substituent [16]. A quantitative structure-activity relationship (QSAR) study on this series of compounds was carried out. It revealed that inclusion of bulky electron-withdrawing groups at *para* positions (in place of nitro and ethoxy) maximized the inhibitory potency against Aurora A kinase [17].

### 5.3. Compound ***7***

A series of pyrazolyl benzimidazole have been reported as antiproliferative agents possessing Aurora A/B kinase inhibitory effect. Compound **7** (Figure 15) is the most potent among this series. Its IC_50_ values against U937 (leukemia), K562 (leukemia), A549 (lung), LoVo (colon), and HT29 (colon) cancer cell lines are 5.106, 5.003, 0.487, 0.789, and 0.381 µM, respectively. In addition, it exerted strong potency against Aurora A and B (IC_50_ = 28.9 and 2.2 nM, respectively). The SAR showed that the morpholino ring is more favorable for activity than *H*, diethylamino, or piperidine. Docking of compound **7** into the crystal structure of Aurora A and B revealed binding into the active site. In the case of Aurora A, the benzimidazole ring forms hydrophobic interactions with a hydrophobic pocket formed by Ala213, Pro214, Leu215, and Gly216, and the hydrogen of *NH* donates a hydrogen bond to backbone amide of Ala213. The morpholino oxygen atom accepts a hydrogen bond from Arg137. In case of Aurora B, the pyrazole ring forms hydrogen bonds with *NH* of Ala173 and backbone carbonyl of Glu171. In addition, the pyrimidine ring forms two hydrogen bonds with backbone *NH* of Lys122. This rationalizes the stronger potency of compound **7** against Aurora B compared to Aurora A [18].

### 5.4. Compound ***8***

Compound **8** (Figure 15) is a dual inhibitor of Aurora A/B (IC_50_ = 35 and 75 nM, respectively). However, it is not very selective towards these two kinases. Upon testing at 1 µM concentration against a 105-kinase panel, it demonstrated more than 80% inhibition against 22 kinases. Compound **8** is potent against SW620 and HCT116 colon cancer cell lines (IC_50_ = 0.35 and 0.34 µM, respectively). The methylisoxazole moiety is more optimal than substituted phenyl in terms of potency and stability [19].

### 5.5. Compound ***9***

**Figure 17:**
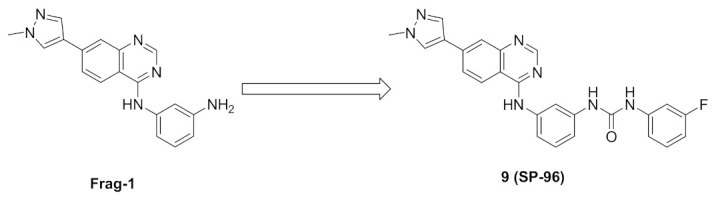
Modification to **Frag-1** to form compound **9** (**SP-96**).

Frag-1 (Figure 17) has been reported as an Aurora B inhibitor with an IC_50_ value of 116 nM. A docking study revealed a non-occupied pocket in front of the amino group (Figure 14). Lakkaniga et al., decided to extend the structure towards this vacant pocket in order to achieve better potency. This recently reported study led to the discovery of SP-96 (compound **9**) (Figure 15 and Figure 17). It is a very potent and selective non-ATP-competitive Aurora B inhibitor (IC_50_ = 0.316 nM). SP-96 is over 2000-fold more selective against Aurora B than FLT3 and KIT. Regarding the terminal fluorophenyl ring, halogen substituents are more tolerated than bulkier substituents. *Meta*-fluoro is more optimal than para or ortho positional isomers. The central phenyl ring in between urea and *NH* should be meta-disubstituted for selectivity against Aurora B. If *para*-disubstituted, the molecule inhibits Aurora B, FLT, and KIT. The pyrazole ring comes at the solvent exposure, which is why polar moiety at this place is more favorable than hydrophobic ones. Furthermore, attachment of a pyrazole ring at position 6 of the quinazoline ring is unfavorable for activity [20].

## 6. Pyrazole-Based BCR-ABL Kinase Inhibitors

### 6.1. Compound ***10***

**Figure 18:**
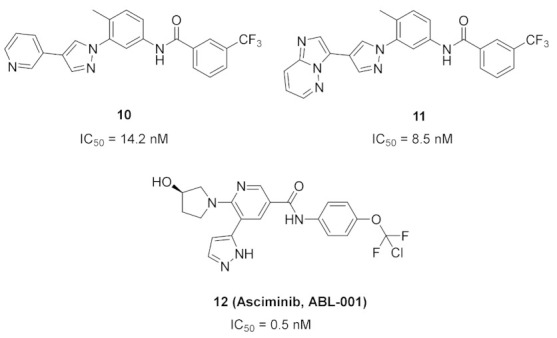
Structures of pyrazole-based BCR-ABL kinase inhibitors and their IC_50_ values.

**Figure 19:**
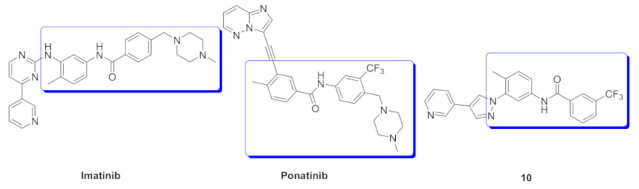
Structures of pyrazole-based BCR-ABL kinase inhibitors and their IC_50_ values.

Bcr-Abl inhibition is a potential therapeutic strategy for treatment of chronic myeloid leukemia (CML). Some pyrazole-based inhibitors of Bcr-Abl kinase have been reported in the literature during the last decade. In compound **10** (Figure 18), the diarylamide moiety was quoted from the structures of imatinib and ponatinib (Figure 19), known Bcr-Abl inhibitory anti-leukemia drugs. The IC_50_ values of compound **10** over Bcr-Abl kinase and K562 leukemia cell lines are 14.2 nM and 0.27 µM, respectively. Removal of trifluoromethyl group from its structure significantly decreased the potency [21].

**Figure 20:**
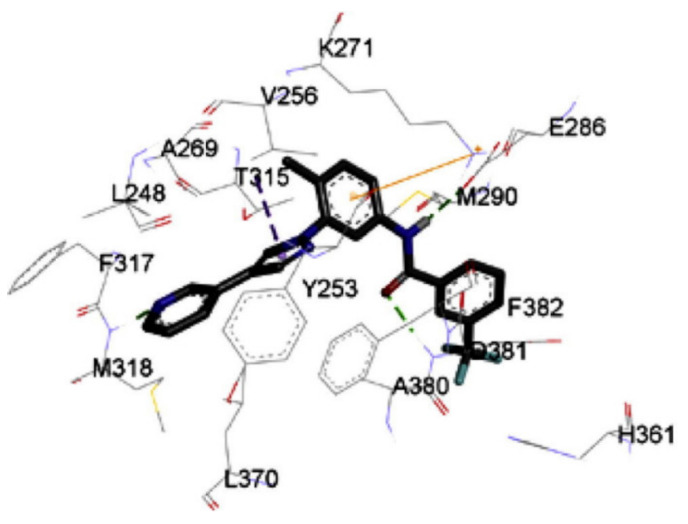
Putative binding mode of compound **10** with Bcr-Abl crystal structure [21].

Docking study of compound **10** into the Bcr-Abl crystal structure was performed to study its binding mode. The pyridine ring is the hinge region-binding moiety of this structure, similar to imatinib. The pyridyl nitrogen accepts a hydrogen bond from Met318. The amide linker forms two more hydrogen bonds with Glu286 and Asp381. Moreover, the pyrazole ring forms pi-pi stacking interaction with Thr315 (Figure 20) [21].

### 6.2. Compound ***11***

**Figure 21:**
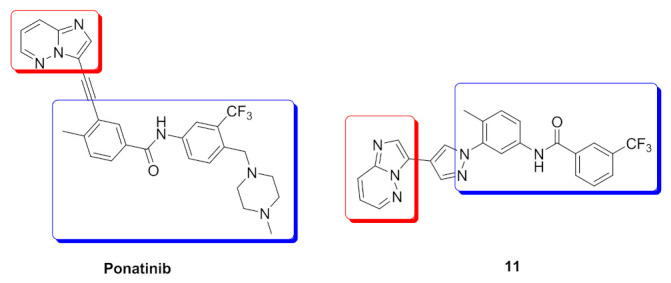
Modifications to ponatinib structure leading to compound **11**.

The same research group also reported compound **11** (Figure 18) possessing imidazo[1,2-*b*]pyridazine nucleus as a hinge region binder similar to ponatinib. Instead of the alkyne linker of ponatinib, compound **11** possesses a pyrazole ring similar to compound **10**. The imidazo[1,2-*b*]pyridazine nucleus was found more favorable for activity against Bcr-Abl kinase than the pyridine ring of compound **10** (Figure 21) [22].

### 6.3. Compound ***12***

Compound **12** (Asciminib, ABL-001) (Figure 18) is a non-ATP competitive inhibitor of Bcr-Abl kinase with a K_d_ value of 0.5–0.8 nM and an IC_50_ value of 0.5 nM. It is able to inhibit the T315I mutant Bcr-Abl as well (IC_50_ = 25 nM) [23,24]. Asciminib is a clinical candidate currently under clinical trials in patients with CML, alone or in combination with imatinib [25].

**Figure 22:**
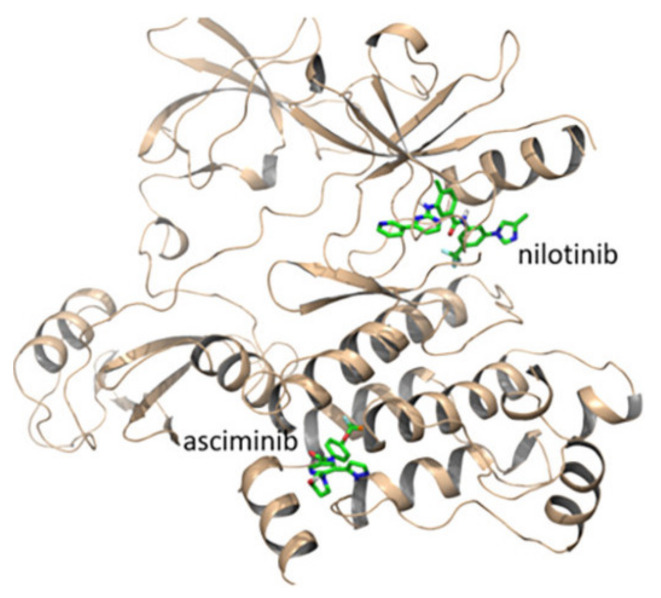
Comparison between the binding sites of asciminib and nilotinib [24].

An X-ray crystallography study of asciminib cocrystal with Bcr-Abl confirmed that it is an allosteric inhibitor, unlike nilotinib (Figure 22). The pyrazole ring forms a hydrogen bond with backbone carbonyl of Glu481 in addition to a hydrophobic interaction with Thr453. The chlorine atom forms the Van der Waals interaction with Leu448, Val487, and Ile508 [24].

**Figure 23:**
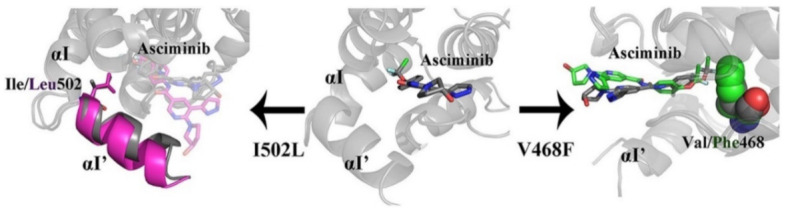
Effects of I502L and V468F mutations of Bcr-Abl kinase on the binding affinity of asciminib [26].

I502L or V468F mutations of Bcr-Abl kinase can lead to resistance of the leukemia cells to asciminib. Molecular dynamics studies revealed that I502L mutation changes the myristoyl pocket conformation while V468F shifts asciminib outside the myristoyl pocket (Figure 23). These mutations lead to declined binding affinity of the molecule with the kinase [26].

## 7. Pyrazole-Based Calcium-Dependent Kinase Inhibitors

### 7.1. Compound ***13***

**Figure 24:**
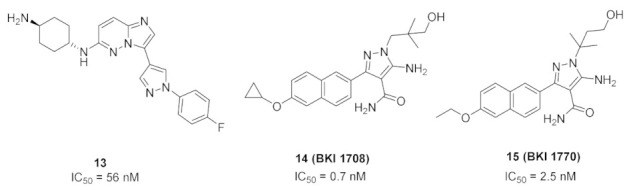
Structures of pyrazole-based calcium dependent kinase inhibitors and their IC_50_ values.

Compound **13** (Figure 24) has been reported as an inhibitor of *Plasmodium falciparum* calcium-dependent protein kinase 1 whose IC_50_ value equals 56 nM. This kinase is essential for a parasite’s life cycle stages of motility and to invade the red blood cells. Compound **13** showed anti-parasitic activity against *Plasmodium falciparum* with an IC_50_ value of 0.262 µM. The pyrazole ring is inserted in this structure instead of the 6-membered (hetero)aromatic rings to decrease logD and improve aqueous solubility and ADME profile. When incubated with human or mouse liver microsomal enzymes for 30 min, the remaining percentages of compound **13** were 80% and 84%, respectively. The SAR of this compound and its analogues revealed that fluoro is more optimal than cyano, and primary amino on the cyclohexyl ring is more favorable for activity than pyrrolidinyl or piperidinyl [27].

### 7.2. Compounds ***14*** and ***15***

*Cryptosporidium parvum* is a parasite that causes diarrhea in children all over the world. Its calcium-dependent protein kinase 1 is essential for its invasion and growth. Compounds **14** (BKI 1708) and **15** (BKI 1770) (Figure 24) have been recently reported as inhibitors of that kinase with anti-parasitic activity both in vitro and in vivo. The IC_50_ of both compounds against the kinase are 0.7 and 2.5 nM, respectively. In addition, EC_50_ values against the microbe are 0.41 and 0.51 µM, respectively. The naphthalene ring is more optimal for activity than any other heterocyclic fused bicyclic ring systems. BKI 1708 exerted in vivo efficacy against mouse model of cryptosporiodiosis when administered at 8 mg/kg once daily. It is safe up to 200 mg/kg with no tendency to induce cardiotoxicity. Similarly, BKI 1770 was efficacious at 30 mg/kg but twice daily, safe up to 300 mg/kg, and there is no cardiotoxicity liability [28].

## 8. Pyrazole-Based Checkpoint Kinase Inhibitors

### 8.1. Compounds ***16*** and ***17***

**Figure 25:**
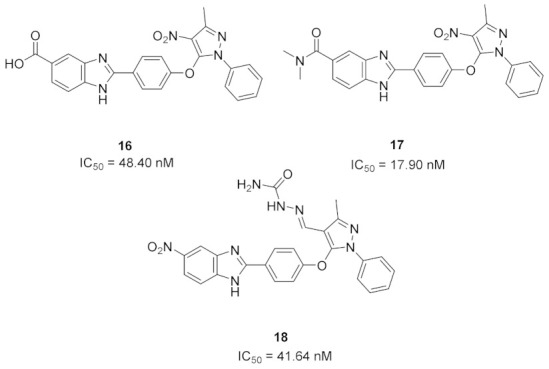
Structures of pyrazole-based checkpoint kinase 2 inhibitors and their IC_50_ values.

Checkpoint kinase 2 (Chk2) is involved in DNA damage response pathway. In addition, it is over-expressed by different types of cancer cells as it is essential for their survival. Chk2 inhibition is an avenue for cancer treatment. Galal et al., have reported a series of pyrazole-based Chk2 inhibitors. Compounds **16** and **17** (Figure 25) are examples of the most promising derivatives of that series. Their IC_50_ values against Chk2 in cell-free assay are 48.4 and 17.9 nM, respectively. In general, derivatives possessing amide moiety on the benzimidazole nucleus are more potent than carboxylic acid or nitro analogues. Furthermore, both compounds showed antiproliferative activity against HepG2 (hepatocellular carcinoma), HeLa (cervical), and MCF7 (breast) cancer cell lines but compound **17** is more potent (IC_50_ = 10.8, 11.8, and 10.4 µM, respectively). Both compounds induced cell cycle arrest in MCF7 cells, and both of them exerted synergistic cytotoxicity in vitro and in vivo, in combination with doxorubicin or cisplatin [29].

### 8.2. Compound ***18***

The same group that published on compounds **16** and **17** reported derivatives of them bearing semicarbazone moiety as inhibitors of Chk2. Compound **18** (Figure 25) is an example of this newer series (IC_50_ against Chk2 = 41.64 nM). It produced modest potency against HepG2, HeLa, and MCF7 cell lines with 2-digit micromolar IC_50_ values. Compound **18** alone arrested S phase of MCF7 cell cycle, while in combination with doxorubicin, it arrested G2/M phase. It exerted synergistic effect in vivo in breast cancer model in combination with doxorubicin [30]. It is noteworthy that the same group reported another series of Chk2 inhibitors possessing cyanopyrimidine instead of the pyrazole core and some of these derivatives showed improved potency [31].

## 9. Pyrazole-Based Cyclin-Dependent Kinase Inhibitors

### 9.1. Compound ***19***

**Figure 26:**
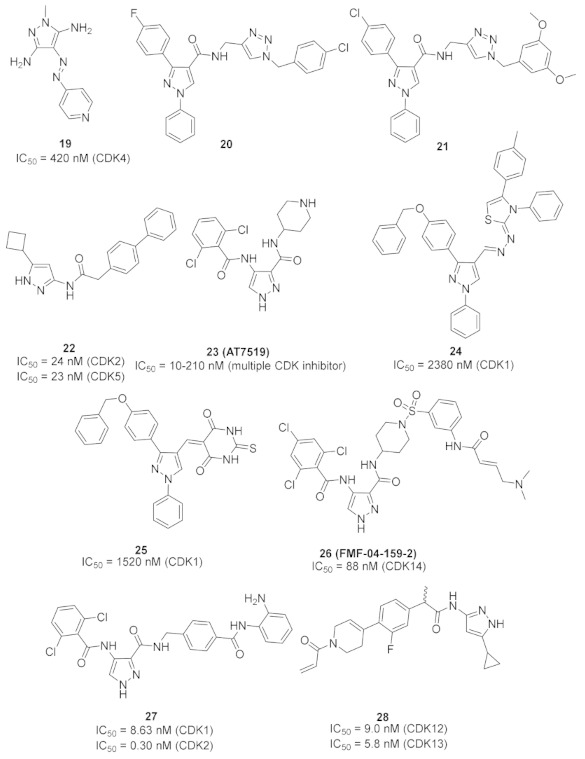
Structures of pyrazole-based CDK inhibitors and their IC_50_ values.

**Figure 27:**
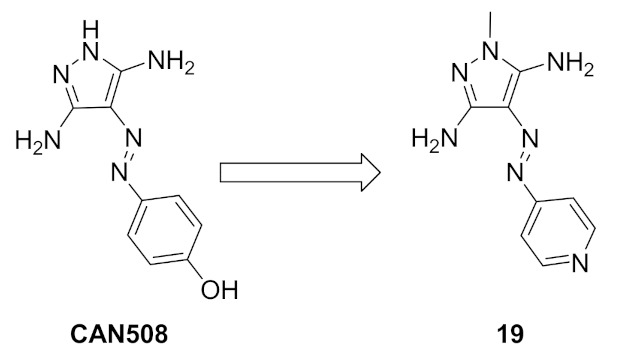
Structure of **CAN508** and the development of compound **19** from it.

Compound **19** (Figure 26) is an azo-diaminopyrazole derivative designed with similarity to CAN508, an old selective cyclin-dependent kinase (CDK)-9 inhibitor (IC_50_ = 350 nM) (Figure 27). The phenolic moiety of CAN508 was replaced with 4-pyridyl and the *NH* of the pyrazole ring was methylated. This led to alteration of the CDK selectivity profile of the compound. Instead of inhibiting CDK9 like CAN508, compound **19** is a selective CDK4 inhibitor with an IC_50_ value of 420 nM. It is more selective toward CDK4 than CDK1, 2, 7, and 9. *N*-Methylation was found more appropriate than *N*-acylation. Compound **19** was also tested for antiproliferative activity against K562, MCF7, and RPMI-8226 cancer cell lines but exerted modest activity. Its IC_50_ values are 67.4, 37.7, and 50 µM, respectively. It was further investigated for ability to induce apoptosis, and it happened in RPMI-8226 multiple myeloma cell line only [32].

### 9.2. Compounds ***20*** and ***21***

Compounds **20** and **21** (Figure 26) are the most promising CDK1-inhibitory antiproliferative agents among a series of pyrazole derivatives. Both compounds exhibited sub-micromolar IC_50_ values against MCF7 cells (IC_50_ = 0.13 and 0.15 µM, respectively), MIAPaCa pancreatic cancer cell line (IC_50_ = 0.28 and 0.34 µM, respectively), and HeLa cervical cancer cell line (IC_50_ = 0.21 and 0.73 µM, respectively). The SAR study indicated that when the R1 group is a monohalogen such as fluoro or chloro, the antiproliferative activity is higher than in the case of methoxy. Both compounds **20** and **21** induced cell cycle arrest in MCF7 cell line in G2/M phase in a dose-dependent pattern. Western blotting indicated that both compounds suppressed CDK1 expression in MCF7 cells at 50 and 100 nM concentrations. In particular, compound **21** completely inhibited its expression at 100 nM. In addition, both compounds induced apoptosis in MCF7 cells due to decreased mitochondrial inner membrane potential and increased reactive oxygen species formation [33].

**Figure 28:**
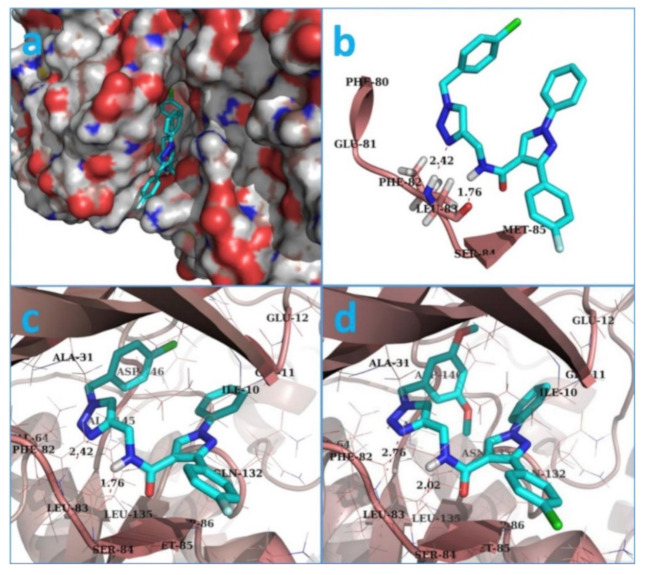
Putative bind interactions of compounds **20** (**a**–**c**) and **21** (**d**) [33].

Docking of both compounds **20** and **21** into the crystal structure of CDK1 was carried out (Figure 28). The pyrazole ring of both compounds interacts with Asp86 and Leu135. The *N*-phenyl ring interacts with Gly11, Glu12, and Gln132. The triazole ring interacts with Ala31, Gly81, and Leu83. In addition, the substituted benzyl ring forms hydrophobic interactions with Thr15, Val18, and Lys33. Moreover, the substituted phenyl moiety attached to position 3 of the pyrazole ring interacts with Ile10, Phe82, Ser84, Met85, and Lys89. The dimethoxy substituents of compound **21** form additional interactions with Asn133 and Leu134 [33].

### 9.3. Compounds ***22*** and ***23***

A series of 3,5-disubstituted pyrazole derivatives were synthesized and tested against pancreatic ductal adenocarcinoma cell lines. Compound **22** (Figure 26) is the most potent among them. It induced apoptosis in MiaPaCa2 cell line through a 3.2-fold increase in caspase-3/7 level. It was further tested for antiproliferative activity against MiaPaCa2 and four other pancreatic ductal adenocarcinoma cell lines, namely AsPC1, BxPC3, SUIT2, and S2-013 after a 3-day incubation period. Its IC_50_ values are 0.247, 0.315, 0.924, 0.209, and 0.192 µM, respectively. AT7518 (**23**) (Figure 26), an old pyrazole-based CDK inhibitor was the positive control in that assay. Its IC_50_ values against the same five cell lines are 0.411, 0.533, 0.640, 0.557, and 2.77 µM, respectively. The SAR study of compound **22** and its derivatives revealed that cyclobutyl is more optimal for activity than hydrogen, methyl, isopropyl, cyclopropyl, cyclopentyl, or phenyl. In addition, the biphenyl moiety is more favorable than naphthalene, ethylenedioxyphenyl, or dimethoxyphenyl. Compound **22** was also tested against a panel of fourteen kinases and exerted preference toward CDK2 and 5 (IC_50_ = 24 and 23 nM, respectively) [34].

It is noteworthy that AT7519 (**23**) is a multi-CDK inhibitory agent that inhibits CDK1, 2, 4, 6, and 9 with IC_50_ values ranging from 10 to 210 nM. It inhibits GSK3β as well with IC_50_ value of 89 nM. It induces apoptosis against different cancer types such as colon cancer and multiple myeloma [35,36].

### 9.4. Compounds ***24*** and ***25***

Compounds **24** and **25** (Figure 26) were reported as potent antiproliferative agents with CDK1 kinase inhibitory effect. They exerted strong potency against three hepatocellular carcinoma (HepG2, Huh7, and SNU-475), one colon cancer (HCT116), and one renal cancer (UO-31) cell lines. The IC_50_ values of compound **24** against these five tested cell lines are 0.05, 0.065, 1.93, 1.68, and 1.85 µM, respectively. In addition, compound **25** exerted IC_50_ values of 0.028, 1.83, 1.70, 0.035, and 2.24 µM, respectively against them. Both compounds inhibited CDK1 but with modest activity (IC_50_ = 2.38 and 1.52 µM, respectively). Moreover, the two compounds stimulated caspase-3 and induced HepG2 cell cycle arrest in G2/M phase [37].

### 9.5. Compound ***26***

**Figure 29:**
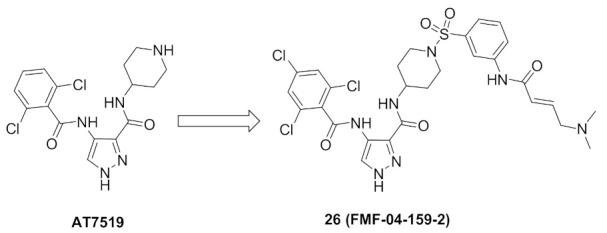
Structure of AT7519 and the development of compound **26** (FMF-04-159-2).

FMF-04-159-2 (compound **26**) (Figure 26) is an extended analogue of AT7519 (Figure 29) that possesses an α,β-unsaturated carbonyl moiety. That is why it acts as an irreversible inhibitor. It has been reported as an inhibitor of CDK14 kinase, a member of TAIRE subfamily of CDKs that includes CDK15-18, in addition to CDK14. The IC_50_ values of compound **26** against CDK14 in cell-free and whole-cell kinase assays are 88 and 500 nM, respectively. Moreover, it exerted antiproliferative activity against the HCT116 colorectal cancer cell line (IC_50_ = 1.14 µM). Although compound **26** is 8.6-fold less potent than AT7519 on the HCT116 cell line, it possesses the merit of improved potency and selectivity toward CDK14. The authors of this work recommend further structural optimization and investigation in order to optimize the kinase and cellular potency [38].

### 9.6. Compound ***27***

Compound **27** (Figure 26) has been recently reported as a dual inhibitor of CDK and histone deacetylase (HDAC). Its IC_50_ values against CDK1, CDK2, HDAC1, HDAC2, and HDAC3 are 8.63, 0.30, 6.40, 0.25, and 45.0 nM, respectively. In addition, it exerted high potency against HCT116 colorectal cancer cell line with sub-micromolar IC_50_ value of 0.71 µM. When tested on NIN3T3 normal cells, its IC_50_ value was 4.47 µM. So, its selectivity index is 6.3. The SAR study showed that o-dichlorophenyl moiety is more optimal than other substituents such as fluoro or methoxy. In addition, the aniline motif is the best solvent exposure moiety compared with other polar moieties such as hydroxamic acid. Compound **27** could induce apoptosis and stop the cell cycle of HCT116 at G2/M phase. In an in vivo HCT116 xenograft model in nude mice, compound **27** was intraperitoneally injected once daily for 22 days at doses of 12.5 and 25 mg/kg. It reduced the tumor size by 37% and 51%, respectively. In vivo PK evaluation of compound I following i.p. injection of 20 mg/kg showed the following parameters: t_1/2_ = 2.61 h, T_max_ = 2.00 h, C_max_ = 7570 ng/mL, and bioavailability = 63.6% [39].

**Figure 30:**
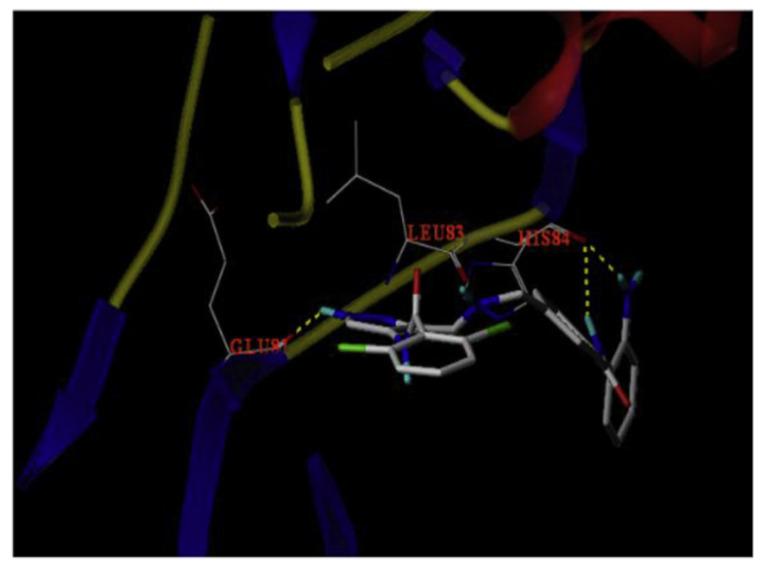
Docking pose of compound **27** into the crystal structure of CDK2 [39].

Docking of compound **27** into the crystal structure of CDK2 was performed in order to study its binding mode (Figure 30). The *NH* directly attached to the pyrazole ring forms a hydrogen bond as a donor with Glu81. In addition, the hydrogen atom of the carboxamide moiety attached to pyrazole at position 3 forms another hydrogen bond with Leu83. Moreover, the aniline *NH_2_* together with *NH* next to it forms two hydrogen bonds with His84 [39].

### 9.7. Compound ***28***

Compound **28** (Figure 26) is a patented pyrazole derivative claimed as selective CDK12/13 inhibitor. Its IC_50_ values against CDK12 and 13 are 9 and 5.8 nM, respectively. It is much less potent over CDK7 (IC_50_ = 880 nM). It possesses an α,β-unsaturated carbonyl moiety that is able to act as a covalent binder and irreversibly inhibit the kinases [40].

## 10. Pyrazole-Based EGFR Kinase Inhibitors

### Compound ***29***

**Figure 31:**
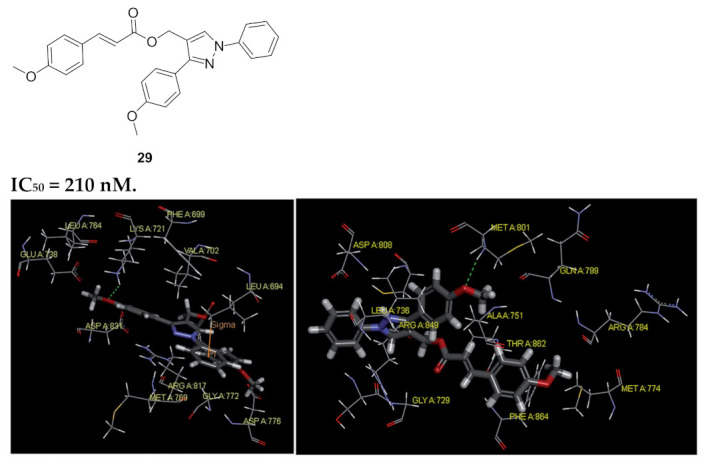
The structure of compound 29 and its IC50 value(**up**). Structure and molecular docking of compound **29** into EGFR (**left**) and HER2 (**right**). The dotted lines = hydrogen bond; yellow line = pi-sigma interactions [41].

Aiming at an anti-EGFR activity, a series possessing pyrazole scaffold was designed, synthesized, and evaluated. Compound **29** (Figure 31) presented itself as the most promising in the series, demonstrating an antiproliferative effect against MCF-7 breast cancer cell line (IC_50_ = 0.30 μM) and B16-F10 melanoma cell line (IC_50_ = 0.44 μM) tumor cells compared to erlotinib (MCF-7, IC_50_ = 0.08 μM and B16-F10, IC_50_ = 0.12 μM). Further biological evaluation to assess the potential inhibition of autophosphorylation of EGFR and HER-2 kinases using solid-phase ELISA assay was done. Compound **29** showed the highest inhibitory activity with IC_50_ = 0.21 ± 0.05 μM for EGFR and IC_50_ = 1.08 ± 0.15 μM for HER-2 kinases. In comparison, erlotinib demonstrated IC_50_ = 0.03 ± 0.002 μM against EGFR and IC_50_ = 0.14 ± 0.02 μM against HER-2 kinases. In the series, the trend of activity showed a preference towards electron donating groups rather than electron withdrawing ones on the distal phenyl ring as well the phenyl ring directly attached to pyrazole [41].

The molecular docking study of compound **29** with EGFR (Figure 31, left) and HER2 (right) showed compound **29** binding to the ATP binding pocket. In EGFR’s molecular docking, compound **29** bonded through hydrophobic interaction, an H-bonding between the methoxy of distal side chain reacted with Lys A721, and the unsubstituted phenyl ring interacted with Leu694 through pi-sigma interaction [41].

## 11. Pyrazole-Based FGFR Inhibitors

### 11.1. Compound ***30***

**Figure 32:**
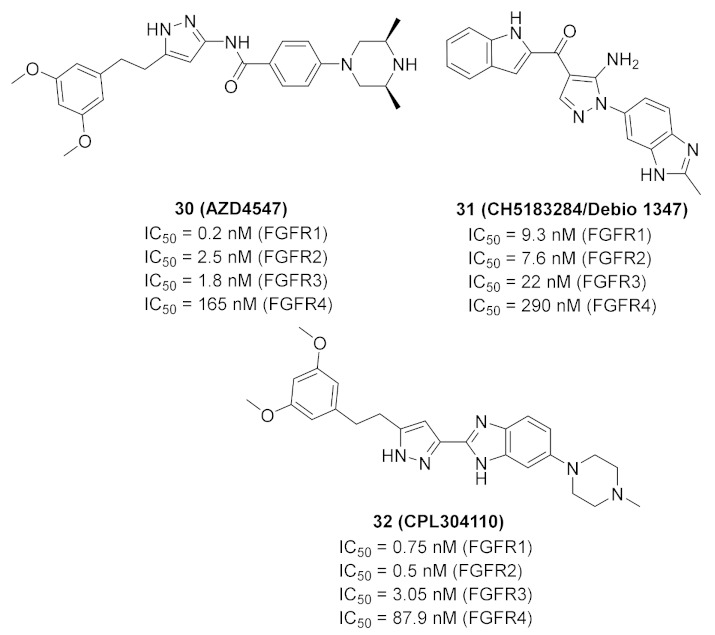
Structures of pyrazole-based FGFR inhibitors and their IC_50_ values.

Fibroblast growth factor receptor (FGFR) 1–4 kinases are over-expressed in different types of tumors. Compound **30** (AZD4547) (Figure 32) is a clinical candidate that possesses pan-FGFR inhibitory effect. It is orally bioavailable, well tolerated in vivo, and has exerted dose-dependent anticancer activity in tumor models [42]. It is currently under investigation in clinical trials in patients with lymphoma, glioma, lung, breast, gastric, and esophageal types of cancer [43].

**Figure 33:**
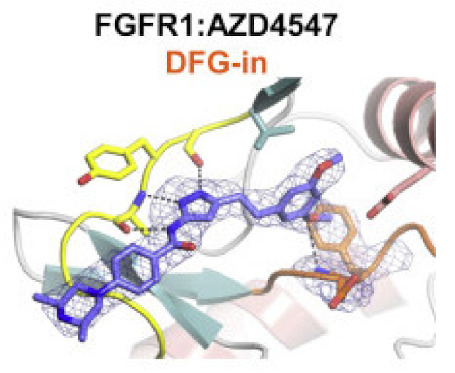
Binding mode of AZD4547 (**30**) with FGFR1 kinase crystal structure [44].

AZD4547 is a type I inhibitor of FGFR1, i.e., it binds to DFG-in conformation of the kinase. It forms four hydrogen bonds with pyrazole *N* and *NH*, amide *NH*, and methoxy oxygen (Figure 33) [44].

### 11.2. Compound ***31***

**Figure 34:**
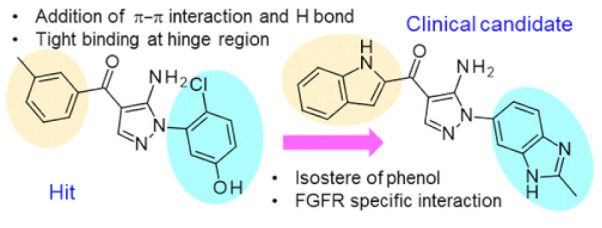
Hit-to-lead rational design of CH5183284 [46].

Compound **31** (CH5183284/Debio 1347) (Figure 32) is a pan-FGFR clinical candidate that was tested in one clinical trial and will be shortly tested in another two clinical trials against breast cancer and other solid tumors [45]. Its IC_50_ values against FGFR-1, -2, -3, and -4 are 9.3, 7.6, 22, and 290 nM, respectively. It is more selective against the FGFR family than the KDR and Src kinases (IC_50_ = 2100 and 5900 nM, respectively). Moreover, it exerted potential antiproliferative activity (IC_50_ values against gastric SNU-16 and colon HCT116 cancer cell lines are 17 nM and 5.9 µM, respectively). Its aqueous solubility equals 29 µg/mL, and its IC_50_ value against hERG is 6.9 µM. In addition, its hit-to-lead design rationale is illustrated in Figure 34 [46].

**Figure 35:**
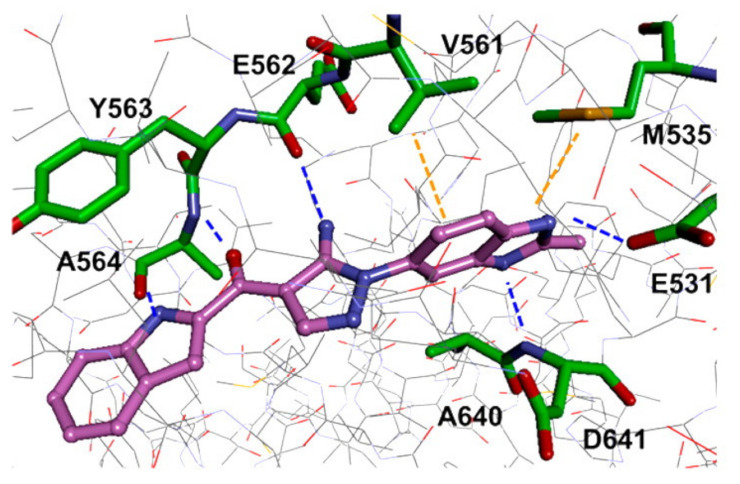
Putative binding mode of compound **31** with FGFR1 crystal structure [46].

Docking studies were conducted to understand the binding mode of compound **31** with FGFR1 and to rationalize its selectivity towards FGFR1 instead of KDR and Src. Its binding interactions are illustrated in Figure 35. The benzimidazole nitrogens form hydrogen bonds with E531 and D641. Moreover, the benzimidazole nucleus forms hydrophobic interactions with Val561, I545, and F641. It is more appropriate for activity than phenoxy or indole. The primary amino group donates a hydrogen bond to E562 while the ketone oxygen accepts a hydrogen bond from A564. Compound **31** is less potent against the KDR kinase because the L889 residue disrupts S-pi interaction with the benzimidazole nucleus. Similarly, the T314 and I339 of Src kinase do not interact with the molecule like V561 and V559 of FGFR1 [46].

### 11.3. Compound ***32***

Compound **32** (CPL304110) (Figure 32) is a clinical candidate that has been recently reported as a pan-FGFR inhibitor. Its IC_50_ values against FGFR-1, -2, -3, and -4 are 0.75, 0.5, 3.05, and 87.9 nM, respectively. When tested against a 13-kinase panel, it was selective against FGFR family only. It showed high potency against different cancer cell lines with over-expressed FGFR, and the highest potency was against FGFR-2-amplified SNU-16 gastric cancer cell line (IC_50_ = 85.64 nM). CPL304110 demonstrated an acceptable PK profile, low toxicity, and potent in vivo anticancer activity. Its *N*-methylpiperazinyl moiety is more favorable than morpholino, pyrazole, or urea. Compound **32** is currently under investigation in phase I clinical trials to test its safety in patients with bladder, gastric, or lung cancers [47].

Docking into FGFR-1 crystal structure showed the following interactions: (i) Benzimidazole *NH* donates a hydrogen bond to carbonyl oxygen of Ala564; (ii) Pyrazole *N* accepts a hydrogen bond from *NH* of Ala564; (iii) Pyrazole *NH* donates a hydrogen bond to carbonyl of Glu562; (iv) Methoxy oxygen accepts a hydrogen bond from *NH* of Asp641 in the gatekeeper region; (v) *N*-Methylpiperazinyl is the solvent exposure moiety of this structure [47].

## 12. Pyrazole-Based IKK Kinase Inhibitors

### Compound ***33***

**Figure 36:**
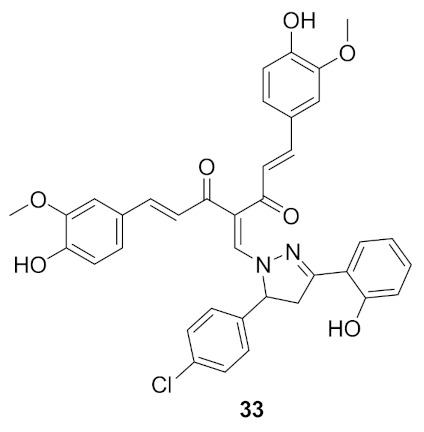
Structure of compound **33**, an IKK kinase inhibitor.

Curcumin derivatives were designed and synthesized for potential cytotoxic effect by targeting IKKβ, a sub-unit of IKK. The designed derivatives included the introduction of a substituted pyrazole ring to curcumin at methylene carbon. They were tested against the HeLa human cervical cancer cell line with curcumin and paclitaxel as positive controls. Compound **33** (Figure 36) showed an IC_50_ of 14.2 μg/mL which was more potent than curcumin (42.4 μg/mL) but less potent than paclitaxel (4.3 nM/mL). The investigations of the SAR of curcumin derivatives showed the importance of halogen atoms (4-chloro and 4-bromo) on activity and how they increase the activity compared to other derivatives. Derivatives with electron-withdrawing groups in the same position of the halogen were superior to their electron donating counterparts and the unsubstituted phenyl ring. An evaluation of the induction of apoptosis in terms of cleavage of the caspase-3 enzyme was performed. Compound **33** exhibited 69.6% of apoptosis, significantly higher than the 19.9% induced by curcumin [48].

**Figure 37:**
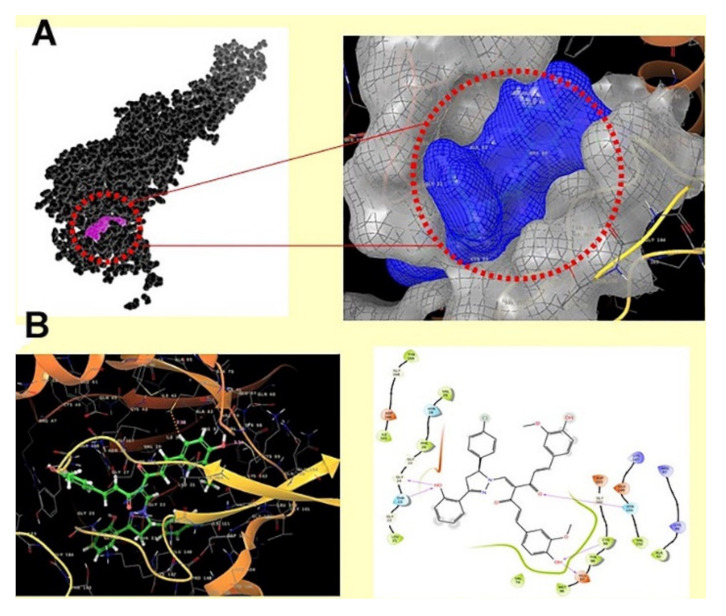
Compound **33′**s molecular docking complex with IKKβ. (**A**) Interaction complex of IKKβ with compound **33** (docking score = −11.874). Next to it the zoomed-in view of the interaction of compound **33** into binding grooves displayed in surface binding view. (**B**) 3D view of compound **33** (ball-stick view) binding mode with key amino acids (cartoon view). The 2D pose view is also shown next to it [48].

Molecular docking of compound **33** and other derivatives was performed with the crystal structure of IKKβ in the hinge region as the ATP binding site for favorable interactions (Figure 37). The co-crystallized inhibitor KSA was employed as the control ligand. Curcumin interacts with the gatekeeper residue Glu97, forms a hydrogen bond with Asp166, and has several hydrophobic interactions with the activation loop. Most of the designed compounds interacted with Glu97 and Cys99 residues, which was ATP’s adenine target during ATP’s interaction with the catalytic domain of IKKβ. Compound **33** interacts with Glu97 and Cys99 of the binding pocket of the receptor through the hydroxyl oxygen of curcumin’s backbone, while hydroxyl’s oxygen at pyrazole accepts hydrogen bonds from Gly24 and Thr23. Carbonyl oxygen of curcumin forms a hydrogen bond interaction with Asn150 [48].

## 13. Pyrazole-Based IRAK Inhibitors

### 13.1. Compound ***34***

**Figure 38:**
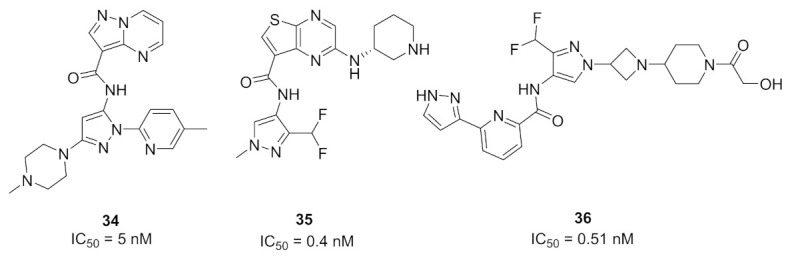
Structures of pyrazole-based IRAK4 inhibitors and their IC_50_ values.

The interleukin-1 receptor associated kinase 4 (IRAK4) is an intracellular serine-threonine kinase that is an upstream protein for IL-1R/TLR signaling pathway. This family of kinases includes IRAK1, IRAK2, and IRAK-M in addition to IRAK4. Inhibition of IRAK4 is a potential therapeutic target for treatment of inflammation. Compound **34** (Figure 38) has been reported as a potent and selective inhibitor of IRAK4 (IC_50_ = 5 nM). It was tested at 1 µM concentration against 108 kinases and only the IRAK4 was more than 80% inhibited. Replacement of the methyl group attached to the pyridyl ring with bulkier alkyl substituents decreased the potency. Moreover, replacement of the methyl attached to the piperazine ring with isopropyl or sulfonyl led to poor aqueous solubility. Likewise, replacement of the pyridyl and piperazinyl rings with two phenyl rings decreased aqueous solubility and weakened the potency against IRAK4 (IC_50_ = 690 nM). The aqueous solubility of compound **34** at pH 7 is 156 µM. At the cellular level, compound **34** exerted strong potency (IC_50_ = 83 nM) against the lipopolysaccharide-induced THP1-XBlue cells. It is also orally active in the mice antibody-induced arthritis model and inhibited cytokine release [49].

### 13.2. Compound ***35***

Compound **35** (Figure 38) has been reported as a potent IRAK4 inhibitor with an IC_50_ value of 0.4 nM. In addition, it exerted cellular activity against human peripheral blood mononuclear cells (hPBMCs) with IC_50_ value = 3 nM. Upon testing in MDCK cells, compound **35** was found to possess good permeability (25 × 10^−6^ cm/s). Replacement of the thienopyrazine nucleus with pyrazolo[1,5-*a*]pyrimidine led to a decreased permeability despite higher potency in a cell-free assay against the IRAK4 kinase. On the other hand, replacement of the thienopyrazine nucleus with pyrrolo[2,1-*f*][1,2,4]triazine or pyrrolo[1,2-*b*]pyridazine slightly increased the permeability but reduced the potency against IRAK4. Thus compound **35** is the most balanced derivative among these analogues with high potency and permeability [50].

### 13.3. Compound ***36***

Compound **36** (Figure 38) is a recently patented compound that possesses an IC_50_ value of 0.51 nM against IRAK4. The series analogues are claimed for treatment of autoimmune diseases, inflammatory disorders, and cancer. Any substitution of the pyrazole *NH* or any modification of the alcoholic side chain attached to the piperidine ring led to reduced potency against IRAK4 [51].

## 14. Pyrazole-Based ITK Inhibitors

### 14.1. Compounds ***37*** and ***38***

**Figure 39:**
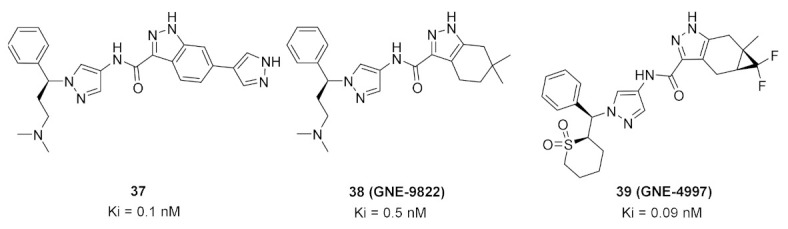
Structures of pyrazole-based ITK inhibitors and their Ki values.

**Figure 40:**
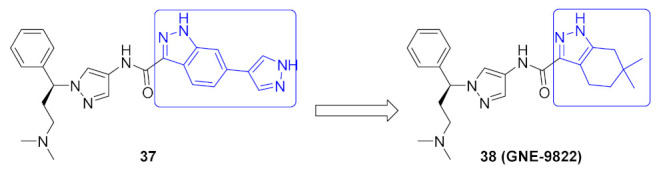
Development of compound **38** (GNE-9822) from compound **37** and the replacement of terminal 6-pyrazoloindazole with dimethyl-tetrahydroindazole.

The Interleukin-2 inducible T-cell kinase (ITK) is a member of the Tec tyrosine kinase family that is a T-cell signaling downstream of the T-cell receptor. Inhibition of ITK can help treat inflammatory disorders such as asthma. A research group at Genentech Inc. has reported the development and optimization of a series of pyrazole-based ITK inhibitors. In the beginning, they reported a series of indazole derivatives out of which compound **37** (Figure 39) is the most potent ITK inhibitor (K_i_ = 0.1 nM). Compound **37** inhibited the phospholipase C-gamma (PLCγ) kinase with a K_i_ value of 25 nM. Despite the strong potency of compound **37** against ITK, it suffers from poor PK properties. For example, it is orally unavailable in rats. In addition, it suffers from poor permeability in MDCK (0.3 × 10^−6^ cm/s). Furthermore, it is not a selective ITK inhibitor. When tested at 0.1 µM concentration over 218 kinases, it exerted more than 70% inhibition of 58 kinases [52]. The group decided to replace indazole with tetrahydroindazole and replaced the pyrazole ring attached to it with geminal dimethyl (compound **38**, GNE-9822, Figure 39) with the aim of improving kinase selectivity and ADME properties (Figure 40). GNE-9822 inhibits ITK with a K_i_ value of 0.7 nM. In addition, it is much more selective than compound **37**. At 0.1 µM concentration, it showed >70% inhibition of only six out of 286 tested kinases. Moreover, its K_i_ value against PLCγ equals 55 nM. The enantiomer of compound **38** is much less potent against ITK (K_i_ = 15 nM) and starts showing inhibitory effects against the Aurora **37** kinase (K_i_ = 170 nM). The permeability of GNE-9822 in MDCK improved significantly (4.6 × 10^−6^ cm/s) and oral bioavailability in rat increased to 40% following a 5 mg/kg dose (compared to 0% in the case of compound **37**) [53].

**Figure 41:**
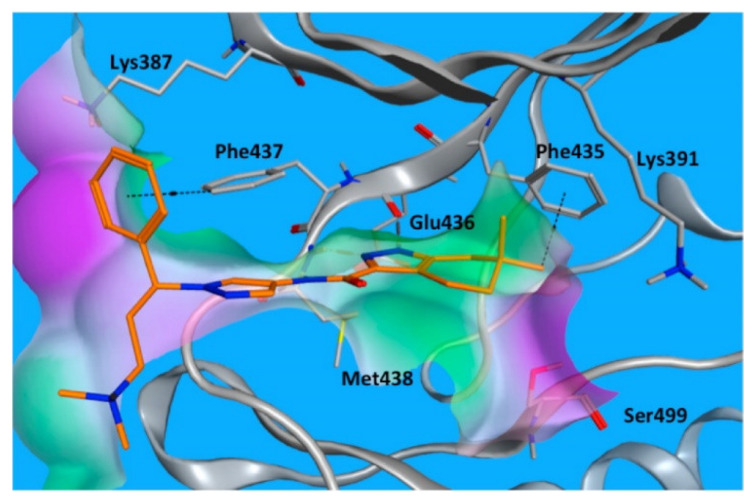
Co-crystal binding interactions of GNE-9822 (compound **38**) with ITK [53].

The real binding mode of GNE-9822 with the ITK kinase was studied by X-ray crystallography (Figure 41). One methyl group attached to the tetrahydroindazole nucleus interacts hydrophobically with Phe435. The benzylic phenyl also forms a hydrophobic interaction with Phe437. In addition, the tetrahydroindazole *NH* donates a hydrogen bond to Glu436 [53].

### 14.2. Compound ***39***

The same group of Genentech Inc. did further structural modification in order to enhance potency and selectivity, and at the same time reduce toxicity. They omitted the basic solubilizing moiety of the last series (dimethylamino-possessing side chain) and replaced it with cyclic sulfone. The less basic molecule **39** (GNE-4997) (Figure 39) exerted less toxicity than GNE-9822 (% inhibition values of hERG at 10 µM concentration are 6.8% and 88%, respectively). In addition, the potency of GNE-4997 against ITK increased significantly to reach a K_i_ value of 0.09 nM. Furthermore, GNE-4997 could reduce IL-2 and IL-13 production in mice. The 6-membered sulfone ring is optimal for potency against ITK compared to the corresponding 5-membered or open chain sulfone as well as the 6-membered sulfinyl (S=O) [54].

**Figure 42:**
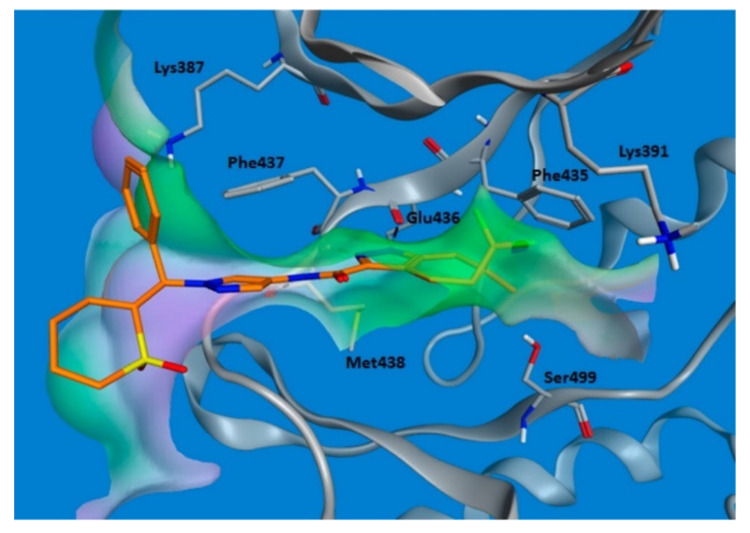
Co-crystal binding interactions of GNE-4997 (compound **39**) with ITK [54].

GNE-4997 bound with the ITK kinase cocrystal was studied by X-ray (Figure 42). Its amide hydrogen donates a hydrogen bond to Met438 carbonyl. The pyrazole ring anchors the molecule into the hinge region through the formation of two hydrogen bonds with *NH* of Met438 and carbonyl of Glu436. The cyclic sulfone ring plays a role in orientating the phenyl ring towards Phe437. Lastly, difluoromethylene occupies a hydrophobic pocket near the Phe435 gatekeeper residue [54].

In conclusion, GNE-4997 possesses several advantages over compounds **37** and **38**. It is a more potent ITK inhibitor, less toxic, with retained kinase selectivity. It may require further optimization in the future to improve its aqueous solubility, which is only 3.9 µM (17.4-fold less than GNE-9822) [54].

## 15. Pyrazole-Based JAK Inhibitors

### 15.1. Compounds ***40*** and ***41***

**Figure 43:**
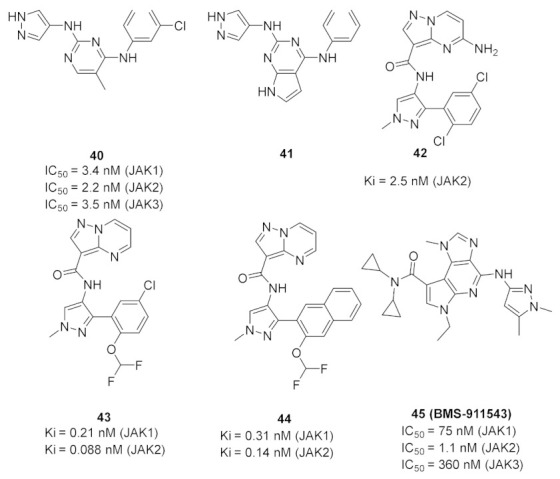
Structures of pyrazole-based JAK inhibitors and their IC_50_/Ki values.

**Figure 44:**
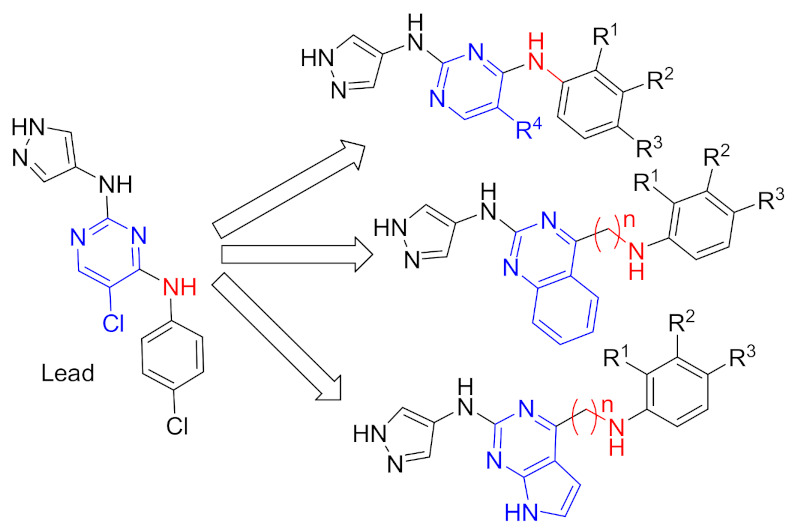
Schematic view of general designed compounds synthesized and tested.

A series of JAK inhibitors were designed and synthesized based on a lead compound from a previous work done by the same research group (Figure 43). Their previous finding showed that substitution on pyrazole’s *N* has no effect on activity, so by omitting the substituent on pyrazole’s *N,* focusing on the bioisosteric ring replacement of the central pyrimidine ring, and hoping to discover novel compounds with improved activity, three series were developed and compared based on their biological results. The three series were composed of three different central rings—a pyrimdine ring, a quinazoline fused ring and a pyrrolo[2,3-*d*]pyrimidine fused ring. Additional variations to the structures to investigate SAR were sought, such as changing the tether link’s length between the phenylamine derivative and the central heterocycle ring and having a different substituent on the distal phenyl ring (Figure 44) [55].

The different derivatives were tested in vitro against three JAK subtypes (JAK1,2 and 3) in a kinase assay at different concentrations, screened at 20 nM (as they were interested in activities in the nanomolar range only), and compared to positive control staurosporine (a prototypical ATP-competitive kinase inhibitor; IC_50_: JAK1 3 nM, JAK2 2 nM, JAK3 1 nM) and Ruxolitinib (an approved JAK inhibitor; inhibition at 20 nM: JAK1 97%, JAK2 99%, JAK3 95%). The quinazoline fused ring lost activity, and pyrrolo[2,3-*d*]pyrimidine had a moderate activity at 20 nM while pyrimidine-based derivatives showed high activity at 20 nM. Compound **40** (Figure 43) inhibited JAK1, 2 and 3 at 20 nM with an inhibition of 88%, 80%, and 79% respectively. The IC_50_ values measured for **40** on JAK1, 2 and 3 were 3.4, 2.2, and 3.5 nM, respectively. Activity dropped upon replacing the Chlorine atom on the pyrimidine ring with hydrogen or fluorine atoms [55].

**Figure 45:**
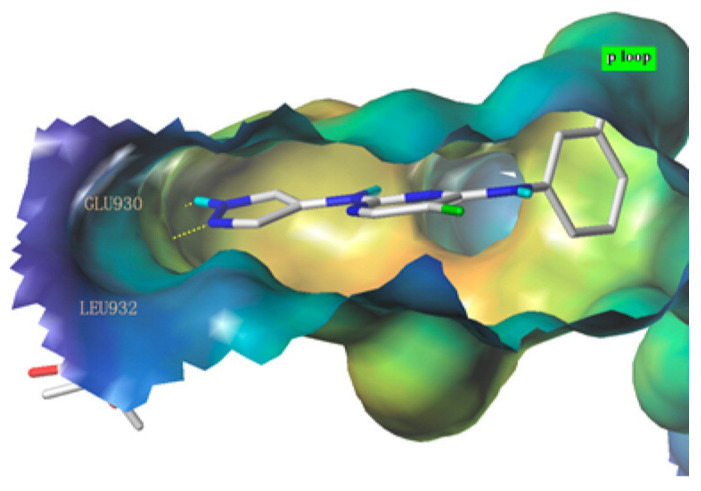
Docking of compound **40** with JAK2 crystal structure [55].

Next, derivatives were tested against the HEL (human erythroleukemia) cell line since the mutation JH2 pseudokinase domain of the Janus kinase 2 gene (JAK2 V617F) existed in it. Compounds were screened at 5 μM and the results were consistent with the trend seen in the kinase profile. The most active compounds (pyrimidine series and pyrrolo[2,3-*b*]pyrimidine series) were further tested against human prostate cancer PC-3, human breast cancer MCF-7, human erythroleukemia HEL, human myelogenous leukemia K562, and human lymphoid leukemia MOLT4 cell lines, while having ruxolitinib as the reference standard. Oddly, the pyrimidine series showed high antiproliferative activity against all cell lines tested (e.g., compound **40** IC_50_ against: PC-3 IC_50_ = 1.08 μM, MCF-7 IC_50_ = 1.33 μM, HEL IC_50_ = 1.08 μM, K562 IC_50_ = 0.77 μM, MOLT4 IC_50_ = 1.61 μM), while **41** showed remarkable selectivity to HEL (IC_50_ = 0.35 μM) and K562 (IC_50_ = 0.37 μM) (Figure 43). In addition to that, the compounds were inferior to ruxolitinib in kinase assay yet superior in cell-based assay, suggesting an off-target effect. To screen the off-target activity, two best representative compounds (**40** and **41**) were tested against multiple kinases. Compound **40** had an activity against Flt-3, VEGFR-2, PDGFRα, and TYK2 while **41** showed selectivity to JAK2 and 3 over the other tested kinases. These results explain the reason behind compound **40′**s activity against multiple cell lines and **41′**s activity against HEL and K562 cell lines. Docking of compound **40** in JAK2’s ATP binding pocket revealed the contribution by pyrazoles’ nitrogens in H-bonding with Glu930 and Leu932 (Figure 45) [55].

### 15.2. Compound ***42***

Philadelphia (Ph)-negative myeloproliferative disorders are a group of hematological disorders at the pluripotent hematopoietic stem cell level. These disorders include essential thrombocythemia, idiopathic myelofibrosis, and polycythemia vera. Activating mutation in JAK2 has a role in the progression of the disease by activating JAK-STAT signaling pathways [56].

Although a number of clinical candidates that are known small molecule JAK inhibitors for myeloproliferative disorder therapy are being clinically developed, and FDA approval of pan-JAK inhibitor ruxilitinib for the treatment of intermediate or high-risk myelofibrosis has confirmed and validated JAK as a clinical target for myeloproliferative disorders, no selective JAK2 inhibitor has been investigated yet. Ruxilitinib inhibits JAK1 and JAK2 equivalently, while the inhibition of JAK3 and Tyk2 is less pronounced. The JAK2 selective inhibitor will potentially improve the safety index; hence, chronic administration for the treatment of MRDs as well as decreasing the immunosuppressive side effects arising by inhibiting other members of JAK such as JAK1, JAK3, or Tyk2) is required. The degree of selectivity towards JAK2 over JAK1 is still unknown, but the group envisioned at least a 10-fold selectivity towards JAK2 over JAK1 for a potential biological activity [57].

**Figure 46:**
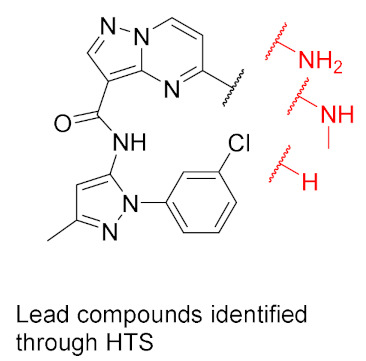
Depicting the structure of Pyrazolo[1,5-*a*]pyrimidine scaffold lead compounds discovered through HTS.

**Figure 47:**
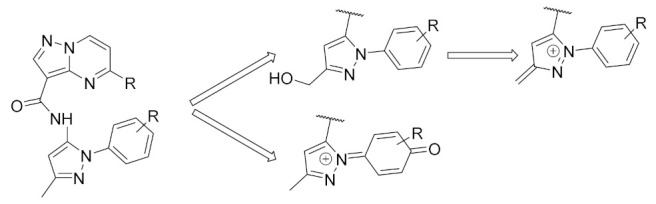
Schematic view of possible active metabolites formed in the 3-methyl-*N*-arylpyrazole core.

Discovery of the lead compound was achieved through high-throughput screening. The Pyrazolo[1,5-*a*]pyrimidine scaffold showed promising inhibitory activity against JAK2, with a nearly 10-fold selectivity to JAK2 over JAK1 and an even better selectivity against other members. Further lead optimization studies were performed to discover the selective, potent and orally active compound **42** (Figure 43). The lead compound, a 2-amino compound (Figure 46), showed a K_i_ of 2.5 nM inhibition against JAK2, potent inhibitory activity with an IC_50_ of 131 nM in a JAK2-driven SET2 cell-based assay through measuring the inhibition of pSTAT5, which is a downstream target of JAK2, low potential for reversible inhibition of five major human CYP450 isozymes, good in vitro permeability profile, moderate selectivity (~9–30×) against the other JAK family members and excellent selectivity when tested against a 177-kinase panel. Yet the lead had some limitations, the most important ones being low microsomal stability in five different species and poor thermodynamic aqueous solubility. In vivo pharmacokinetic profiling was done on rats and mice. The lead compound showed low plasma clearances in both mice and rats (3 and 6.8 mL/min/kg, respectively), extremely low V_d_ in both species (V_dss_ = 0.27 and 0.19 L/kg, respectively), and high plasma protein binding in both species (98.7% and 99.6%, respectively) which explained the low plasma clearance and low volume of distribution. The lead compound also had poor oral bioavailability in mice and rats (8.6% and 1%, respectively), which can be explained by poor aqueous solubility; hence, permeability was efficient. The group’s hypothesis for the poor aqueous solubility was due to high crystal packing forces hence multiple aromatic rings (four) and multiple potential hydrogen bond donors (HBDs) and acceptors (HBAs) exist in the structure of the lead compound, and not due to the high hydrophobicity of the compound, since the cLogP was 2.1. The two other lead compounds, the 2-des-amino compound and the 2-methylamino compound (Figure 47), were also investigated to explore the impact of removing HBD on oral bioavailability and other limitations shown by the 2-amino compounds. The 2-des-amino compound showed improved oral exposure compared to the 2-amino compound (F_oral_ = 44% vs. 1%); the improvement might be due to the greater kinetic aqueous solubility of 2-des-amino compared to the 2-amino compound hence both compounds showed similar thermodynamic solubility as well as similar permeability in MDCK cells. The rat plasma clearance and the V_d_ of the 2-des-amino compound was similar to the 2-amino compound. The activity in both the enzymatic assay against JAK2 and the cellular assay were nearly 10-fold less potent compared to the 2-amino compound. The 2-methylamino compound showed higher free fraction on rat plasma compared to 2-amino compound (1.7% vs. 0.4%), which expectedly showed a high clearance in rat microsomes compared to the 2-amino compound (48 vs. 39 mL/min/kg). The oral bioavailability of the 2-methylamino was higher compared to the 2-amino compound (F_oral_ (%) = 30 vs. 1) which can be explained by the improved kinetic aqueous solubility. The 2-methylamino compound was only 2-fold less potent compared to the 2-amino compound in the JAK2 enzymatic assay (K_i_ = 5.1 nM) [57].

Another limitation predicted by the group and one they decided to work on was the formation of potential reactive metabolites of lead compounds, hence lead compounds showed poor solubility as well as poor human liver microsomal stability. The 3-methyl-*N*-arylpyrazole center in the lead compound is especially critical in the formation of potential reactive metabolites, so the *N*-aryl moiety could be oxidized to paraquinoneimine, since the group hypothesized the role an electron rich aryl ring could play in contributing to the poor microsomal stability. In addition to that, the 3-methyl substituent is concerning since it can be oxidized in a two-step fashion into the pyrazoleiminium species through the oxidation of the 3-methylalcohol metabolite (Figure 47) [57].

**Figure 48:**
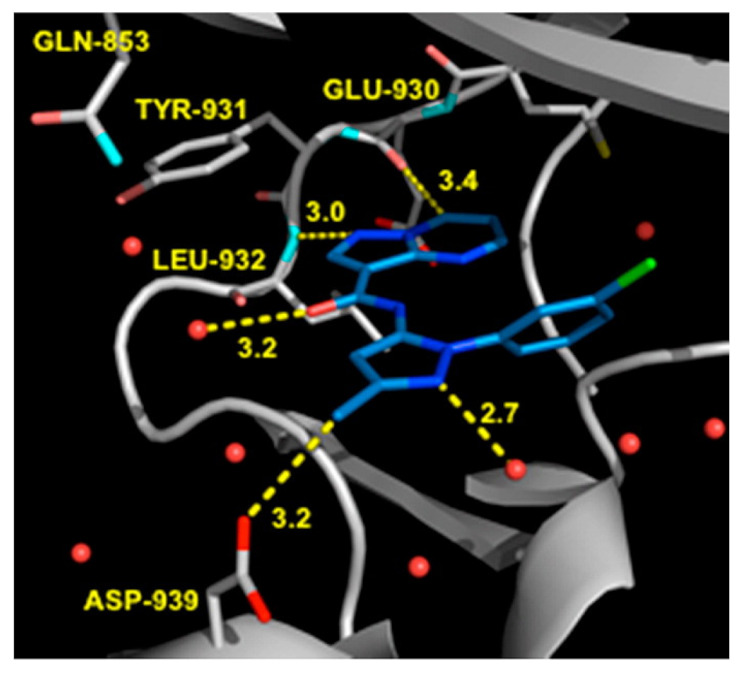
Co-crystal structure of 2-des-amino compound in the active site of the JAK2 kinase domain (2.3 Å). P-loop removed to allow a better view of the key active site interactions. Dashed lines = close contacts between ligand and protein with distances labeled in Å [57].

The 2-des-amino compound’s co-crystal structure with JAK2 revealed two bonds of a pyrazolo[1,5-*a*]pyrimidine core and the active sites which were a hydrogen bond between *N1* and Leu932 backbone’s *NH*, and a weak, non-classical, yet possible H-bond between *C7′*s *CH* and Glu930 backbone carbonyl (although the distance of H-bond is long (3.4 Å)). The phenyl ring occupies the hydrophobic sugar pocket of the ATP binding site. The amide’s carbonyl and *N2* of pyrazole formed H-bond with waters. The sequence homology of JAK1 and JAK2 is quite similar, yet some differences exist and can be targeted to discover a selective inhibitor. The group decided to exploit Asp939 in JAK2 (in close proximity to pyrazole’s methyl substituent), which is equivalent to Glu966 in JAK1, in order to improve selectivity between the two isoforms (Figure 48) [57].

**Figure 49:**
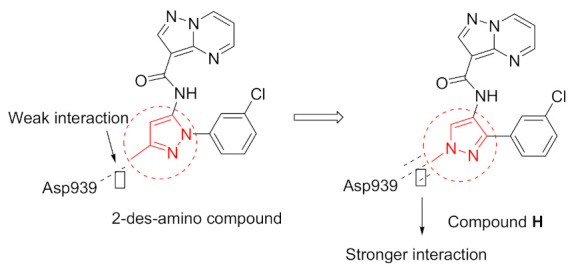
Schematic view of the steps in development and lead optimization.

Bioisosteric replacement of the 3-methyl-*N*-arylpyrazole moiety of the 2-des-amino compound with various pyridine analogues as well as regioisomers of pyrazole were prepared and tested. In summary, the pyridinyl compounds were active yet not as active as the pyrazoles. The most active compound was compound **A** (Figure 49) with a K_i_ of 3.2 nM and a selectivity of 8.1-fold towards JAK2 over JAK1, 36.5-fold towards JAK2 over JAK3 and 18.3-fold towards JAK2 over Tyk2. Compound **H**’s activity was superior to the other regioisomer of pyrazole, which can be explained by the high energy required for the amide and pyrazole groups to adopt a coplanar conformation that is caused by the lone pair repulsion of the *N2* of pyrazole and lone pair of the amide’s oxygen. In addition to that, the binding mode of the 2-des-amino compound showed an interaction between the *N2* of pyrazole and water. This interaction is lacking in regioisomeric pyrazole due to the absence of HBA in that position, while compound **H** retains such a position and bonding. The activity of compound **H** is superior to the 2-des-amino compound which might be due to higher polarization of the *CH* bond of *N*-methyl moiety of compound **H** compared to the *C*-methyl moiety in the 2-des-amino compound. The improved polarizability might improve bonding between the *N*-methyl group and Asp939. In terms of acidity, the 2-des-amino compound’s methyl is more acidic compared to compound **H** hence resonance stabilization is a factor when it comes to the resulting anion (calculated pKa 40 vs. 43 respectively). Yet the partial positive charge on the *N*-methyl moiety in compound **H** is greater because of the greater polarization. Rat plasma clearance of compound **H** was similar to that of the 2-des-amino compound (12.5 vs. 13.3 mL/min/kg). Oral bioavailability of compound **H** is higher in comparison to the 2-des-amino compound despite having poor kinetic and thermodynamic solubilities (both around 1 μM). A possible explanation given by the author is that compound **H** precipitated in crystalline form [57].

Compound **H**’s activity persuaded the group to investigate similar modification on the 2-amino lead compound. The modification led to a 2-fold increase in potency, a slight improvement of selectivity towards JAK2 over JAK1, and predictable clearance through rat liver microsomes, yet the oral bioavailability was still low (around 7%). The low oral exposure was explained by the poor kinetic and thermodynamic aqueous solubility. The group decided to replace the *N*-methyl moiety with various substituents in order to improve solubility and stability against human liver microsomes. Although some physiochemical and pharmacokinetic improvements were discovered, the potency was affected dramatically. The group decided to modify the distal phenyl ring. This modification led to the discovery of **42**. It had a K_i_ of 0.1 nM against JAK1, <10-fold selectivity over JAK1, JAK3, Tyk2 towards JAK2, an IC_50_ of 7.4 nM against pSTAT5 (in Jak2-driven SET2 cell-based assay), good oral bioavailability (F_oral_(%) = 63), which can be explained by low plasma clearance and high permeability, hence the solubility was poor and devoid of reversible CYP inhibition for the five major isoforms with only minimal time-dependent inhibition (TDI) of CYP3A4 (TDI IC_50_ = 5.8 μM with a 38% shift in AUC). Compound **42** was selective against a panel of 183 kinases (at 0.01 μM) and only inhibited 5 kinases outside the JAK family [57].

The excellent profile of compound **42** motivated the team to test on a SCID mouse the SET2 xenograft model that is dependent on JAK2 for growth, where the aim is to observe whether compound **42** can knock down the Jak2-mediated phosphorylation of STAT5. Sixty-four percent inhibition of pSTAT5 was observed at the 1-h time point at a dose of 100mg/Kg, and while the plasma concentration of compound **42** decreased, the inhibition of pSTAT5 also decreased [57].

### 15.3. Compounds ***43*** and ***44***

**Figure 50:**
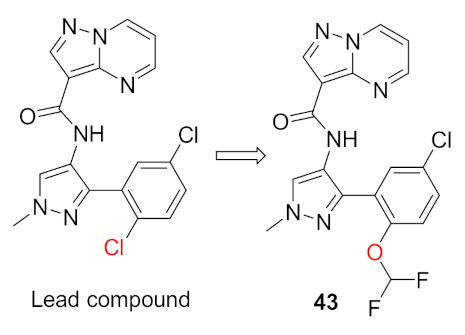
Schematic view the development of **43**.

Interleukin-13 (IL-13) is a cytokine implemented in various allergic inflammations, so it can be exploited for the treatment of diseases such as asthma and atopic dermatitis. IL-13 activates three isoforms of JAKs (JAK1, JAK2 and Tyk2), thus inhibiting the JAKs’ activity can prove clinically beneficial in the treatment of some allergic inflammation conditions. However, it is unknown which isoform is more important and has a bigger role in mediating the IL-13 effect. In this paper, the Genentech group that worked on compound **42** (Figure 43) explored the possibility of JAK1 inhibition in mediating and controlling the IL-13 effect. The group developed compounds **43** and **44** (Figure 43) through different rounds of SAR analysis. The lead compound was modified first at position 2 and when it was replaced with a different substituent, the most potent compound was compound **43** with the substituent’s difluromethoxy moiety (Figure 50) [58].

**Figure 51:**
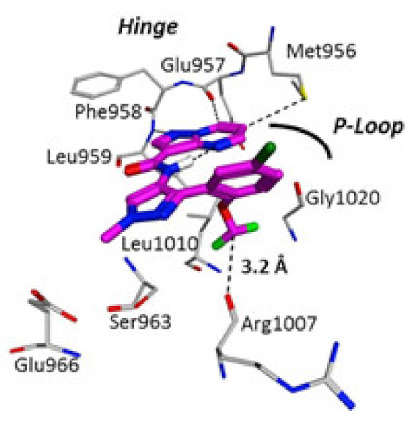
X-ray crystal structures of compound **43** in complex with JAK1 [58].

Compound **43** has a K_i_ of 0.21 nM against JAK1, K_i_ of 0.088 nM against JAK2, and exhibited an IC_50_ of 4.7 nM in IL-13 stimulated BEAS-2B cells monitored for pSTAT6 formation in the presence or absence of a JAK inhibitor (IL-13-pSTAT6 cell-based assay). In the X-ray crystal structure, the difluromethoxyphenyl moiety had Van der Waals interactions with Leu1010 and side chain methylene of Ser963. The fluorine atom forms dipolar interactions with the backbone carbonyl carbon of Gly1020. The polarized hydrogen atom of the difluoromethoxy group forms a non-classical hydrogen bond with the backbone carbonyl of Arg1007. The pyrazolopyrimidine core binds to the hinge region (Leu959 and Glu957) and interacts with the gatekeeper Met956 side chain, the *N*-methyl binds to Glu 966, and the chlorine atom interacts through Van der Waals interaction with the P-loop region (Figure 51) [58].

The next step undertaken was the modification of the heterocyclic tether (pyrazole) with the idea of improving co-planarity between the heterocycle and the amide. This modification led to a loss of activity. The last set of modifications was done to the distal phenyl moiety with a different substituent or by replacing the phenyl ring with other rings, leading to the discovery of compound **44**. Compound **44** had a K_i_ of 0.31 nM against JAK1, a K_i_ of 0.14 nM against JAK2, and an IC_50_ of 6.4 nM in the IL-13-pSTAT6 cell-based assay. To test the series’ selectivity, a sample compound was screened on a panel of 71 kinases and showed off-target activity against LRRK2 and FYN. The LRRK2 activity is concerning since it has a role in lung toxicity. Both compounds **43** and **44** were 47-fold and 83-fold more selective respectively towards JAK1 over LRRK2. Lastly, the series was tested for its metabolic stability and it exhibited poor to moderate stability against human liver microsomes. This series of compounds showed interesting biological activity and requires further development [58].

### 15.4. Compound ***45***

**Figure 52:**
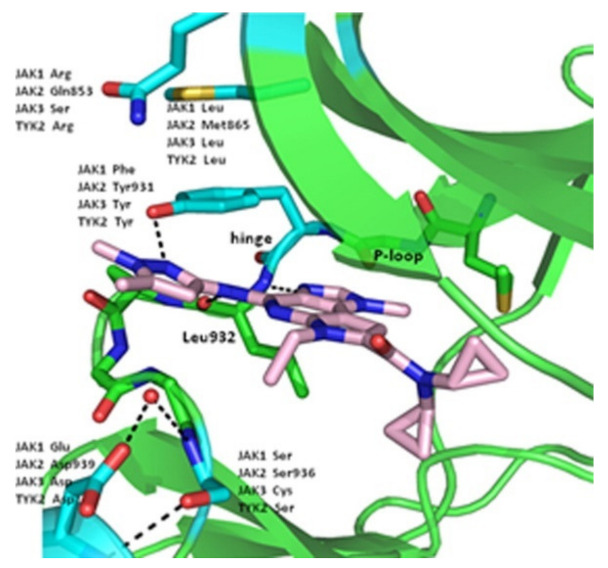
X-ray crystallized BMS-911543 (**45**) bound to the kinase catalytic domain of JAK2; Pick = BMS-911543’s Carbon; Green = JAK2’s carbon green; Cyan = residues near the C-4 group, which differ in the JAK family; dashed lines = hydrogen bonds [59].

The JAK2-V617F mutation activates JAK/STAT signaling pathway which has a role in the progression of myeloproliferative disorders MPD). Compound **45** (BMS-911543, Figure 43) was developed and discovered to be an inhibitor of JAK2. The group discussed the development from a lead compound which had a 4,5-dimethylthiazole ring instead of the pyrazole of compound **45**. The X-ray crystal structure of the lead compound showed an interaction between the *N* of pyrazole with the Tyr931 residue through H-bonding. It also showed unfavorable interactions of dimethyl moieties with non-conserved residues in the extended hinge region of other JAK family members, but this unfavorable interaction provided high selectivity which was desirable (Figure 52). The biggest drawback of the thiazole compound was its ADMET profile where it exhibited formation of reactive metabolites across species due to microsomal instability. Thiazole moiety of the lead compound was modified in its substituent or replaced with other heterocylces such as triazole and pyrazole. BMS-911543 was discovered and showed an IC_50_ of 1.1 nM against JAK2, an IC_50_ of 75, 360 and 66 nM on JAK1, JAK3 and Tyk2, respectively. The X-ray crystal structure of BMS-911543 was similar to the lead compound [59].

## 16. Pyrazole-Based JNK Inhibitors

### 16.1. Compound ***46***

**Figure 53:**
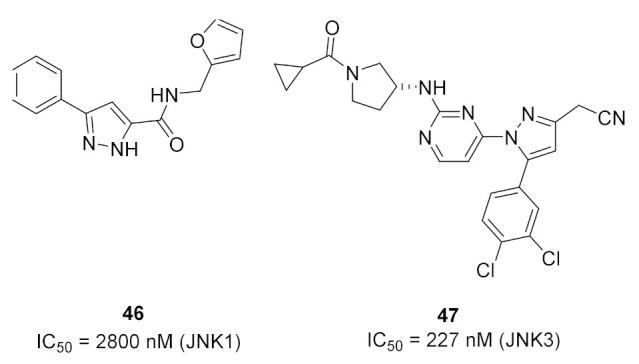
Structures of pyrazole-based JNK inhibitors and their IC_50_ values.

**Figure 54:**
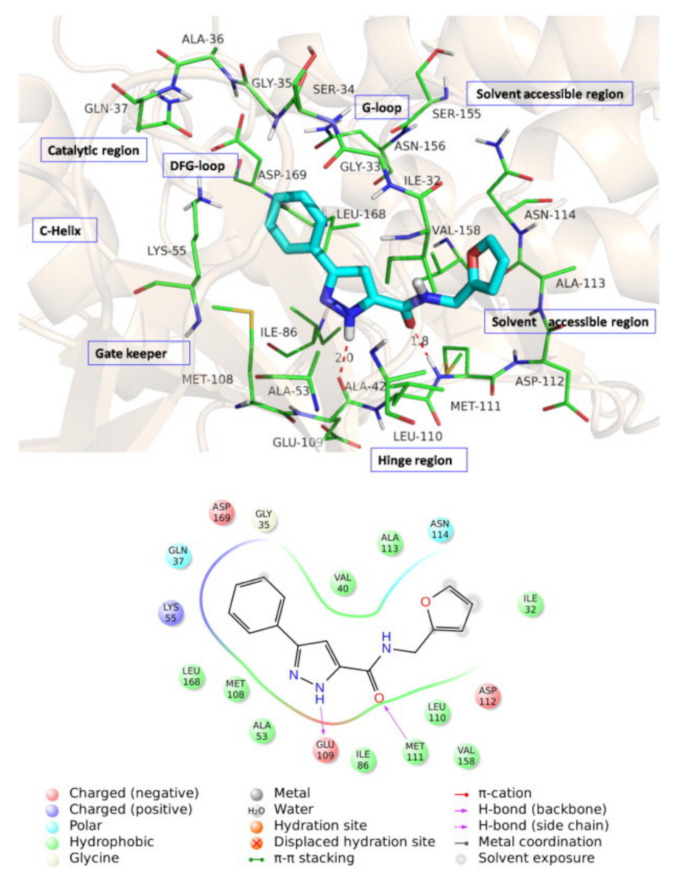
Docking pose and putative binding interactions of compound **46** with JNK-1 kinase crystal structure [60].

c-Jun N-terminal kinases (JNK) are MAPKs that play a crucial role in inflammatory disorders. Compound **46** (Figure 53) is the most potent JNK-1 inhibitor among a series of pyrazole carboxamide derivatives (IC_50_ = 2.8 µM). It was tested for in vivo anti-inflammatory activity against carrageenan-induced paw edema model in rats and reduced the paw inflammation by 73.11%, 81.81%, 91.89%, and 66.33% after 1, 2, 3, and 4 h following the injection. The furan ring of compound **46** was the most optimal for activity compared to the substituted phenyl, isoxazole, thiazole, pyridine, naphthalene, benzimidazole, or cyclopropyl. The docking study showed that compound **46** is an ATP-competitive inhibitor of the JNK-1 kinase. Its phenyl ring forms hydrophobic interactions with Val40 and Leu168. The pyrazole *NH* donates a hydrogen bond to the carbonyl backbone of Glu109, and the amide oxygen accepts a hydrogen bond from *NH* of Met111. The pyrazole ring faces Ile32, Leu110, and Val158 hydrophobic residues (Figure 54) [60].

### 16.2. Compound ***47***

**Figure 55:**
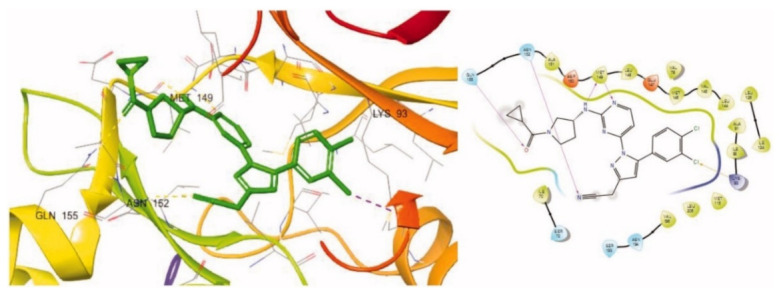
Putative binding mode of compound **47** with JNK3 crystal structure [61].

JNK3 is a potential target for neurodegenerative disorder therapy. Compound **47** (Figure 53) is a pyrazole-based selective JNK3 inhibitor whose IC_50_ value against the kinase is 227 nM. It was tested against a 38-kinase panel and was favored over JNK3. The SAR of this series indicated that 3,4-dichlorophenyl is more optimal for JNK3 inhibition than nitrophenyl, naphthyl, or other fused bicyclic rings. In addition, the nitrile group is more favorable than the primary amide. The docking study was carried out and the binding mode is illustrated in Figure 55. The aminopyrimidinyl is the hinge region-binding moiety of this structure. It forms two hydrogen bonds with Met149. The carbonyl oxygen of compound **47** accepts a hydrogen bond from Gln155. In addition, the cyano nitrogen accepts two hydrogen bonds from the backbone and side chain of Asn152. Lastly, the dichlorophenyl ring occupies a hydrophobic pocket and forms hydrophobic interactions. Its meta-chloro atom forms a halogen bond with Lys93 [61].

## 17. Pyrazole-Based LRRK Inhibitors

### 17.1. Compounds ***48***–***50***

**Figure 56:**
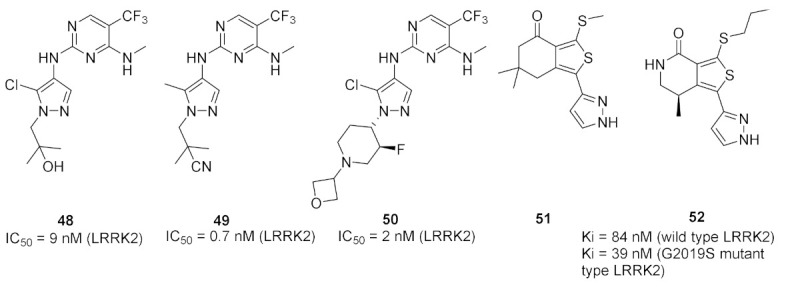
Structures of pyrazole-based LRRK inhibitors and their IC_50_/Ki values.

**Figure 57:**
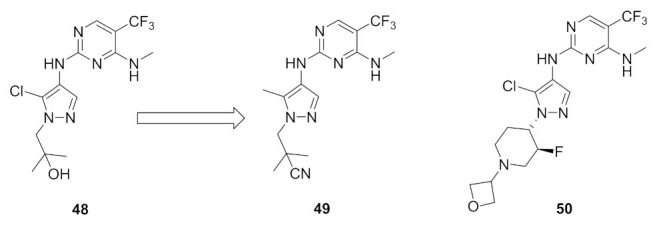
Development of compounds **49** and **50** from structural modification of compound **48**.

Leucine-rich repeat kinase 2 (LRRK2) is a potential target for treatment of Parkinson’s disease. Compounds **49** (GNE-0877) and **50** (GNE-9605) were developed via structural optimization of the solvent-exposed part of the ATP-binding site of compound **48** in order to improve human hepatocyte stability and brain exposure to the molecule, and to decrease the compound’s ability to inhibit or induce CYP (Figure 56 and Figure 57). The Ki values of compounds **48–50** against LRRK2 are 9, 0.7, and 2 nM, respectively. In addition, the IC_50_ values of the three compounds against pLRRK2 are 28, 3, and 19 nM, respectively. So the structural modifications done in compounds **49** and **50** led to improved potency. In addition, the brain-to-plasma ratio of compounds **49** and **50** in rats are 0.6 and 0.51, respectively, which is higher than that of compound **48** (0.37). Furthermore, the oral bioavailability of compounds **49** and **50** following 1 mg/kg administration in rats is 35% and 74%, respectively [62].

### 17.2. Compounds ***51*** and ***52***

**Figure 58:**
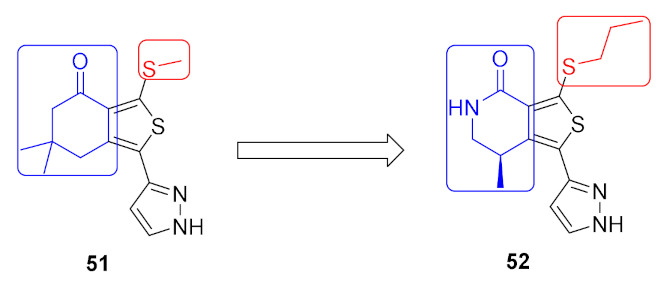
Development of compound **52** from **51** through introduction of amide lactam ring instead of cyclic ketone and extension of methylthio to propylthio.

Compound **51** (Figure 56) was identified through high throughput screening by the Merck company as an inhibitor of LRRK2. Structural optimization via insertion of the lactam led to improved CNS exposure and PK properties. In addition, the *N*-propylthio is more optimal for activity than isopropyl or other alkyl or cycloalkyl substituents attached to sulfur. Moreover, the methyl group attached to the lactam ring with this stereochemistry is the most favorable for activity compared to other alkyl substituents and other orientations (Figure 58). The K_i_ values of compound **52** (Figure 56) against the wild-type LRRK2 and the G2019S mutant-type LRRK2 kinases are 84 and 39 nM, respectively. Furthermore, the oral bioavailability of compound **52** is 98% after oral administration of 10 mg/kg in rats [63].

## 18. Pyrazole-Based Lsrk Inhibitor

### Compound ***53***

**Figure 59:**
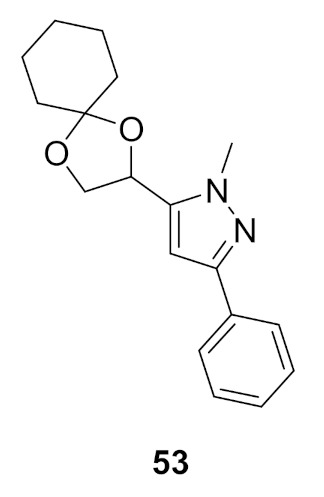
Structure of compound **53**, a pyrazole-based Lsrk inhibitor.

**Figure 60:**
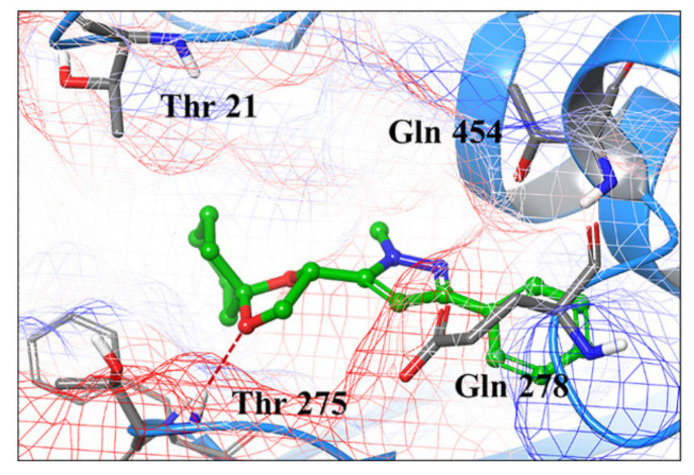
Docking pose of compound **53** into LsrK crystal structure [64,65].

(*S*)-4,5-Dihydro-2,3-pentanedione (commonly known as (*S*)-DPD) is a small signaling molecule that is phosphorylated by LsrK kinase. The resultant phosphor-DPD activates bacterial quorum sensing (QS). Thus, LsrK inhibition can interfere with QS and can help fix the problem of bacterial resistance. A series of 1,3,5-trisubstituted pyrazole derivatives were reported as LsrK inhibitors. Compound **53** (Figure 59) is the most potent among this series but with modest potency (IC_50_ = 119 µM). The SAR study indicates that the pyrazole core is more favorable for LsrK inhibition than pyridine or pyrimidine. Moreover, *N*-methylpyrazole is more optimal than unsubstituted pyrazole or pyrazole-bearing higher alkyl, cycloalkyl, or phenyl at *N1*. Unsubstituted phenyl at position 3 of the pyrazole ring is more optimal compared to substituted phenyl, heteroaryl, or cyclohexyl. The docking of compound **53** into the LsrK crystal structure showed formation of only one hydrogen bond between an oxygen atom with Thr275 (Figure 60) [64,65].

## 19. Pyrazole-Based MEK/ERK Kinase Inhibitors

### 19.1. Compound ***54***

**Figure 61:**
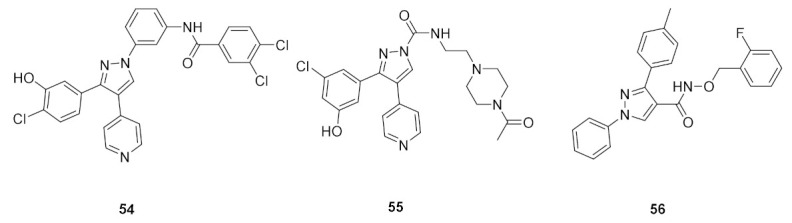
Structures of pyrazole-based MEK/ERK inhibitors.

The 1,3,4-triarylpyrazole scaffold was employed in designing a series of diarylureas and diarylamides linkers with different substituents, and a chloro hydroxy or methoxy substituent on the phenyl ring targeting hydrogen bonds in the active site. This series was tested for antiproliferative activity on the A375P melanoma human cell line. The amide linkers had higher potency compared to their urea counterpart, with compound **54** (Figure 61) as the most potent in the series (IC_50_ = 6.7 μM), surpassing the FDA-approved multi-targeted kinase inhibitor sorafenib (IC_50_ = 11.5 μM). To investigate the potential mechanism of action, compound **54** was tested in different concentrations (1, 3, and 5 μM) against the ERK-containing A375P cell lysate and compared with sorafenib. Both compound **54** and sorafenib decreased phosphorylation of ERK1/2 in a dose-dependent pattern [66].

### 19.2. Compound ***55***

Compound **55** (Figure 61) is part of a series consisting of a 3,4-diarylpyrazole center, with a variety of N-alkylcarboxamide chains. The inspiration behind designing and synthesizing this series was to target COX-2 and ERK1/2 simultaneously as suppressing both can have a desirable synergistic antiproliferative activity. Compound **55** exhibited an IC_50_ = 2.7 μM on MDA-MB-435 melanoma cell line. An investigation of the activity in suppressing the MEK/ERK pathway was performed using MEK/ERK-containing A375P cell lysate which was treated with three different concentrations (1, 3, and 10 μM) of test compounds including compound **55** and sorafenib for comparison. Compound **55** at 10 μM suppressed phosphorylation of MEK1/2 (inhibition percentage of 80.6%) and ERK1/2 (inhibition percentage of 87.5%) compared to sorafenib’s results (80.7% & 94.9%, respectively) in a dose-dependent manner. To explore the prospect of COX-2 inhibition using an enzyme immunoassay, test compounds including **55** were studied and compared to celecoxib. The best compounds were further tested on COX-1 to determine the selectivity profile between the two enzymes. Compound **55** inhibited COX-2 with IC_50_ = 0.30 μM (celecoxib had an IC_50_ value of 0.29 μM), did not show any inhibition of COX-1 up to 50 μM (also celecoxib) and had a selectivity index of 166.67, which is comparable to celecoxib’s selectivity index (172.41). Replacement of the hydroxyl group with methoxy diminished the activity. In addition, *N*-acetylpiperazinyl is more optimal than the corresponding analogues such as pyrrolidine, piperidine, morpholine, or dialkylamino. Furthermore, hydroxyl and chloro groups *meta* to one another are more favorable for activity than the *ortho*-disubstituted phenyl with the same two groups [67].

### 19.3. Compound ***56***

1,3-Diphenyl-*N*-benzyloxy-1*H*-pyrazole-4-carboxamide derivatives were synthesized and biologically evaluated. In vitro antiproliferative activity was measured using the MTT assay against three cancer cell lines (HeLa, MCF-7, and A549) and compared to gefitinib. Compound **56** (Figure 61) had the best results on the three cell lines with a GI_50_ of 1.18, 2.11, and 0.26 μM, respectively (gefitinib’s results were 1.52 μM, 6.71 μM, 2.86 μM). Moreover, the toxicity of the series was investigated against the human kidney epithelial cell 293T and compound **56** showed a median cytotoxic concentration of 20.57 μM. Further tests to assess MEK inhibition using the recombinant proteins of RAF–MEK–ERK cascade kinase assay revealed that compound **56** had the best activity with an IC_50_ = 91 nM versus an IC_50_ = 89 nM by the positive control U0126. In addition, the phosphorylation level of ERK was measured in a cell-based assay which predictably inhibited the activity of ERK phosphorylation in the B-RAF mutant cell line which showed an IC_50_ of 0.61 μM and had an excellent selectivity profile [68].

**Figure 62:**
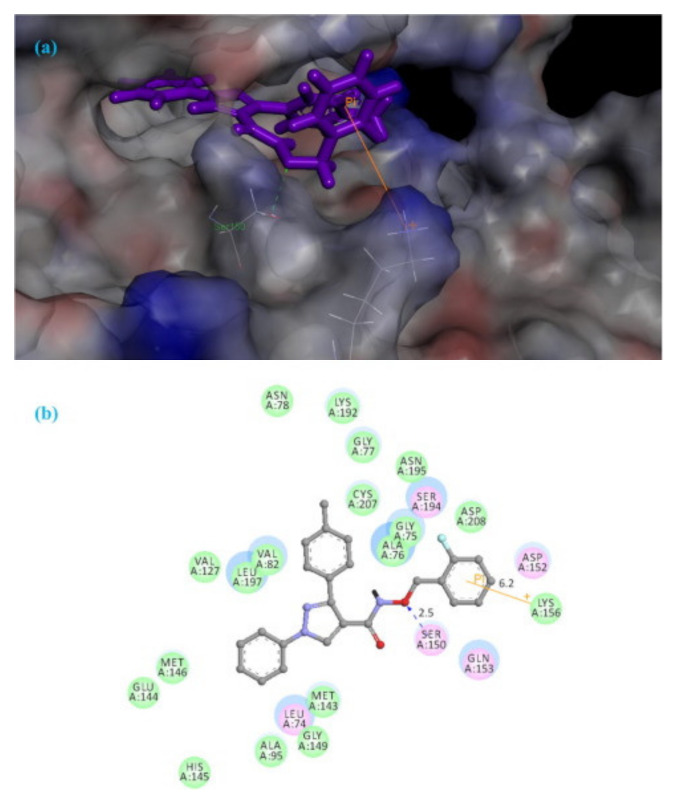
(**a**) Docking pose of compound **56** into the active site of the MEK1 protein-kinase. Hydrogen bond is illustrated as dashed line. (**b**) 2D binding mode of compound **56** with MEK1 active site [68].

Compound **56** was docked into the MEK1 active site to gain an insight of the binding interactions. The 1,3-diphenyl-1*H*-pyrazole scaffold occupied the ATP binding pocket deeply, showed a good shape complementarity, and exhibited hydrophobic interactions with multiple residues of the ATP binding pocket. On the other end, the chain with the aromatic end had a cation-pi interaction with Lys156 (Figure 62) [68].

## 20. Pyrazole-Based p38α/MAPK14 Kinase Inhibitors

### 20.1. Compound ***57***

P38α/MAPK1’s role in inflammatory diseases is the control and management of the production of cytokines (tumornecrosis factor-α (TNF-α), interleukin-1 (IL-1), interleukin-6 (IL-6), and interleukin-1b (IL-1b)), thus regulating the downstream signaling that mediates inflammatory response. Inflammatory disorders such as rheumatoid arthritis, inflammatory bowel syndrome, and psoriasis can be managed with p38α /MAPK1 inhibition [69].

**Figure 63:**
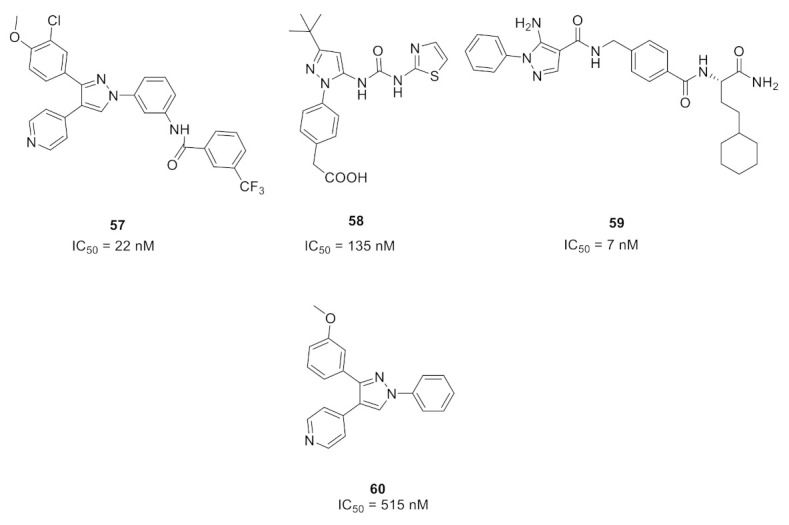
Structures of pyrazole-based p38α/MAPK14 kinase inhibitors and their IC_50_ values.

Based on that rationale, compound **57** (Figure 63) was discovered when a series of triarylpyrazoles where designed and synthesized as inhibitors of p38α /MAPK1. The series consisted of a pyrazole core, with three aryl arms around it. The phenol was designed as hydroxyl or methoxy derivatives, investigating the hydrophobicity as well as the potential of H-bonding. The *N*-phenyl arm was attached to the halogenated phenyl ring tethered with urea or amide linkers, testing the effect of the length of the tether as well as the possibility of extra H-bonding due to the additional *NH* group in the urea tether compared to amide [69].

The series was tested on a p38α /MAPK1 and a trend was noticed. The methoxy derivatives were superior to the hydroxyl derivatives, hinting at the presence of some hydrophobic pockets that can be accessed by a methoxy moiety. Another trend observed is the amide linker’s higher activity compared to that of urea linkers. The different substituent on the terminal phenyl ring also showed intolerability towards bulkier substituents, and trifluoromethyl substituted phenyl rings are better for activity. Compound **57** with an amide linker showed the highest potency in the series (IC_50_ = 22 nM). Further testing on a panel of 40 kinases at 10 μM to determine the selectivity of compound **57** was performed. Compound **57** exerted over 50% inhibition to A-RAF, B-RAF (wild type), B-RAF (V600E), RAF1, c-MET, and p38α and less than 50% on the other 36 kinases. To test the inhibitory activity of compound **57** against the p38α inside the cells, the NanoBRET target engagement assay was performed against the HEK293 cells with MAPK14-NanoLuc^®^Fusion VectorDNA. Compound **57** showed an EC_50_ of 0.52 μM, which is comparable to dasatinib’s 0.47 μM (positive reference), but lower than SB 203580’s 0.06 μM (another positive control which is a potent p38α /MAPK1) [69].

An additional test was performed to confirm the downstream inhibition of TNF-α production in lipopolysaccharide-stimulated THP-1 human cells. Compound **57** inhibited TNF-α production with an IC_50_ of 58 nM while SB 203580 had an IC_50_ of 20 nM. To investigate compound **57**’s properties, it was tested against hERG and showed less potency compared to E-4031, and 23.76 times more selectivity against the HEK293 cells (nanoBRETassay) than hERG. The plasma stability profile of compound **57** was also tested and revealed high stability in both human and rat plasma. Compound **57** was 100% unchanged after 30 min and decreased to 98.4% after 2 h, while procaine was 1.2% after 30 min and 0.2% after 2 h. The in vivo pharmacokinetic (PK) profiling of compound **57** at 10 mg/kg showed 11.32% oral bioavailability and high plasma stability. Lastly, compound **57** showed no gastric ulcerogenicity upon administration of 50 mg/kg once daily for 5 consecutive days in in vivo anti-inflammatory screening using carrageenan-induced paw edema model in rats compared to diclofenac through an intraperitoneal (i.p.) injection [69].

**Figure 64:**
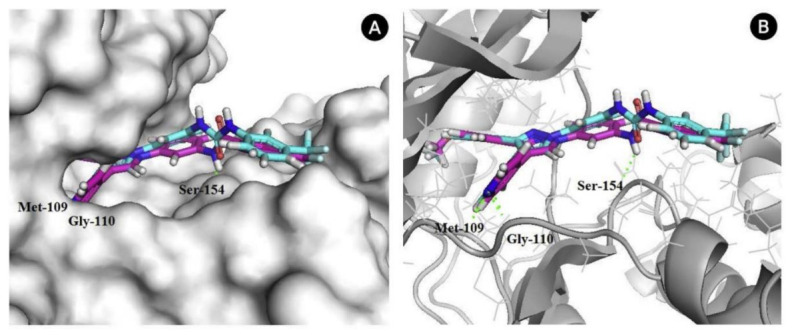
Binding mode of urea and amide derivatives; (**A**) binding mode of a urea compound (one of the compounds in the series, cyan) and **57** (magenta, amide), (**B**) best-docked pose for another two derivatives within the p38α kinase active site. Green dotted lines illustrate hydrogen bonding interactions [69].

The docking of this series with highly resolved X-ray crystal structures for the human p38α was performed to explain the activity. The docking results revealed that the tether was projected outward of the ATP binding pocket, and the *NH* of amide formed H-bonding with Ser-154 residue while the urea linker failed due to improper alignment. The halogenated phenyl ring at the end of the amide linker showed intolerability to bulky substituents due to the hindrance it exerts on the molecule to enter deep into the kinase active site. The pyridinyl’s *N* bonds with Gly-110 and Met-109 in the hinge region. The 3-chloro-4-methoxy motif is buried deep in the kinase active site (Figure 64) [69].

### 20.2. Compound ***58***

A series of *N*-pyrazole, *N*’-thiazole-urea derivatives were studied as p38α inhibitory agents. Compound **58** (Figure 63) is the most potent among this series with IC_50_ value of 135 nM against p38α. Despite its high potency against p38α, compound **58** is unable to inhibit in vivo phosphorylation of MK2, a well-known substrate of p38. This is attributed to poor cellular permeability of compound **58** because of the charged carboxylate group. Structural modification of the carboxylic acid group can lead to the optimization of pharmacokinetic and pharmacodynamic properties. The ethyl ester analogue of compound **58** could effectively inhibit phosphorylation of MK2 in HeLa cells with an IC_50_ value of 6 µM but its IC_50_ value against p38α is 639 nM. Further lead optimization can lead to analogues with improved characteristics [70].

### 20.3. Compound ***59***

**Figure 65:**
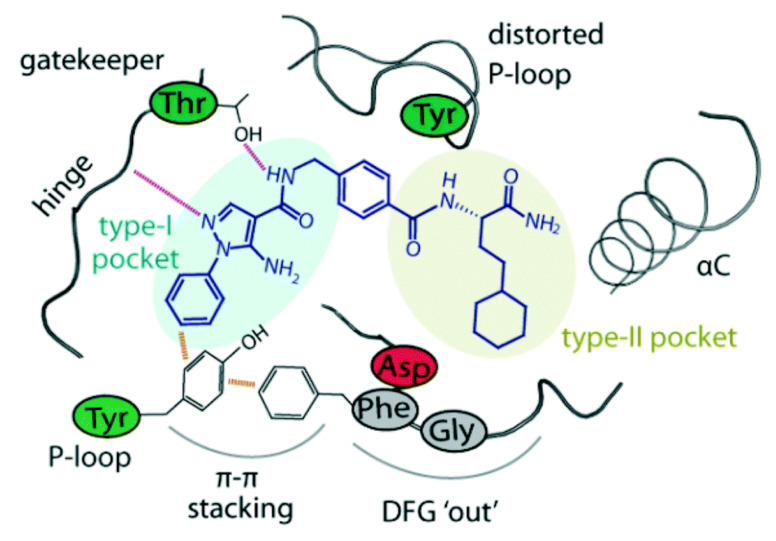
Binding mode of compound **59** with p38α crystal structure [72].

A library of DNA-encoded small molecules was studied for potential inhibitory effect against p38α. Compound **59** (VPC00628, Figure 63) is the most potent and selective molecule identified from 12,600,000 tested compounds. Its IC_50_ against p38α is 7 nM, and it was selected for further studies. Compound **59** is a type II kinase inhibitor that binds to the kinase DFG-out inactive form. Figure 65 illustrates the binding interactions of compound **59** with the DFG-out form of p38α. Pyrazole carboxamide *NH* acts as a hydrogen bond donor with the Thr gatekeeper, the pyrazole ring nitrogen interacts with the hinge region, and the *N*-phenyl forms a hydrophobic interaction with the P-loop tyrosine. The other part of the structure containing cyclohexyl and bisamide occupies a type-II pocket. When tested at 2 µM concentration against a 99-human kinase panel, it showed preferential selectivity towards p38α and p38β compared to the other 97 tested kinases. In a human monocytic cell line, compound **59** strongly inhibited TNF-α secretion with an IC_50_ value of 46 nM. Replacement of the terminal primary amide with the *N*-ethyl-*N*-methyl tertiary amide led to a selective type-II inhibitor of p38α despite weaker potency (IC_50_ = 14 nM). It was tested against a 468-kinase panel at 1 µM concentration and inhibited only thirteen kinases including p38α and p38β (98.5% and 96.6% inhibition, respectively) [71].

### 20.4. Compound ***60***

Compound **60** (Figure 63) is the most potent inhibitor and antiproliferative agent among a series of 1,3,4-triarylpyrazole derivatives. Upon testing against a 15-kinase panel, it inhibited V600E-B-RAF, C-RAF, FLT3, and P38α/MAPK14. The highest potency was reported against p38α with an IC_50_ of 515 nM. In addition, it exerted potential antiproliferative activity against National Cancer Institute’s (NCI) 60 cancer cell line panel, and the three most sensitive cell lines are the RPMI-8226 and K-562 leukemia cell lines in addition to the MDA-MB-468 breast cancer cell line (IC_50_ values equals 1.71, 3.42, and 6.70 µM, respectively). Compound **60** induces apoptosis but not necrosis in the RPMI-8226 leukemia cell line. Replacement of the methoxy group with hydroxyl or any substitution on the pyridyl ring reduced the activity [73].

## 21. Pyrazole-Based PDK Inhibitors

### Compound ***61***

**Figure 66:**
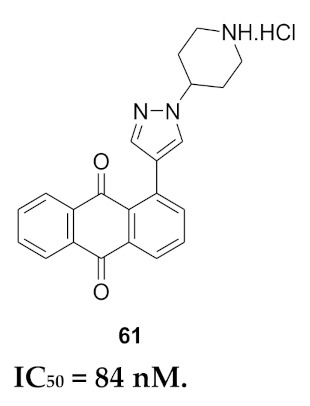
Structure of compound **61**, a PDK4 inhibitor.

Pyruvate dehydrogenase kinase 4 (PDK4) inhibition is a potential avenue for treatment of metabolic disorders such as hyperglycemia and insulin resistance, in addition to cancer and allergies. Ahn et al., have reported a series of pyrazole-possessing anthraquinone derivatives as inhibitors of the PDK4 kinase. Compound **61** (Figure 66) is the most potent PDK4 inhibitor among this series (IC_50_ = 84 nM). Any substitution on the piperidine *NH* or any modification of the ring carbonyl led to diminished activity. Compound **61** could enhance the glucose tolerance in the diet-induced obesity model in mice. In addition, it alleviated the allergic reactions in a passive cutaneous anaphylaxis model in mice. Moreover, it exerted modest antiproliferative activity against some cancer cell lines with 2-digit micromolar IC_50_ values. Compound **61** also demonstrated a weak inhibitory effect against CYP isozymes with 2-digit micromolar range. After oral administration of 10 mg/kg dose of compound **61** to male rats, the oral bioavailability was 63.6%, and t1/2 and t_max_ were 21.6 and 6 h, respectively [74].

**Figure 67:**
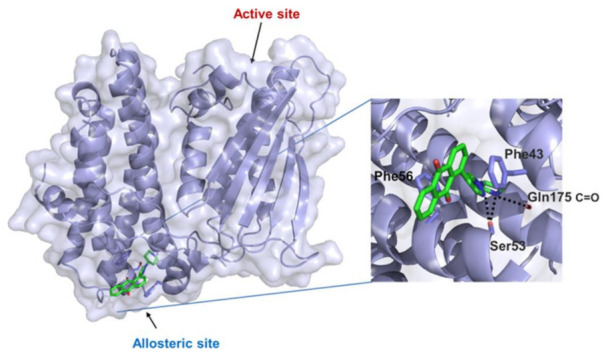
Putative binding mode of compound **61** with the lipoamide allosteric site of PDK4 kinase crystal structure [74].

The docking study indicated that compound **61** binds to the allosteric lipoamide site, not the active site. The pyrazole and piperidine rings entered the pocket, while the anthraquinone motif remained at the gate, intercalated between Phe43 and Phe56 on the surface and formed hydrophobic interactions with them. Furthermore, the pyrazole nitrogen atoms formed hydrogen bonds with the Ser53 hydroxyl group and the backbone carbonyl oxygen atom of Gln175 (Figure 67) [74].

## 22. Pyrazole-Based Pim Kinase Inhibitors

### 22.1. Compound ***62***

**Figure 68:**
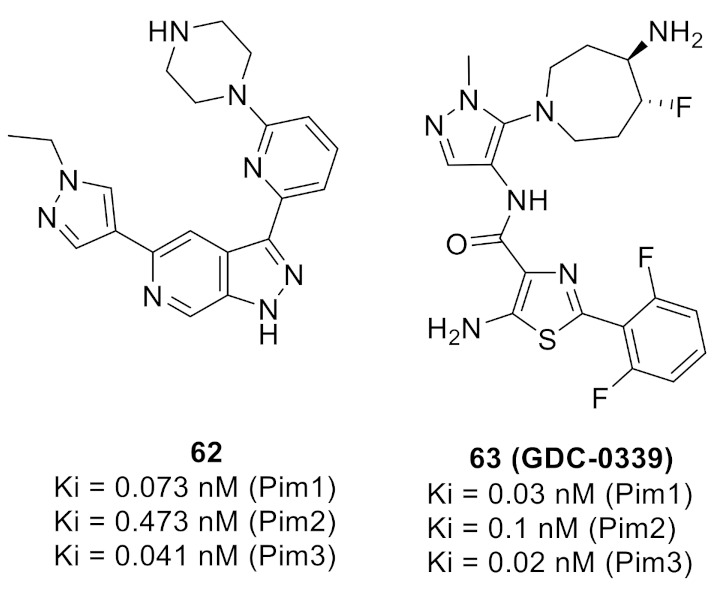
Structures of pyrazole-based Pim kinase inhibitors and their Ki values.

Pim kinases are good targets for management of different disorders including multiple myeloma. Compound **62** (Figure 68) has been reported as a pan-Pim kinase inhibitor. In addition, it demonstrated antiproliferative activity against the MM1.s myeloma cell line (IC_50_ = 0.64 µM). The 6-azaindazole core scaffold is more favorable for activity than indazole. The *N*-ethylpyrazole is more optimal than other substituted pyrazole rings, and piperazine is better than other alicyclic rings. However, compound **62** suffers from low oral bioavailability (1%) and high plasma protein binding (97.8%). The poor oral bioavailability could be attributed to low permeability. Further structural optimization should be carried out in order to improve the PK profile [75].

### 22.2. Compound ***63***

Compound **63** (GDC-0339, Figure 68) has been developed as an orally bioavailable pan-Pim kinase inhibitor with antiproliferative activity against multiple myeloma. It was further tested against a panel of 277 kinases at 100 nM concentration, which is 500–5000 times its K_i_ values against Pim kinases, and showed more than 50% binding to only twelve kinases. In addition, it exerted a high potency against the MM1.s myeloma cell line with an IC_50_ value of 0.07 µM. It was also tested in vivo against MM1.s and RPMI 8226 mice models of multiple myeloma and exhibited promising results. The 2,6-difluorophenyl moiety is more optimal for activity than monofluorophenyl. In addition, the presence of thiazole and pyrazole rings together in the structure is the best combination compared to the 5-membered/6-membered or two 6-membered rings [76].

**Figure 69:**
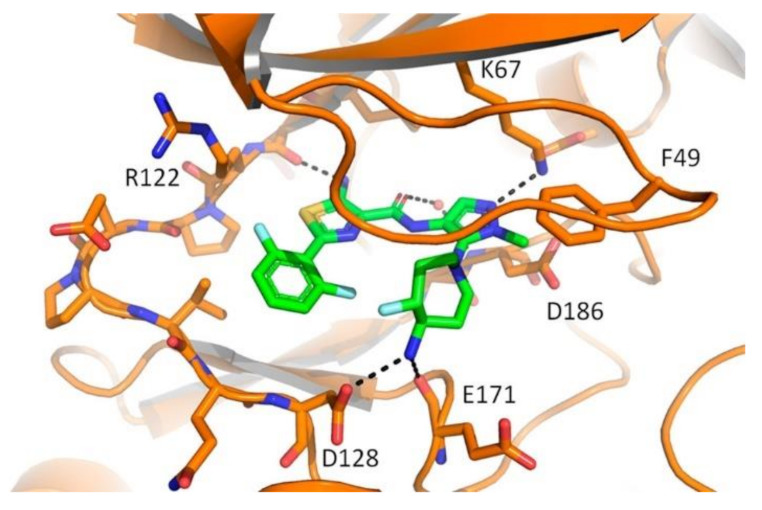
In silico binding interactions of compound **63** with Pim1 crystal structure [76].

The docking of compound **63** into the Pim1 crystal structure revealed its fitting into the ATP-binding site. The primary amino group attached to the 7-membered ring forms a salt bridge with Asp128 and Glu171. The other primary amine donates a hydrogen bond to Arg122. Furthermore, the non-methylated pyrazole nitrogen atom accepts a hydrogen bond from Lys67 terminal amine (Figure 69) [76].

## 23. Pyrazole-Based RAF Kinase Inhibitors

### 23.1. Compound ***64***

**Figure 70:**
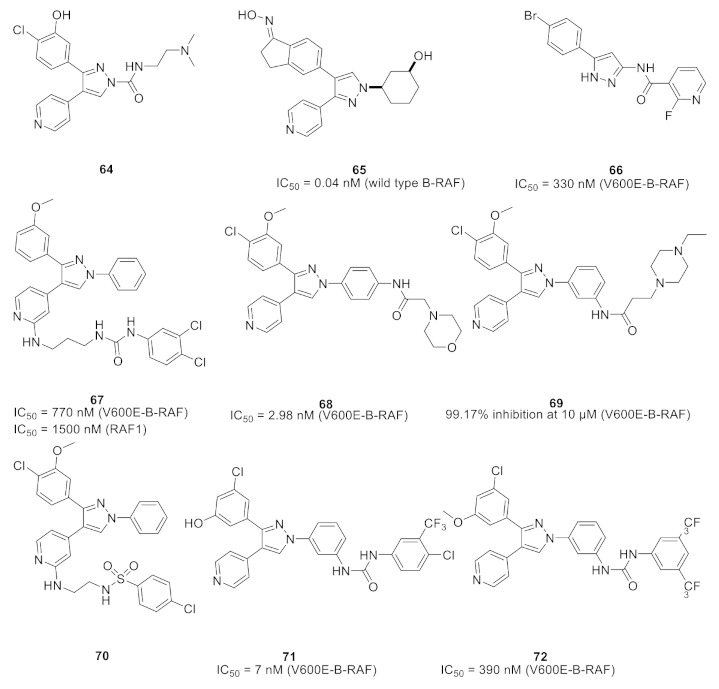
Structures of pyrazole-based RAF kinase inhibitors and their IC_50_/Ki values or percentage inhibition.

**Figure 71:**
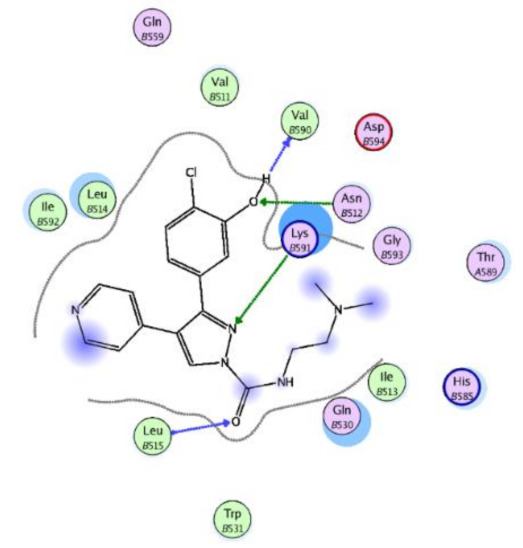
Putative binding interactions of compound **64** with V600E-B-RAF crystal structure [77].

Compound **64** (Figure 70) is the most potent among a series of 3,4-diarylpyrazole-1-carboxamide derivatives reported as antiproliferative agents against the A375P human melanoma cell line in which mutant V600E-B-RAF kinase is over-expressed. Its IC_50_ value against A375P cell line equals 4.5 µM. The docking of compound **64** into the domain of V600E-B-RAF was carried out to investigate its putative binding mode (Figure 71). The phenolic hydroxyl group forms two hydrogen bonds with Val B590 and Asn B512, pyrazole *N2* forms a hydrogen bond as acceptor with the Lys B591 amino acid residue, and the urea carbonyl oxygen accepts a hydrogen bond from Leu B515. The structure-activity relationship (SAR) study showed that terminal dimethylamino is more optimal for activity than bulkier dialkylamino or cyclic amines. In addition, the phenolic group is more favorable than methoxy, and this was supported by the docking study demonstrating the contribution of OH as a hydrogen bond donor. In addition, compound **64** obeys Lipinski’s rule of five so it is estimated to be orally bioavailable [77].

### 23.2. Compound ***65***

Compound **65** (Figure 70) is another example of vicinal diarylpyrazole derivatives reported as RAF kinase inhibitors. It showed various B-RAF kinase inhibitory effects when different hydroxylated cycloalkyl groups were placed at the *N1* position of the pyrazole ring. Docking, molecular dynamics (MD) simulations, and hybrid calculation methods (Quantum Mechanics/Molecular Mechanics (QM/MM)) were conducted on the complexes to explain these differences. Compound **65** is the most potent against B-RAF kinase with an IC_50_ value of 0.04 nM. Compound **65** forms hydrogen bonding interactions with Glu501 and Cys532 and is surrounded by Val471, Lys483, Thr529, Leu514, Asp594, Phe583, Ala481, and Trp531. The trans isomer (with the hydroxyl group behind the plane) is less potent against the kinase (IC_50_ = 0.09 nM). Replacement of the cyclohexyl ring with cyclopentyl led to decreased potency [78].

### 23.3. Compound ***66***

**Figure 72:**
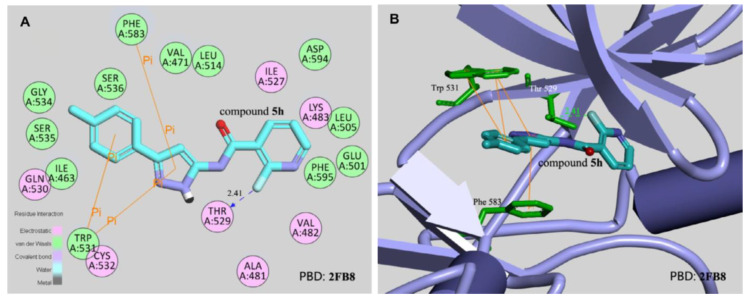
Putative binding interactions of compound **66** with V600E-B-RAF crystal structure [79]. (**A**) 2D interactions; (**B**) 3D interactions.

A series of novel 5-phenyl-1*H*-pyrazole analogues possessing the niacinamide motif have been reported as potential V600E-B-RAF inhibitors. Compound **66** (Figure 70) exhibited the strongest potency against V600E-B-RAF kinase (IC_50_ = 330 nM). Compound **66** also demonstrated the best antiproliferative potency against WM266.4 and A375 melanoma cell lines with IC_50_ values of 2.63 and 3.16 µM, respectively, which are comparable with vemurafenib. Strong electron-withdrawing substituents such as fluoro on the pyridyl ring are beneficial for the activity. The putative binding mode of compound **66** is shown in Figure 72. It neatly binds to V600E-B-RAF via one hydrogen bond with a fluoro substituent and three pi–pi interactions through the pyrazole and phenyl rings [79].

### 23.4. Compound ***67***

A novel series of 1,3,4-triarylpyrazole derivatives containing terminal arylamide or arylurea moieties have been reported as RAF kinase-inhibiting antiproliferative agents. Compound **67** (Figure 70) showed the best mean inhibition percentages values over the National Cancer Institute’s (NCI) 58 cell line panel at 10 µM. Compound **67** was tested against seven kinases at 10 µM concentration to profile its kinase inhibitory activities. Compound **67** showed high inhibitory effects (90.44% and 87.71%) against the V600E-B-RAF and RAF1 kinases, respectively. Its IC_50_ values over both kinases are 0.77 and 1.50 µM, respectively. Compound **67** possessing the 3′,4′-dichlorophenylurea terminal moiety showed the most promising results at 5-dose testing. A urea spacer is more favorable for activity in this series than an amide. It exhibited promising potency, efficacy, and broad-spectrum antiproliferative activity against many cancer cell lines of different cancer types (one-digit micromolar IC_50_ values), as well as being superior to sorafenib [80].

### 23.5. Compound ***68***

**Figure 73:**
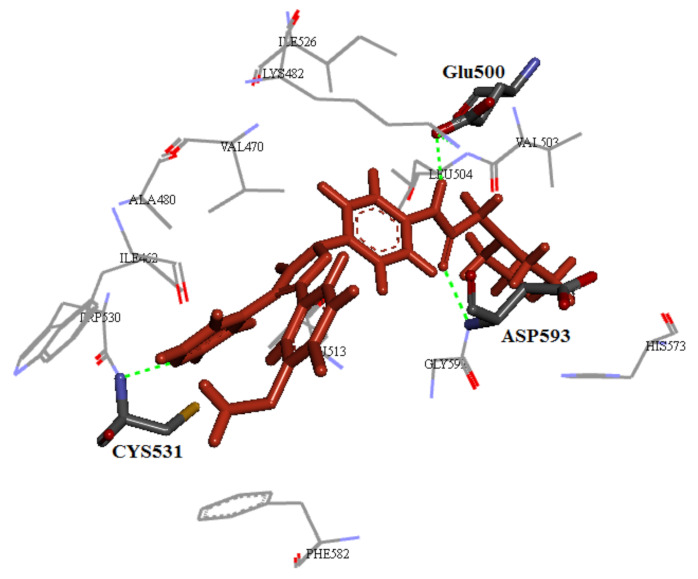
In silico binding interactions of compound **68** with V600E-B-RAF crystal structure [81].

A series of 1,3,4-triarylpyrazoles with an amide spacer were reported as RAF kinase-inhibiting anti-melanoma agents. Among them, compound **68** (Figure 70) is the most potent against A375 melanoma cells (IC_50_ = 1.82 µM), with a selectivity index of 45.83 toward A375 rather than the HS27 normal fibroblasts. Compound **68** showed higher potency against the melanoma cell lines that include B-RAF V600E mutation compared to melanoma cells possessing the NRAS mutation as well as normal epithelial skin cells. Compound **68** is highly potent and selective against the V600E-B-RAF kinase with an IC_50_ value = 2.98 nM. It has one carbon linker between the amide group and the morpholino nitrogen atom in its structure, and it is more active and more potent than the corresponding analogue with no linker. Compound **68** is about two-fold more potent than sorafenib and GW5074 against V600E-B-RAF. The molecular docking study revealed that compound **68** belongs to Type-II kinase inhibitors that bind to the kinase inactive form. Its amide oxygen atom showed an additional hydrogen bond formation with the kinase crystal structures in the docking study that enhances the affinity and potency. In addition, the pyridyl nitrogen forms a hydrogen bonding interaction with the Cys531 amino acid residue (Figure 73) [81].

### 23.6. Compound ***69***

As an extended study, a series of positional isomers of compound **68** (Figure 70) possessing a *meta*-disubstituted benzene ring at *N1* of the pyrazole ring was reported. These positional isomers are generally more active than the *para*-analogues. Among them, compound **69** (Figure 70) possessing an ethylene spacer between the amide group and the piperazinyl nitrogen is the most promising. It was examined on the NCI-60 cancer cell line panel and showed a 97.72% mean inhibition percentage at 10 µM concentration. Its IC_50_ values are within sub-micromolar range (0.27–0.92 µM) against nine cancer cell lines of nine cancer types. Against the A375 melanoma cell line, its IC_50_ value is 0.82 µM, which is 2.22 times more potent than compound **68**. Furthermore, compound **69** inhibits 99.17% of the V600E-B-RAF kinase activity at 10 µM concentration. The SAR study of this series revealed that *N*-methylpiperazinyl is more optimal for activity than the higher alkyl-substituted piperazinyl. In both the series of compounds **68** and **69**, the methoxy group is more favorable than hydroxyl [82].

### 23.7. Compound ***70***

Compound **70** possessing *p*-chlorobenzenesulfonamido at a terminal position, an ethylene linker, and a 4-chloro-3-methoxyphenyl ring at the C-3 position of the pyrazole core ring is the most promising anticancer agent among its series of compounds (Figure 70). It showed the highest mean percentage inhibition value (66.71%) against the NCI-60 cancer cell line panel at 10 µM concentration. It exerted broad-spectrum activity against various cell lines of different cancer types. Moreover, compound **70** exerted a higher range of selectivity against the HT29 colon cancer cell line than the HL-60 leukemia and MRC-5 lung fibroblasts (normal cells). Upon testing against 12 kinases of different kinase families, compound **70** gave a higher inhibitory effect over three RAF kinases. It produced 78.04%, 74.47%, and 72.46% inhibition at 10 µM concentration against the RAF1, V600E-B-RAF, and V600K-B-RAF kinases, respectively. The SAR study showed that an ethylene linker is more optimum for activity than propylene [83].

### 23.8. Compounds ***71*** and ***72***

**Figure 74:**
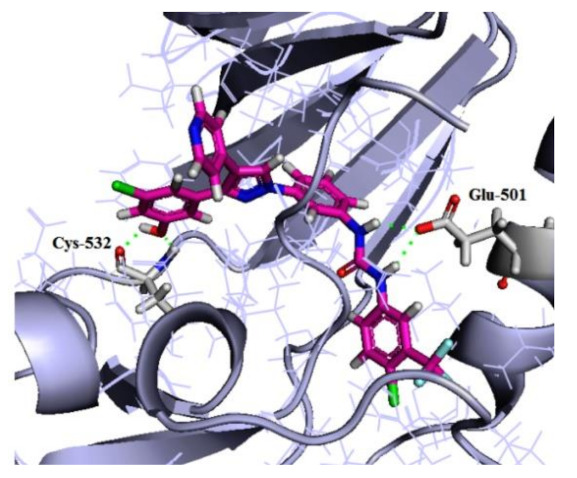
In silico binding interactions of compound **71** with V600E-B-RAF crystal structure [84].

**Figure 75:**
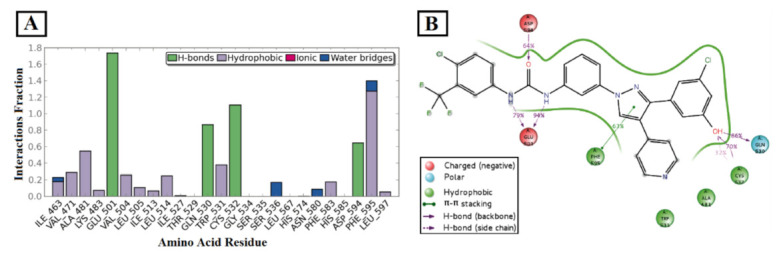
V600E-B-RAF-compound **71** binding interactions throughout the 50 ns simulation period; (**A**) the fractions of interaction happened between compound **71** and V600E-B-RAF kinase. (**B**) 2D interaction diagram of compound **71** within V600E-B-RAF active site [84].

Compound **71** (Figure 70) is the most potent RAF kinase-inhibitory derivative among a series of pyrazole-containing diarylureas. Its IC_50_ value against the V600E-B-RAF kinase equals 7 nM. Docking and molecular dynamic simulation studies revealed that compound **71** is a type IIA inhibitor of V600E-B-RAF. The SAR revealed that the phenolic OH on the aryl ring attached to the C-3 of pyrazole is more optimal for activity than OMe. OH forms a bidentate hydrogen bonding with the Cys532 amino acid residue. In addition, a urea linker is more favorable than amide as the two *NH* groups of urea form hydrogen bonds as donors with the αC-helix Glu501amino acid residue (Figure 74). In addition, the pyrazole ring forms a hydrophobic interaction with the Phe595 residue, and the urea oxygen interacts with Asp594 residue (Figure 75) [84]. Moreover, compound **71** was tested for anticancer activity over the NCI-60 cancer cell lines of nine cancer types and showed promising activity. Its mean inhibition percentage over the sixty cell lines is more than 89.54%. In addition, its mean IC_50_ value against the nine subpanels was within the range of 1.98–3.26 µM. Hydrophobic and electron-withdrawing substituents (e.g., Cl & CF_3_) on the terminal aryl ring attached to urea spacer are favorable for activity. Furthermore, compound **72** is another promising antiproliferative agent despite its lower potency against the V600E-B-RAF kinase (IC_50_ = 390 nM). It was tested against 58 cancer cell lines of nine different cancer types at the NCI and exerted higher potency than sorafenib against all the 58 tested cell lines. Its IC_50_ values are within the submicromolar range against most of the tested cell lines. It exerted weak potency against the RAW 264.7 non-cancerous cell line, and induced apoptosis in the RPMI-8226 leukemia cell line with an IC_50_ of 1.52 µM [85].

## 24. Pyrazole-Based ROS Kinase Inhibitors

### Compound ***73***

**Figure 76:**
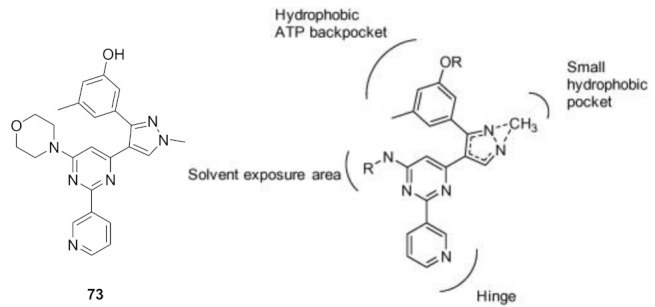
Structure of compound **73** and a simplified top view of a general scheme depicting the binding mode of trisubstituted prazoles in ROS1 and key interactions involved [86].

The ROS1 is a tyrosine kinase whose function is not fully understood, but its role in the development of resistance in some cancers made it a valuable target for management of some cancers. A series of trisubstituted pyrazoles were designed, synthesized and biologically evaluated. The group’s previous work on the same scaffold had advanced an understanding of the structure-activity relationship of this scaffold and was summarized in Figure 76 [86].

The key interactions that constitute the SAR of trisubstituted pyrazoles consist of the distal unsubstituted pyridinyl ring, which interacts with the hinge region’s Met2029 and loses its activity upon replacing it with a phenyl ring, or there is a deterioration in activity upon the introduction of substituents to the pyridinyl ring due to a steric clash which prevents bonding. The pyrazle’s methyl binds to a small hydrophobic pocket, which does not tolerate polar groups, or bigger substituents. Both positional isomers of pyrazole appear to be biologically active with a preference to 1-methylpyrazole over the other isomer. The pyrazole’s role is to direct the substituent and the arms into optimal positions to interact with the ROS1 binding site [86].

The series was tested against the ROS1 enzyme. Compound **73** (Figure 76) displayed the highest potency among the series (IC_50_ = 13.6 nM) compared to crizotenib (IC_50_ = 60 nM). Compound **73′**s activity was a strong motivation to investigate the selectivity on a panel of kinases. Using the KINOMEscan™ screening platform, compound **73** was tested on 456 non-mutant and disease related mutant kinases. ROS1, FLT3, JAK2, and TYK2(JH1 domain-catalytic) were shown to be inhibited by **73** at 10 μM, while it did not inhibit the ALK and c-MET enzymes which are the most homologous to the ROS1 kinase. Selectivity was quantitatively measured using the selectivity score parameter, which is obtained by dividing the number of kinases that compounds bind to by the total number of distinct kinases tested, excluding mutant variants. Compound **73** had a selectivity score of 0.076 compared to Imitanib (0.12) and Dasatinib (0.26) [86].

**Figure 77:**
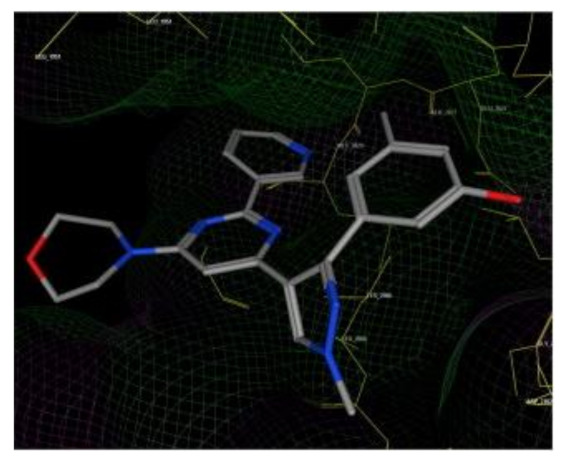
The binding motif of **73** in ROS1 kinase [86].

The docking of **73** into the binding site of unphosphorylated ROS1 was performed (Figure 77). The binding mode obtained further strengthened the hypothesis of the proposed SAR generated by the group’s work. Pyridinyl bonded with Met2029 of the hinge region via H-bonding, the disubstituted phenyl moiety was directed to a certain hydrophobic pocket under the P-loop, and Morpholine reached out into the solvent [86].

## 25. Pyrazole-Based Src Kinase Inhibitor

### Compound ***74***

**Figure 78:**
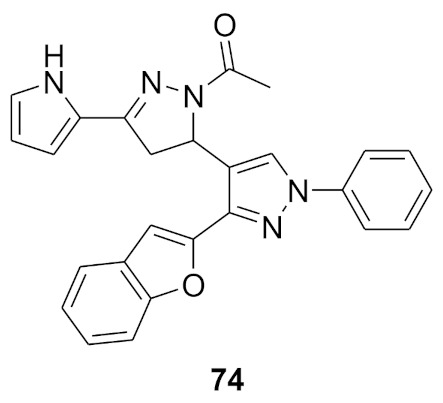
Structure of compound **74**, a Src kinase inhibitor.

**Figure 79:**
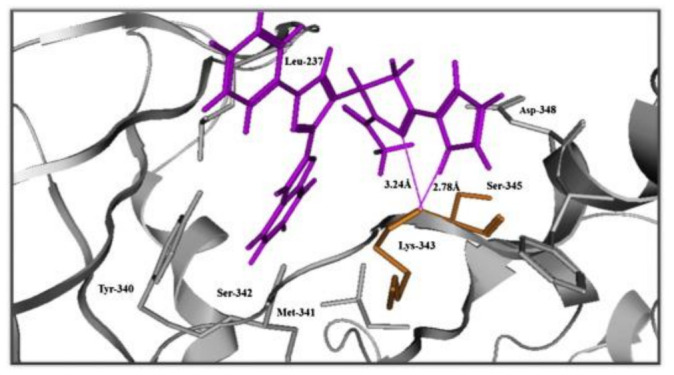
In silico binding interactions of compound **74** with Src crystal structure [87].

Compound **74** (Figure 78) was reported as a broad spectrum antiproliferative agent possessing an inhibitory effect against the Src kinase. Upon testing against the NCI-60 cancer cell line panel, it exerted one-digit micromolar IC_50_ values against several cell lines of a variety of cancer types. Its highest potency was against the CCRF-CEM and MOLT-4 leukemia cell lines (IC_50_ = 1.00 µM against both of them). The SAR shows that replacement of the acetyl group with hydrogen diminished the activity. Concerning the pyrazoline ring, expansion or replacement with chalcone decreased the activity while replacement with an isoxazole ring retained it. Compound **74** was further tested against eight kinases and the highest activity was reported against Src (59% inhibition at 10 µM concentration). In silico studies showed that compound **74** obeys Lipinski’s rule of five and has an acceptable PK profile. In addition, docking studies demonstrated that compound **74** occupies the ATP-binding pocket of Src. The pyrrole *NH* forms a hydrogen bond with Lys343. Compound **74′**s pyrazoline nitrogen accepts a hydrogen bond from Ser345. Moreover, the benzofuran benzene ring forms hydrophobic interactions with Leu273 and Gly274 (Figure 79) [87].

## 26. Pyrazole-Based TGFβ/ALK Kinase Inhibitors

### 26.1. Compounds ***75*** and ***76***

**Figure 80:**
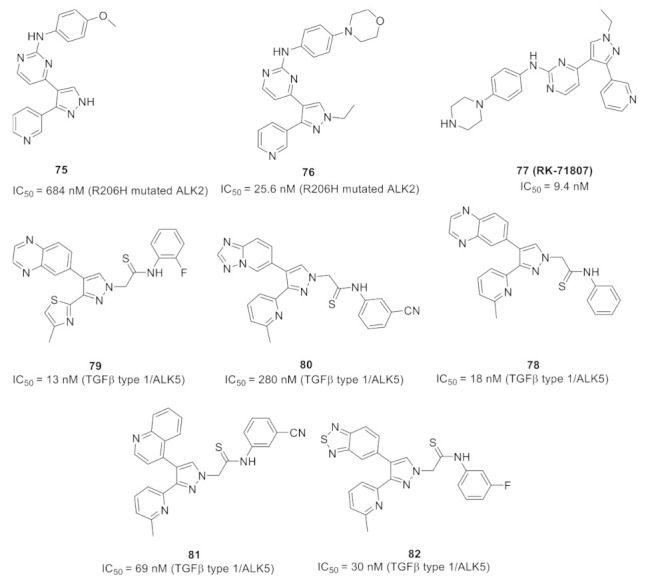
Structures of pyrazole-based TGFβ/ALK inhibitors and their IC_50_ values.

**Figure 81:**
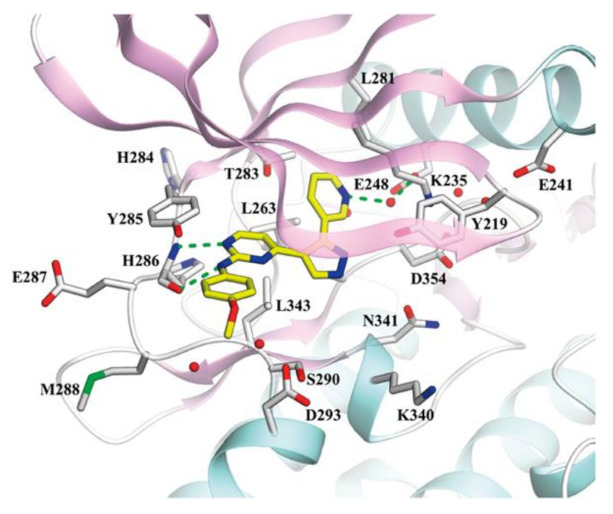
X-ray crystal structure of lead compound **75** in R206H mutant ALK2. White stick model: residues forming ATP binding pockets; Yellow stick model: lead compound; Green dashed lines: the hydrogen bonds between lead compound and active site; Red sphere: water molecules [88].

The motive for the development of compound **76** (Figure 80) was the lack of active compounds with desirable physiochemical properties and oral activity against the R206H mutated ALK2. Through in silico and in vitro investigations, a lead compound **75** (Figure 80) consisting of the vicinal diarylpyrazole compound was identified. The cocrystal structure of the lead with the R206H mutated ALK2 showed the interactions between the aminopyrimidine ring and the hinge region, the hydrogen bond via the water molecule between 3‘-pyridyl’s nitrogen, and the carboxylate of the Glu248 side chain from the αC helix while the pyrazole ring was embedded inside the sugar pocket and anisidine’s methoxy was projected into the solvent region (Figure 81) [88].

Using the data from the cocrystal structure, the same research group hypothesized that introduction of polar groups on anisidine and pyrazole moieties would improve activity and physiochemical properties such as aqueous solubility and liver microsomal stability. They started with ring replacement of the 3‘-pyridyl ring with different heterocycles, which proved detrimental for activity. Introduction of different chains on the two nitrogens of the pyrazole ring showed that an unsubstituted pyrazole lead compound and an alkyl substituent on *N1* were inferior to an *N2* substituent in terms of inhibitory activity and cell permeability, and *N2*- ethylpyrazole is the best substituent. The group decided to move on to the next step: investigation of the role of substituents on anisidine moiety. It was concluded that compound **76** with *p*-morpholine anisidine had the best activity among the synthesized compounds. Compound **76** had an IC_50_ of 25.6 nM. Permeability was measured using Caco-2 cells which exhibited A to B Papp= 9.12 × 10^−6^ cm/s. Efflux ratio of compound **76** was 1.0 (calculated by taking the ratio of A to B compared to B to A). Moreover, the multidrug resistance (MDR) was determined using Madin–Darby canine kidney (MDCK) cells which had a ratio of 1.6. Moreover, compound **76** had an aqueous solubility of 200.1 µM and human plasma protein binding of 91.8%. The in vivo study of the pharmacokinetic properties of compound **76** on rate showed good oral bioavailability (F = 56%) [88].

### 26.2. Compounds ***75*** and ***77***

**Figure 82:**
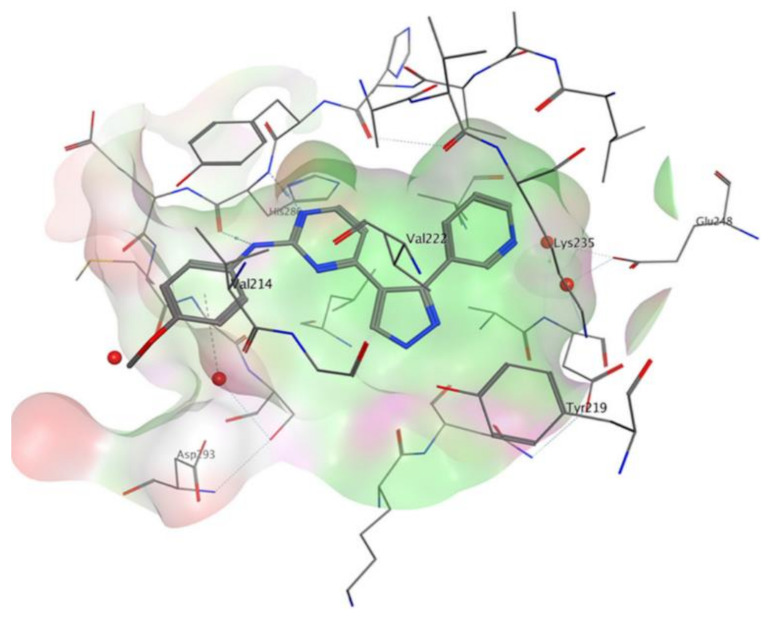
In silico binding interactions of RK-59638 (**75**) with ALK2 (R206H) crystal structure [89].

Compounds **75** (RK-59638) and **77** (RK-71807) were reported as ALK2 (R206H) kinase-inhibiting lead compounds for treatment of fibrodysplasia ossificans progressive (Figure 80). The IC_50_ value of compound **75** is 684 nM against the ALK2 (R206H) kinase. The docking study demonstrated hydrogen bonding between the amino pyrimidine moiety and the Ser286 nitrogen in the hinge region. The pyridine ring forms water-mediated hydrogen bonding with Glu248. In addition, its pyrazole ring forms a hydrophobic interaction with Val222 (Figure 82) [89].

The *N1* of the pyrazole ring in compound **75** does not contribute to the binding interactions with the kinase. Different substituents on it were investigated, and ethyl was the optimum substituent. The methoxy group was also replaced with a piperazinyl ring to obtain compound **77**. These structural modifications led to improved potency and physicochemical properties. The IC_50_ of compound **77** against the kinase is 9.4 nM, which is about 72.8-fold more potent than compound **75**. The aqueous solubility of compound **77** at pH 7.4 has improved to reach 93.8 µg/mL (vs. 6.4 µg/mL in case of compound **75**). Plasma protein binding decreased from 85% for compound **75** to 65.8% for compound **77**. Compound **77** also showed less inhibitory effects than compound **75** against the cytochrome P450 isozymes. Furthermore, compound **77** exerted only 27% inhibition of hERG upon testing at 10 µM [89].

**Figure 83:**
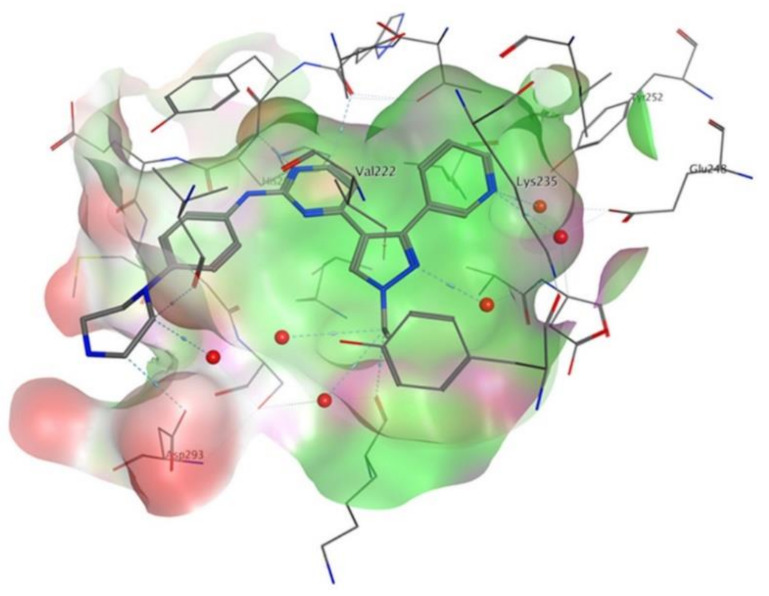
In silico binding interactions of RK-71807 (**77**) with ALK2 (R206H) crystal structure [89].

The putative binding interactions of compound **77** with the kinase crystal structure indicate electrostatic interaction of the protonated piperazine nitrogen with Asp293. The ethyl group exerts a hydrophobic interaction with Tyr219 phenyl ring. Moreover, the pyrazole *N1* forms hydrogen bonding with Lys235 (Figure 83) [89].

### 26.3. Compound ***78***

**Figure 84:**
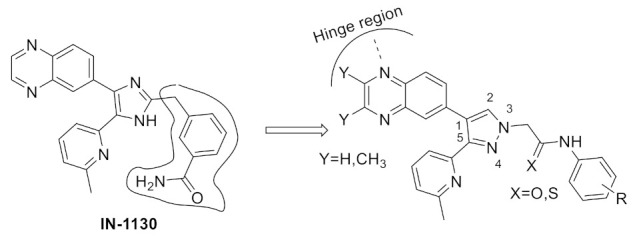
A schematic view of the rationale behind the development, as well as a hypothetical H-bonding between quinazoline’s N and hinge region [90].

The development of TGFβ type 1/ALK5 inhibitors based on their clinical candidate IN-1130 as a lead compound has been reported. That group’s previous works had concluded that activity and selectivity were higher in the presence of methyleneamide and methylenethioamide linkers against the ALK5 kinase. They also concluded that although thiazide linkers might be superior in activity in comparison to methylenethioamide, but owing to shelf stability issues during long term storage, the methylenethioamide and methyleneamide are better options for development. To study the tolerability of the ALK5 active domain to the length of the tether between the phenyl ring and central pyrazole ring, the group synthesized few ethyleneamide linkers to be investigated among the synthesized compounds. Another point which was considered during the investigation was the introduction of 2,3-dimethyl substituents on the quinazoline ring, which is thought to improve the H-bonding between the *N* of the quinazoline ring and the hinge region of ALK5 (Figure 84) [90].

A total of sixteen compounds were synthesized and biologically evaluated. A kinase assay using purified human ALK5 kinase was produced in Sf9 insect cells to evaluate ALK5 inhibition. All compounds with a 2,3-dimethylquinazoline moiety did not show any activity up to concentration of 1 µM. In addition, the 3-substituted pyrazole ring had superior activity compared to its positional isomer. The ethyleneamide linker experienced a loss of activity compared to methyleneamide, and methylenethioamide derivatives were superior to methyleneamide. The most potent compound against ALK5 was compound **78** (Figure 80). Further investigation through measuring the luciferase activity in a cell-based assay to determine the TGF-β-induced downstream transcriptional activation to ALK5 signaling was performed. Using HaCaT cells permanently transfected with a p3TP-luciferase reported construct, the results did not differ much from the kinase assay showing a similar trend. Compound **78** inhibited luciferase activity by 80% at 0.1 μM. Compound **78** was further tested and compared to IN-1130 and SB-505154 (a known ALK5 inhibitor) at five different concentrations. Compound **78** inhibited ALK5 in a dose-dependent fashion, it was equipotent with IN-1130, and more potent than SB-505154. The p38α MAP kinase’s active site is one of the most homologous to that of the ALK5’s, which was chosen to investigate the selectivity of the compounds in this series. The series is devoid of activity towards the p38α MAPK up to a concentration of 1 μM, with compound **78** being the most selective towards ALK5 compared to p38α MAPK (selectivity index > 77) [90].

**Figure 85:**
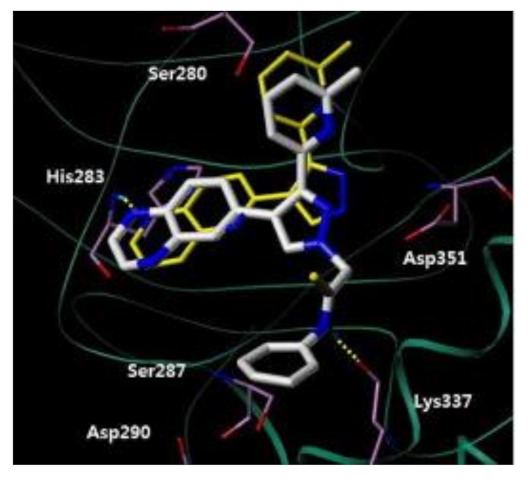
Binding pose of compound **78** in the active site of ALK5, superimposed over the X-ray pose of 1,5-naphthyrine inhibitor (yellow carbon). Yellow dotted lines indicate hydrogen bonding interactions (<2.5 Å) [90].

The docking of compound **78** to study its binding mode and interactions with the active site of ALK5 is shown in (Figure 85). A quinoxalinyl ring mimics the adenine ring of the ATP’s pocket and formed H-bonding with the backbone of His283, and the thioamide’s NH formed H-bonding with Lys337 in the catalytic domain of ALK5 [90].

### 26.4. Compound ***79***

**Figure 86:**
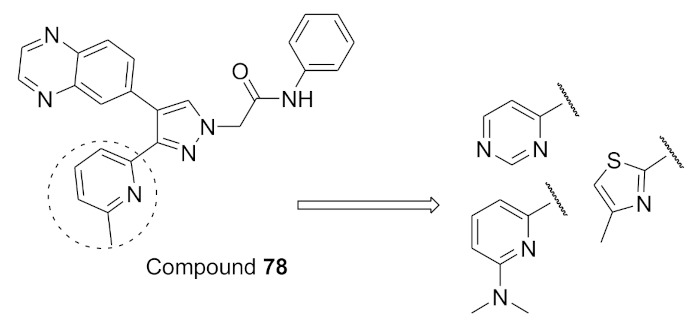
Schematic view showing the design strategy used to modify the *o*-methylpyridinyl motif.

Continuing to build on compound **78**, the group went on to modify the *o*-methylpyridinyl motif (Figure 80). The methylpyridinyl motif’s importance lies in its ability to form a hydrophobic interaction with Tyr249 of the ALK5’s active site, the nitrogen of pyridine forms a water-bridged H-bonding with the side chains of Tyr249 and Glu245 and the backbone of Asp351. Keeping in mind the hypothesis that the inhibitory activity could be improved if the H-bonding of the pyridine motif was stronger, the group sought to replace the methyl group with dimethylamine to study the effect of an electron donating group on improving the H-bonding of the *N* of the pyridine. They also sought bioisosteric replacement of the pyridine ring with the 4-methylthiazol-2-yl and 4-pyrimidinyl groups to study its effect on activity (Figure 86) [91].

A series compromising of thirty-two compounds was synthesized. The series was tested on a purified human ALK5 kinase domain produced in Sf9 insect cells at 10 μM to evaluate ALK5 activity. The series followed the previously discussed trend in compound **78**, methylenethioamide was superior to methyleneamide, and the 3-substituted pyrazole was superior to its positional isomers. The 6-Methylpyridine was superior to its 6-dimethylaminopyridine counterpart. It is possible that fitting into the enzyme’s active site was not achieved due to it being bulkier compared to the methyl group. Nonetheless, the series containing 4-methylthiazol-2-yl showed highly improved activity compared to 6-methylpyridine, with compound **79** (Figure 80) showing the highest inhibition (2% residual activity upon testing at 10 µM concentration) and an IC_50_ of 0.28 μM. Compound **79** was further tested on p38α MAPK at 10 μM since its kinase domain is one of the most homologous to the ALK5’s kinase domain, and lacked any inhibitory activity against it. The most potent of all, compound **79** had a selectivity index of >35 against ALK5 compared to p38α MAPK [91].

**Figure 87:**
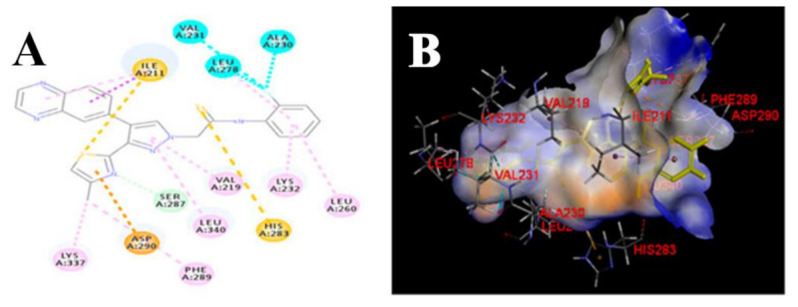
Docking pose of compound **79** in the active site of ALK5. (**A**) 2D binding interactions pro; (**B**) Proposed pose of **79** in the binding pocket of ALK5. The ligands are shown in yellow [91].

Compound **79** and its 6-dimethylaminopyridnyl counterpart were docked into ALK5’s active site (Figure 87). The docking study explained the higher activity of 4-methylthiazol-2-yl of compound **79** compared to its 6-dimethylaminopyridinyl counterpart with its higher number of bondings, five bonds, in comparison to the two bonds of its dimethylaminopyridinyl counterpart. The phenyl ring of compound **79** bonds with Lys232 via a pi-alkyl bond. The pyrazole ring interacts with the side chains of Leu340 and Val219. Thiazole’s contribution to activity was notable as well. The ring’s nitrogen bonds with the backbone of Ser287 through a carbon–hydrogen bond. Methyl’s involvement with the backbone of Lys337 is through an alkyl bond, as well as with the backbone of Phe289 through a pi-alkyl bond [91].

### 26.5. Compound ***80***

**Figure 88:**
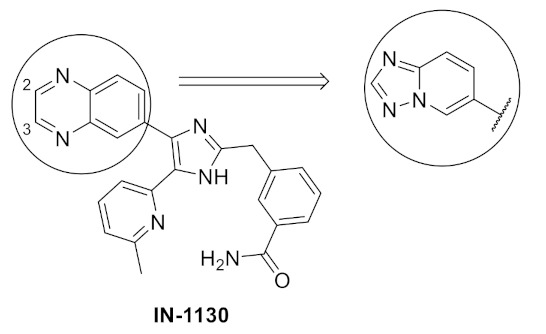
Preclinical candidate IN-1130 and possible oxidation site at position 2 or 3 on the quinazoline’s heterocycle.

The motivation for carrying out this work stems from the desire to overcome the metabolic oxidation of preclinical candidates IN-1130 (Figure 88). The extensive history of studying and developing a potent and selective ALK5 (Activin receptor-Like Kinase 5) inhibitor by this group had led them to draw a pharmacophore that can effectively and selectively inhibit ALK5. A central five-membered ring with a small tether (e.g., methylene, methylenethioamido and methyleneamide) attached to a substituted phenyl ring (fluorine atom at position 2 or carbonitrile or carboxamido groups at position 3 and 4) and a 2-pyridyl heterocycle attached to the central 5-membered ring can constitute a framework as an ALK5 inhibitor and hinge region binder like the quinazoline ring [92].

**Figure 89:**
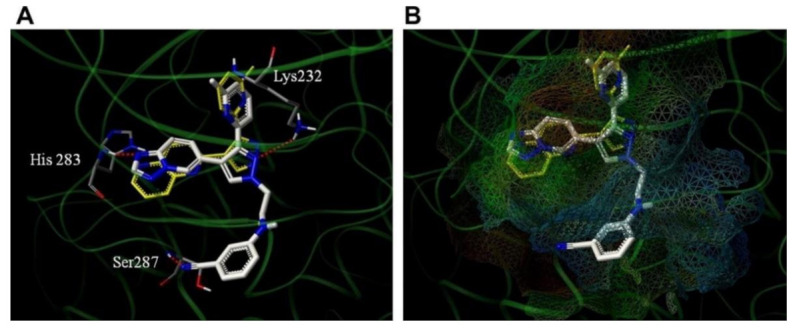
Putative binding mode of compound **80** with ALK5. (**A**) White, **80′**s binding mode in the active site; Yellow, 1,5-napthyrine; Grey, key amino acid residues represented in line form; Red, hydrogen bond interaction (<2.8 Å); compound **80** is superimposed over 1,5-naphthyrine inhibitor. (**B**) MOLCAD49 lipophilic potential surface map of ALK5’s active site in the docking model of **80**. Lipophilicity increases from blue (hydrophilic) to brown (hydrophobic) [92].

It was hypothesized that the bioisosteric replacement of quinazoline to [1,2,4]triazolo[1,5-*a*]pyridin-6-yl could block oxidation on position 2 and 3 of quinazoline. To investigate the activity of the designed compounds, the group synthesized two series with imidazole or pyrazole central 5-membered rings. Two different tethers (methyleneamido and methylenethioamido) that connect the central 5-membered ring to the terminal phenyl ring were investigated. They were biologically evaluated on a purified human ALK5 kinase domain produced in a Sf9 insect cell kinase assay. The results demonstrated that in both the pyrazole and the imidazole central rings, the activity was improved by methylenethioamide compared to methyleneamide. Additionally, the differences in activity between the pyrazole and the imidazole central rings did not favor one over the other, so they could not conclude which ring is superior. Compound **80** with a pyrazole central ring had the most potent IC_50_ (0.018 μM) in the series (Figure 80). For the evaluation of the activation of ALK5 signaling induced by TGF-β transcriptional activation, HaCaT cells permanently transfected with p3TP-luciferase reporter construct were used for cell-based luciferase activity. Results showed, contrary to the kinase assay, a superior activity exhibited by methyleneamido derivatives compared to the methylenethioamido analogues. Yet, compound **80** had a higher inhibition (95% at 0.03 μM) compared to the other compounds in the series. Further biological evaluation aimed to investigate the selectivity of the compounds over the p38α MAP kinase since its kinase domain is known to be one of the most homologous to that of ALK5. The methyleneamido linkers did not show any activity against the p38α MAP kinase while imidazole containing methylenethioamido linkers did show inhibitory activity against it (IC_50_ of methylenethioamide compounds ranges from 1.05 μM to 5.21 μM). Compound **80** had the best selectivity towards ALK5 over the p38α MAP kinase among the series with a 284 selectivity index. The docking of compound **80** into the X-ray structure of ALK5 and superimposing it on the native ligands (1,5-naphthyrine inhibitor) somehow explained the reason behind the activity of compound **80** (Figure 89). The triazolopyridine ring interacts with the backbone *NH* of His 283 of the hinge region. Moreover, the *N* of the pyrazole ring forms a hydrogen bond with the protonated ammonium group of Lys232. Lastly, the cyano group accepts a hydrogen bond from Ser 287. Compound **80** fits the enzyme’s active site and interacts with the key amino acids in it [92].

### 26.6. Compound ***81***

**Figure 90:**
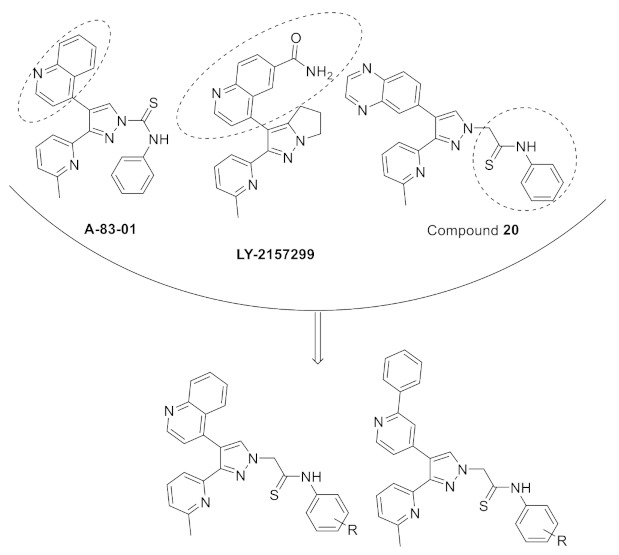
Schematic view of the rationale behind designing the series of compounds as ALK5 inhibitors.

ALK5 inhibitors hold abundant potential for a medicinal chemist to work on. Here, a similar group that worked on the development of compounds **78–80** report yet another study on possible changes and possible modifications that can be performed on the same framework that was used in the previous designs. The group described using a quinolin-4-yl arm which is similar to a previously reported inhibitor of ALK5, and explored the potential biological effect it has. They also planned to replace the quinolin-4-yl with an isostere, the 2-phenylpyridin-4-yl, investigating the depth of design that can be ascertained as a biologically useful agent (Figure 90).

The group synthesized a number of different series and evaluated their ALK5 inhibitory activity using a purified human ALK5 kinase domain produced in the Sf9 insect cell kinase assay at 10 μM, which showed that compound **81** had a promising inhibitory activity (2% residual activity at 10 μM, IC_50_ = 69 nM) (Figure 80). The series containing quinolin-4-yl arm was superior to the one with 2-phenylpyridin-4-yl. Further investigation was carried out on the p38α MAPK since its active site is highly homologous to ALK5’s. Most compounds were active against it, following the same trend seen in the ALK5 assay, and had compound **81**′s inhibitory activity at 10 μM as 3% residual activity and an IC_50_ of 104 nM.

### 26.7. Compound ***82***

Compound **82** has been reported as a potent and selective inhibitor of ALK5 (Figure 80). Its IC_50_ equals 30 nM. It is 235-fold more selective toward ALK5 than p38α. Moreover, it is 4-fold more potent than the clinical candidate LY-2157299. Compound **82** has also been reported as a potential inhibitor of collagen I and α-SMA protein, and mRNA expressions in TGFβ-induced LX-2 human hepatic stellate cells. Therefore, compound **82** is a potential candidate for treatment of hepatic fibrosis.

**Figure 91:**
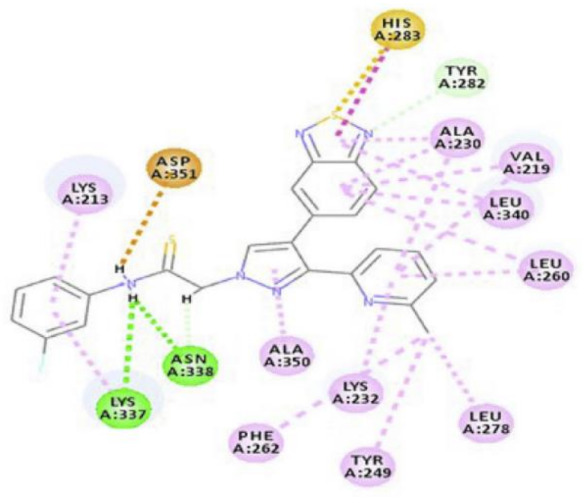
Two-dimensional putative binding interactions of compound **82** with ALK5 crystal structure [93].

The thioamide moiety of compound **82** is more favorable for activity than the corresponding amide. In addition, m-fluorophenyl is more optimal than any other aryl substituent. The docking study demonstrated a formation of hydrogen bonds between the thioamide *NH* and Lys337 and Asn338, in addition to a network of hydrophobic interactions performed by the pyrazole ring and the other rings of the structure (Figure 91) [93].

## 27. Pyrazole-Based Trk Inhibitors

### 27.1. Compounds ***83***–***85***

**Figure 92:**
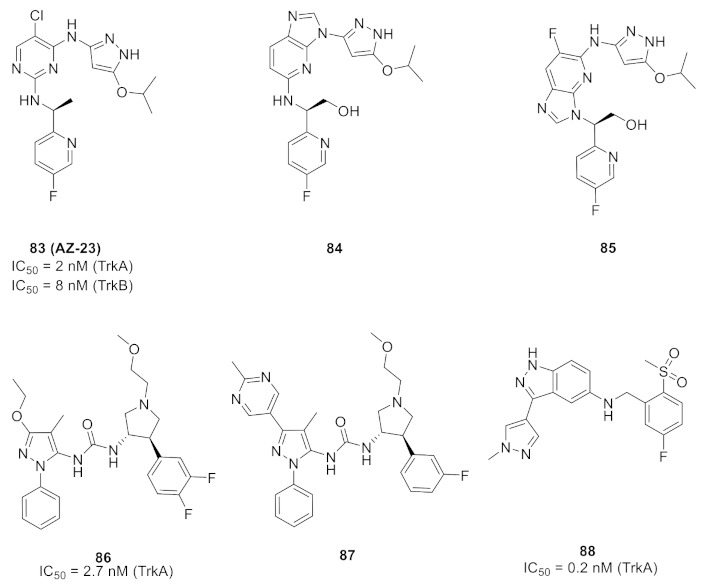
Structures of pyrazole-based TRK inhibitors and their IC_50_ values.

Compound **83** (AZ-23) is a known pyrazole-based ATP-competitive, orally bioavailable Trk kinase inhibitor. Its IC_50_ values against TrkA and TrkB are 2 and 8 nM, respectively (Figure 92). In addition, it inhibits other kinases such as FGFR1, FLT3, Ret, MUSK, and LCK [94].The pyrazole *N* and *NH* of AZ-23 form two hydrogen bonding interactions with Glu590 and Met592. In addition, the *NH* attached to the pyrazole ring donates a hydrogen bond to a backbone amide oxygen. Wang et al., optimized the structure of AZ-23 through ring fusion to get compounds **84** and **85** (Figure 92). Both compounds are potent inhibitors of TrkA with an IC_50_ value of 0.5 nM in a cellular assay. The extended hydroxyl group in both compounds forms an additional hydrogen bond with the backbone amide oxygen of Glu518 in the glycine-rich P loop. The oral bioavailability of both compounds **84** and **85** is 29% and 54%, respectively. In addition, their aqueous solubility values are 250 and 220 µM, respectively. The two compounds were tested against hERG and they were safe enough (IC_50_ values > 25 µM [95].

### 27.2. Compound ***86***

**Figure 93:**
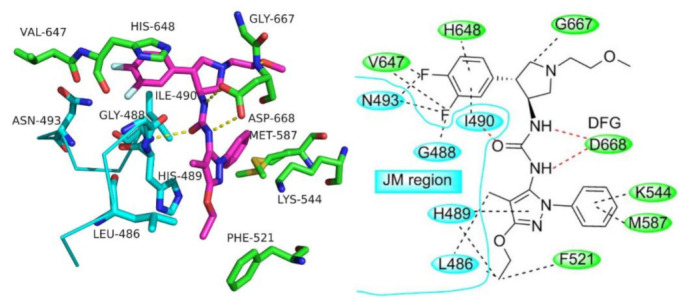
The in silico binding interactions of compound **86** with the juxtamembrane region of TrkA kinase [96].

Furuya et al., studied the important interactions of the inhibitor with the juxtamembrane region of the TrkA kinase for selectivity. They studied the interactions of compound **86** with that region (cyan color, Figure 93). The urea oxygen accepts a hydrogen bond from Ile490. In addition, the pyrazole ring with its methyl and methoxy substituents interacts with His489 and Leu486. Moreover, the difluorophenyl moiety interacts with Asn493, Gly488, and Ile490. The presence of moiety that interacts with the juxtamembrane moiety beyond the hinge region is crucial for selectivity. The Trk inhibitors that interact with the ATP-binding (hinge) region are usually non-selective. The selectivity of compound **86** towards TrkA was confirmed by the cell-free biochemical measurements. Its IC_50_ values against TrkA, TrkB, and TrkC are 2.7, 1303.7, and 2483.7 nM, respectively, which confirms its superior selectivity against TrkA [96].

### 27.3. Compound ***87***

Compound **87** is another pyrazolyl urea derivative that has a structural similarity to compound **86** (Figure 92). Compound **87** was developed with the aim of deciphering the allosteric binding mechanisms of TrkA inhibitors. It possesses the advantage of a rapid association rate with the TrkA crystal structure, thus binding to the inactive conformation of the kinase (i.e., type II TrkA inhibitor). In addition, its off-rate is slow [97].

### 27.4. Compound ***88***

Compound **88** was developed as a potent and peripherally restricted Trk kinase inhibitor for use as an analgesic agent (Figure 92). The main goal of the research group was to have a substrate for the efflux transporter, thus it has a low CNS penetration ability and a higher plasma exposure. The IC_50_ values of compound **88** in cell-free and cell-based Trk kinase assays are 0.2 and 1.7 nM, respectively. It was further tested at 20 nM concentration against a panel of 49 kinases and exerted selectivity against TrkA. Replacement of methylpyrazole with methoxypyridine, the insertion of NHSO_2_Me instead of the methyl group, and the removal of fluoro led to less potency against TrkA (IC_50_ = 7 and 53 nM in cell-free and cell-based assays, respectively). Furthermore, compound **88** was tested in vivo in Complete Freund’s adjuvant (CFA)-induced thermal hypersensitivity model. Compound **88** at a 4 mg/kg oral dose gave anti-pain activity comparable to ibuprofen (100 mg/Kg p.o. dose). The clearance of compound **88** is 127.4 mL/min/kg and its aqueous solubility is 140.7 µM (compared to 15.2–23 µM when there is pyridyl instead of methylpyrazole). After oral administration of a 5 mg/kg of compound **88** in rats, its C_max_ was 161 nM. In addition, its t_1/2_ was 1.46 h following the i.v. administration of 1 mg/kg in rats. The ratio of compound **88** in brain to plasma was found to be only 0.03 after an i.p. injection of 2.5 mg/kg in rats [98].

## 28. Pyrazole-Based VEGFR Kinase Inhibitors

### 28.1. Compound ***89***

**Figure 94:**
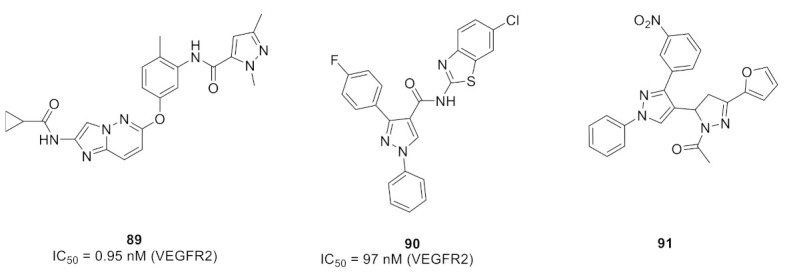
Structures of pyrazole-based VEGFR2 inhibitors and their IC_50_ values.

The high expenditure of energy, nutrients and oxygen required by tumor cells to survive and grow is met through growing new blood vessels, a process named angiogenesis. Multiple proteins are involved in angiogenesis, but vascular endothelial growth factor receptors (VEGFRs) have been the center of interest as drug targets for their great involvement in tumor neovascularization [99]. Compound **89** was part of a series designed to inhibit the VEGFR-2 kinase (Figure 94). It strongly inhibited the VEGFR-2 kinase (IC_50_ = 0.95 nM) and decreased the proliferation of VEGF-stimulated human umbilical vein endothelial cells (HUVEC) with an IC_50_ of 0.30 nM. The consequences of bioisosteric replacement of amides as well as the tethered phenyl moieties revealed that a smaller aliphatic cyclic system such as cyclopropyl is optimum for activity, heterocyclic pyrazole and generally aromatic systems are better compared to aliphatic cyclic systems, and finally the 2,methylphenyl is optimum for activity compared to other substituents in the tethered phenyl ring. Its kinase selectivity profiling over 250 kinases indicated that compound **89** inhibits VEGFR-1 (IC_50_ = 3.2 nM), VEGFR-3 (IC_50_ = 1.1 nM), PDGFR-α and β (IC_50_ = 4.3 nM and 13 nM, respectively), FMS (IC_50_ = 10 nM) and RET (IC_50_ = 18 nM) kinases while its IC_50_ values against other kinases were above 100 nM. Oral administration of 1 mg/kg twice daily suppressed tumor growth in a mouse xenograft model of human lung adenocarcinoma A549 cells (T/C = 8%) [100].

### 28.2. Compound ***90***

Using pyrazole-containing chemotherapeutic agents like crizotinib, axitinib and ibrutinib as a framework, a series of pyrazole-benthothiazole hybrids were designed as antiangiogenic agents. Compound **90** bearing halogens (fluoro and chloro) as substituents, displayed in vitro inhibitory activity against VEGFR-2 (Figure 94). Compound **90** was investigated for cytotoxic activity using the MTT assay against the HT-29 colon cell line (IC_50_ = 3.32 μM), PC-3 prostate cells (IC_50_ = 3.17 μM), A549 lung cells (IC_50_ = 3.87 μM), and U87MG glioblastoma cells (IC_50_ = 6.77 μM). It was also tested against a normal human embryonic kidney cell line (HEK-293T) to investigate selectivity towards tumor cells and revealed 9 to 15-fold more selectivity towards cancer cells compared to axitinib, which is only 2–3 times more selective. The SAR study presented by this series revealed the superiority of electron-withdrawing substituent compared to the electron-donating ones. Further studies such as the colony-forming potential of PC-3 treated with compound **90** showed an inhibition of compound **90** at 1, and 5 μM for 7 days, crossed multiple layers of both PC-3 and U87MG spheroids at 1 μM and 5 μM in a 3D multicellular spheroids inhibition assay, inhibited migration in PC-3 cells at 3 μM after 48h as compared to control cells, and almost stopped migration at 5 μM and dose-dependent G_0_/G_1_ phase cell cycle arrest in PC-3 cells using the flow cytometry analysis. The in vivo antiangiogenic activity was analyzed using Zebrafish embryos treated with compound **90** at 0.1, 0.5 and 1 μM with axitinib used as a positive control 24 h post fertilization (hpf) stage, and it revealed that compound **90** exhibits dose-dependent antiangiogenic activity [101].

### 28.3. Compound ***91***

A bi-aryl pyrazole series conjugated with pyrazoline, triazolopyrimidine, and pyrazolone compounds was designed as an antiangiogenic agent, targeting the VEGFR-2 kinase. The best compound of this series in terms of potency was compound **91** (Figure 94) which had in an SRB assay against the MCF-7 cell lines, an IC_50_ of 18.35 μM in comparison to tamoxifen, which has an IC_50_ of 23.31 μM. It also displayed a reduction in the VEGFR-2 levels in the MCF-7 cell lines with 72% inhibition compared to an untreated control. Compared to sorafenib in an ELISA assay, compound **91** had an IC_50_ of 225.13 nM against the VEGFR-2 kinase, while sorafenib’s was 186.54 nM. Compound **91** was the most potent in the series [102].

**Figure 95:**
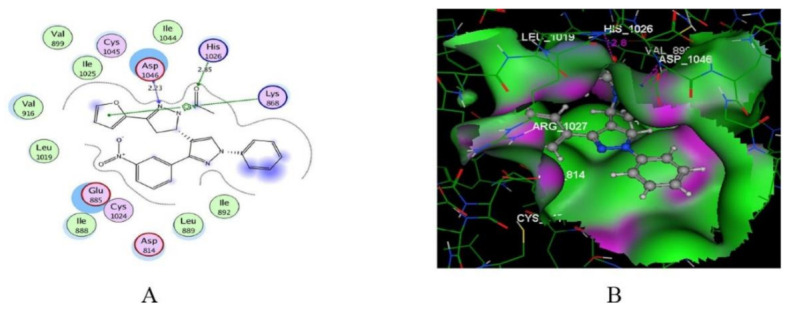
Compound **91** binding mode in the active pocket of VEGFR-2, (**A**) illustrates the 2D interaction and (**B**) the 3D interactions. Green = hydrophobic area, pink = high polar area, blue = mild polar area and dotted lines and arrows = hydrogen bonds [102].

The docking of compound **91** into the VEGFR-2 active pocket was done on the MOE 2008.10 version. The docking results displayed the key interaction of the arms of the pyrazole core, and the contribution made by the *N*-acetyl pyrazoline and its interactions with different amino acids of the active site. Compounds with the *N*-acetyl moiety had superior results as compared to the other compounds in this series. The furan centroid interaction with Lys868 could explain the VEGFR-2 inhibitory activity (Figure 95) [102].

## 29. Pyrazole-Based Multikinase Inhibitors

### 29.1. Compound ***92***

**Figure 96:**
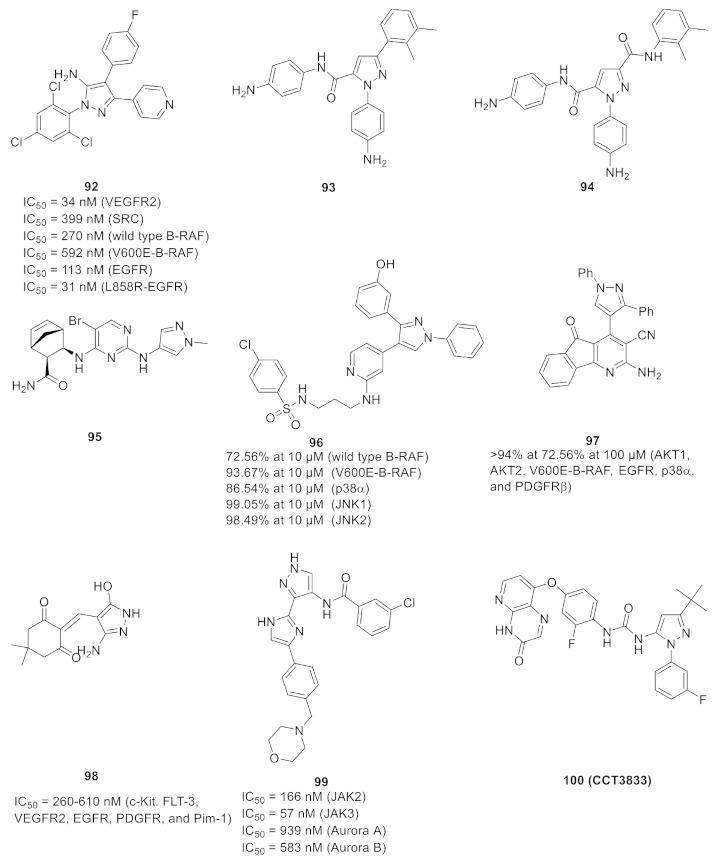
Structures of pyrazole-based multi-kinase inhibitors and their IC_50_ values or percentage inhibition.

The 1,3,4-triarylpyrazole derivatives with a *p*-fluorophenyl at position 3 of the pyrazole ring and 4-pyridyl at position 4 are potent inhibitors of p38α. Upon a regioisomeric switch from 3-(4-fluorophenyl)-4-(pyridin-4-yl)-1-(aryl)-1*H*-pyrazol-5-amine to 4-(4-fluorophenyl)-3-(pyridin-4-yl)-1-(aryl)-1*H*-pyrazol-5-amine, i.e., an exchange of the locations of the pyridyl and fluorophenyl rings, p38α inhibition was lost, but the new analogues inhibited other kinases over-expressed in tumors. Compound **92** is the most promising kinase inhibitor among this series (Figure 96). It is a multiple inhibitor of VEGFR2, Src, B-RAF (wild-type), V600E-B-RAF, EGFR (wild-type), and L858R-EGFR with IC_50_ values of 34, 399, 270, 592, 113, and 31 nM, respectively. The 2,4,6-trichlorophenyl, 4-fluorophenyl, 4-pyridyl, and the amino group are all together important for the kinase inhibitory effect. Removal of amino, replacement of fluorophenyl with amide or ester, or replacement of the trisubstituted phenyl with a monosubstituted one led to decreased potency against the kinases [103].

**Figure 97:**
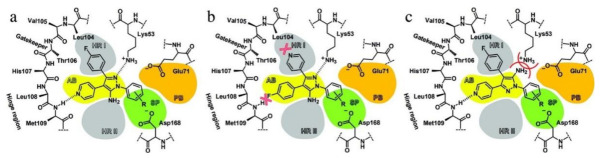
(**a**) Binding interactions of the regioisomer of compound **92** with p38α. (**b**,**c**) Effects of regioisomerism on declined activity against p38α [103].

**Figure 98:**
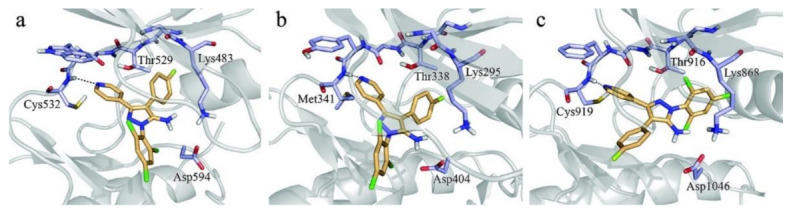
Putative binding interactions of compound **92** with B-RAF (**a**), Src (**b**), and VEGFR2 (**c**) [103].

If the pyridyl ring is attached to position 4 of the central pyrazole ring, its nitrogen atom comes at the right location to accept a hydrogen bond from Met109 in the hinge region of p38α (Figure 97a). The ring switch in compound **92** shifts the pyridyl nitrogen away from Met109, thus justifying the weak inhibitory effect against p38α (Figure 97b). Moreover, even if the pyridyl ring interacts with Met109, the amino group of compound **92** comes in front of the protonated amino of Lys53 and this leads to repulsion (Figure 97c) [103]. Figure 98 illustrates its binding interactions with B-RAF, Src, and VEGFR2 [103].

### 29.2. Compounds ***93*** and ***94***

Both compounds **93** and **94** are antiproliferative agents against the SNU449 hepatocellular carcinoma cell line but with modest potency (IC_50_ 50–100 µM) (Figure 96). In silico studies revealed their potential multikinase inhibitory effects against AKT2, GSK-3β, PI3K, EGFR, IGFR, CDK2, Aurora A, and MAPK. Docking studies demonstrated the formation of 2–3 hydrogen bonds by the terminal amino, the C5-amide *O* and the *NH* groups. The pyrazole ring and/or the aryl rings interact with the phenyl rings of the Phe or Tyr amino acid residues to form pi-pi interactions [104].

Western blot assays showed that both compounds inhibit the PI3K/AKT/mTOR pathway resulting in a down-regulation of the phosphorylated isoform of both direct and indirect downstream targets such as GSK-3β, ribosomal subunit S6 and MDM2. However, upon testing in real experiments against a 20-kinase panel at 3 µM concentration, no significant inhibition was recorded. The authors recommend kinase testing at high concentrations and we recommend further lead optimization [104].

### 29.3. Compound ***95***

**Figure 99:**
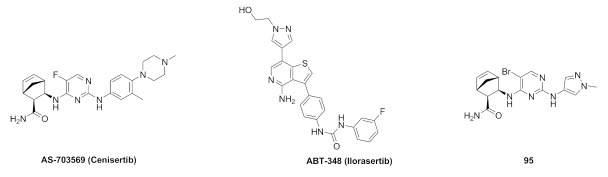
Structures of cenisertib, illorasertib, and compound **95**.

A series of novel pyrazole derivatives that are structurally related to kinase inhibitor AS-703569 (cenisertib, Figure 99) were developed in an effort to identify kinase inhibitors with dual KDR/Aurora B activity and enhanced aqueous solubility compared to Abbott’s dual inhibitor ABT-348 (ilorasertib) (Figure 99). Compound **95** was found to have a balanced and strong potency against the two kinases (Figure 96 and Figure 99). Compound **95** is also a potent inhibitor of many RTKs and serine/threonine kinases. In fact, this compound has a K_i_ < 5 nM for thirty-eight kinases, a K_i_ of 5–10 nM over eleven kinases, and a K_i_ of 10–20 nM against sixteen kinases. The pan-kinase inhibitory effects of this compound led to a narrow therapeutic index that prohibited its use as an anticancer agent. This is applicable to that series of compounds: the derivatives of compound **95** [105].

### 29.4. Compound ***96***

Compound **96** is the most promising antiproliferative derivative among a series of triarylpyrazoles containing terminal aryl sulfonamide moiety (Figure 96). It was tested against the NCI-60 cancer cell line panel and exerted broad-spectrum activity with a 97.80% mean inhibition percentage. The A498 renal carcinoma cell line is the most sensitive cell line to compound **96** (IC_50_ = 0.33 µM). Upon testing against a 20-kinase panel, it exerted inhibitory effects against B-RAF (wild-type), V600E-B-RAF, p38α, JNK1, and JNK2 kinases (inhibition % values at 10 µM concentration are 72.56%, 93.67%, 86.54%, 99.05%, and 98.49%, respectively). JNK1 and JNK2 are the most sensitive among them (IC_50_ = 350 and 360 nM, respectively). The plasma stability testing of compound **96** showed its high stability profile in both human and rat plasma. However, its oral bioavailability following 10 mg/kg administration in rats is only 9.2%. The SAR study demonstrated that the phenolic *OH*, propylene spacer, and terminal p-chlorobenzene sulfonamide moieties are optimal for the activity of this compound compared to methoxy, ethylene linkers, and other aryl sulfonamide motifs [106].

### 29.5. Compound ***97***

A series of novel 1,3,4-triarylpyrazole derivatives attached to a tricyclic ring system was reported as multiple kinase-inhibiting antiproliferative agents. Compound **97** is the most promising among them (Figure 96). It was tested against five different cancer cell lines, but its highest potency was exerted against the MCF7 breast cancer cell line (IC_50_ = 6.53 µM). The fused tricyclic ring system with carbonyl was found to be more favorable for antiproliferative activity than a fused bicyclic, monocyclic or even a fused tricyclic ring system lacking a carbonyl group. Compound **97** was further tested against a panel of 12 kinases at 100 µM and was promiscuous. Compound **97** showed more than 94% inhibition against AKT1, AKT2, V600E-B-RAF, EGFR, p38α, and PDGFRβ. The highest activity was against EGFR (99% inhibition) [107].

**Figure 100:**
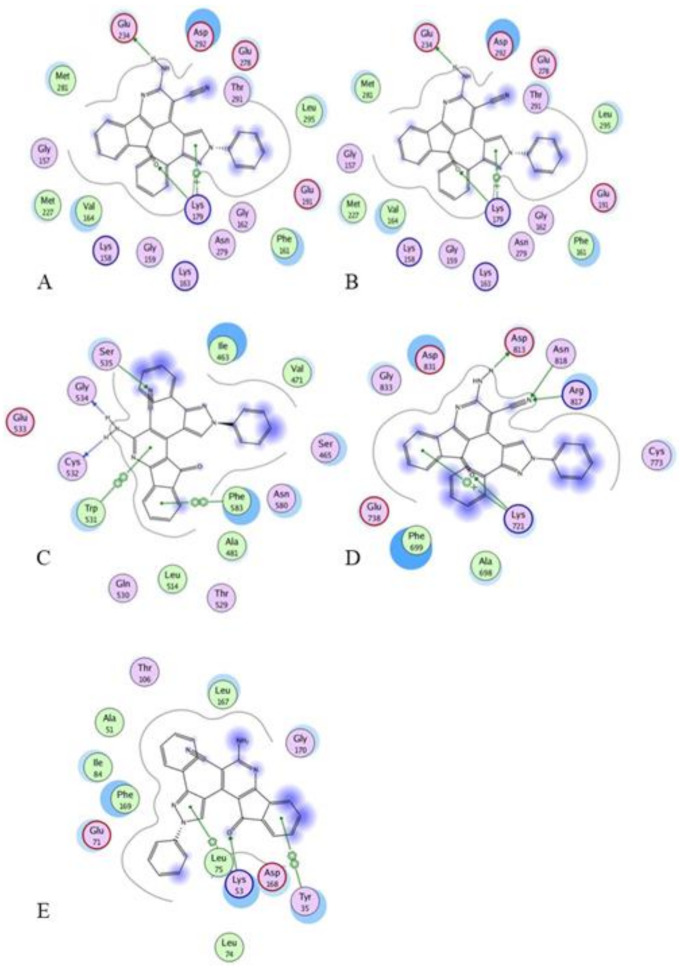
Putative binding interactions of compound **97** with AKT1 (**A**), AKT2 (**B**), V600E-B-RAF (**C**), EGFR (**D**), and p38α kinases (**E**) [107].

Docking studies were performed in order to study its binding interactions with AKT1, AKT2, V600E-B-RAF, EGFR, and p38α kinases (Figure 100). The pyrazole ring forms arene–cation interactions with the Lys amino acid residues of the AKT1 and p38α kinases. However, it forms hydrophobic interactions with the Phe163 residue of AKT2. The carbonyl oxygen accepts hydrogen bonds in all kinases except V600E-B-RAF. The cyano and amino groups act as additional hydrogen bond-forming groups, and the fused benzene and pyridine rings form hydrophobic interactions with the EGFR crystal structure. This can rationalize the strong inhibitory effect of compound **97** against the EGFR kinase [107].

### 29.6. Compound ***98***

Compound **98** is a dimedone-pyrazole hybrid that was reported as multikinase inhibitory antiproliferative agent (Figure 96). It was tested against the A549 (lung), H460 (lung), HT29 (colon), MKN-45 (gastric), U87MG (glioma), and SMMC-77217721 (hepatic) cancer cell lines and showed sub-micromolar IC_50_ values within the range of 0.29–0.42 µM. It was also tested against c-Kit, FLT-3, VEGFR-2, EGFR, PDGFR, and Pim-1 kinases and inhibited all of them with IC_50_ values of 260–610 nM. The highest potency was exerted against Pim-1 (IC_50_ = 260 nM). Phenyl substitution on the *NH* of pyrazole ring or replacement of the amino group with hydroxyl decreased the activity [108].

### 29.7. Compound ***99***

**Figure 101:**
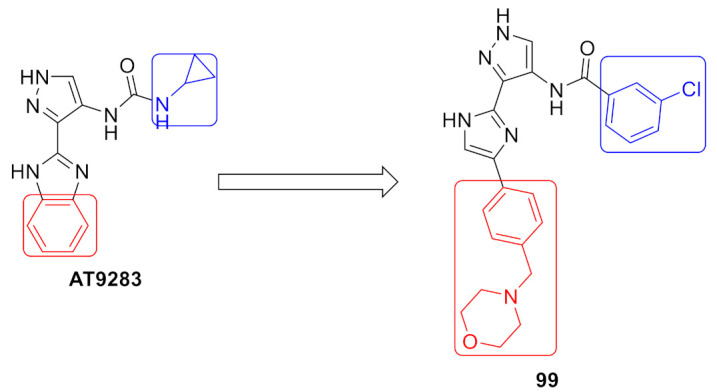
Structures of **AT9283** and compound **99**.

A series of pyrazole derivatives was reported as multikinase inhibitory antiproliferative agents. These derivatives were designed with a similarity to AT9283, a pyrazolyl urea derivative with JAK/Aurora kinase inhibitory effects (Figure 101). Compound **99,** possessing *m*-chlorobenzamido moiety, exerted more balanced biological results against kinases and cancer cell lines compared to other analogues with substituents except for chloro (Figure 96). The IC_50_ values of compound **99** against JAK2, JAK3, Aurora A, and Aurora B are 166, 57, 939, 583 nM, respectively. Its potency against these kinases is weaker than AT9283 (2.2, 1.2, 26, and 62 nM, respectively). In addition, compound **99** was tested against the K562 leukemia cell line and the HCT116 colon cancer cell line (IC_50_ = 6.726 and 15.054 µM, respectively) but yielded weaker potency than AT9283 (IC_50_ = 0.748 and 0.09 µM, respectively). Against the HCoEpiC and HUVEC normal cells, the IC_50_ values of compound **99** are 31.509 and 28.978 µM, respectively while those of AT9283 are 2.367 and 1.793, respectively. Upon testing the effects of compound **99** on K562 and HCT116 cell cycles, it arrested the G2 phase in a dose-dependent manner [109].

**Figure 102:**
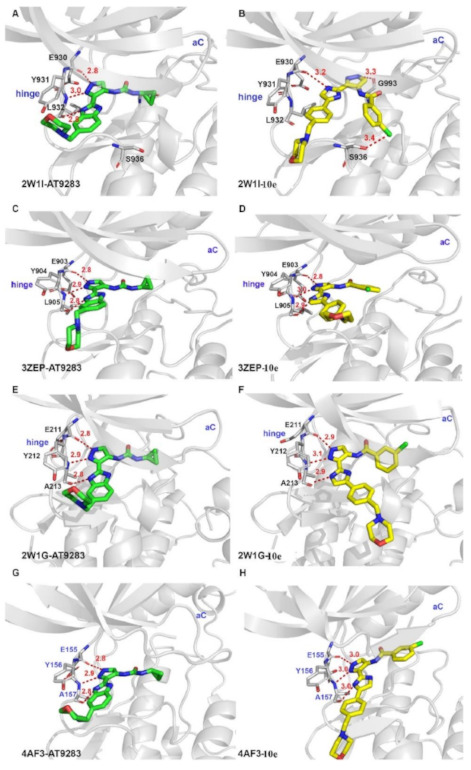
Putative binding interactions of AT9283 and compound **99**. (**A**) AT9283 with JAK2; (**B**) Compound **99** with JAK2; (**C**) AT9283 with JAK3; (**D**) Compound **99** with JAK3; (**E**) AT9283 with Aurora A; (**F**) Compound **99** with Aurora A; (**G**) AT9283 with Aurora B; (**H**) Compound **99** with Aurora B [109].

The docking study of compound **99** and AT9283 against the four kinases was carried out (Figure 102). The pyrazole and imidazole rings of compound **99** formed a network of hydrogen bonds with the hinge regions of the four kinases. These interactions are similar to AT9283 but weaker. This explains the weaker potency of compound **99** against the kinase when compared to AT9283. It is noteworthy that the morpholino ring of compound **99** did not contribute to interactions with the kinase crystal structures, and the structure of AT9283 does not possess a similar moiety [109].

### 29.8. Compound ***100***

Compound **100** (CCT3833) is a pyrazolyl urea-based pan-RAF kinase inhibitor that possesses inhibitory effects against the Src kinase as well (Figure 96). It inhibited B-RAF, C-RAF, and Src kinases both in vitro and in vivo. In addition, it decreased tumor regression in mouse models. CCT3833 is a clinical candidate that was evaluated in phase I clinical trials for tolerability and safety in volunteer patients with solid tumors [110]. In addition, CCT3833 was reported to increase the progression-free survival in a patient suffering from KRAS (G12V) spindle cell sarcoma [111,112].

## 30. Conclusions

Pyrazole represents a privileged scaffold with a variety of therapeutic activities. In this article, we reviewed different pyrazole-based kinase inhibitors that were described in the literature in the last decade (2011–2020). During the last decade, pyrazole derivatives were reported to exert a wide spectrum of biological activities including antimicrobial and anticancer activities among others. In the anticancer and anti-inflammatory fields in particular, pyrazoles have been shown to interact at the intracellular level on many pathways, especially against kinases. As reported in the literature from 2011 to 2020, several important results have been obtained, where pyrazole derivatives have been shown to inhibit many kinases such as the Akt, ALK, Aurora, Bcr-Abl, CDK, Chk2, EGFR, ERK/MEK, FGFR, IRAK4, ITK, JAK, JNK, LRRK, Lsrk, MAPK14, PDK4, Pim, RAF, ROS1, Src, VEGFR, and others in the pathways of different diseases. The reviewed compounds were classified according to their kinase targets with some of them being multiple kinase inhibitors. We arranged the compounds chronologically from the oldest to the most recent in each kinase category. Structural modifications of pyrazole derivatives can be optimized to enhance potency, selectivity, and pharmacokinetic properties. The pyrazole ring can replace more hydrophobic (hetero)aromatic rings in the structure to improve aqueous solubility and PK properties. It can also act as a core scaffold for proper orientation of other rings/substituents attached to it inside the kinase crystal structure. The nitrogen atoms of pyrazole can perform hydrogen bonding and its carbons can contribute to hydrophobic interactions. Pyrazole is also an electron-rich ring, so it can contribute to an arene–cation interaction if it comes in front of positively charged amino acid residues in kinase crystal structures. The design and development of pyrazole derivatives as kinase inhibitors have been interesting in research in the field of medicinal chemistry. Azole derivatives including pyrazoles have a potential to inhibit cytochrome P450 enzymes, thus it is recommended for researchers working in this field to test their potential compounds against the CYP450 isozymes. If a significant inhibitory effect is found, the structural design should be reconsidered to decrease affinity to CYP450 with special attention given to decreasing electron density on the pyrazole ring by attaching electron-withdrawing group(s), for example, or replacement of pyrazole with 6-membered ring keeping in mind the possible impact on biological activity. Otherwise, re-adjustment of the doses of co-administered drugs that are metabolized mainly by cytochrome P450 enzymes is recommended to avoid accumulation and toxicity.

Table 1 summarizes the structures of the reviewed pyrazole-based kinase inhibitors, their IC_50_ values against the most sensitive kinases in cell-free assays, and other relevant biological activities.

## Figures and Tables

**Table 1:** Structures, IC_50_ values, and the most important biological results of the reviewed pyrazole-based kinase inhibitors.

Kinase	Inhibitor	IC_50_ Value in Cell-Free Assay (nM)	Other Biological Activities
AKT1	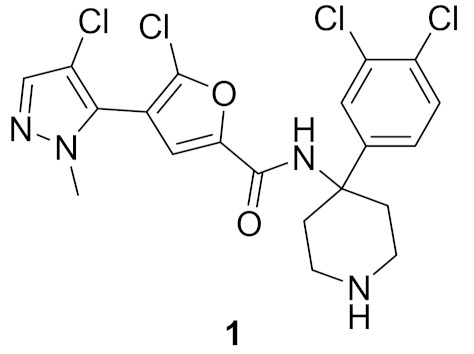	61	Antiproliferative activity against HCT116 and OVCAR-8 cell lines (IC_50_ = 7.76 and 9.76 µM, respectively)
	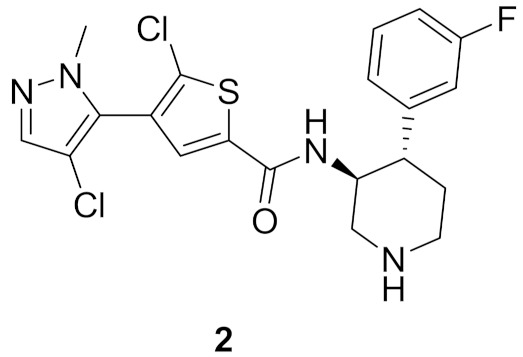	1.3	Antiproliferative activity against HCT116 colon cancer cell line (IC_50_ = 0.95 µM). Reduction of tumor size by 42% in the MM1S model.
ALK	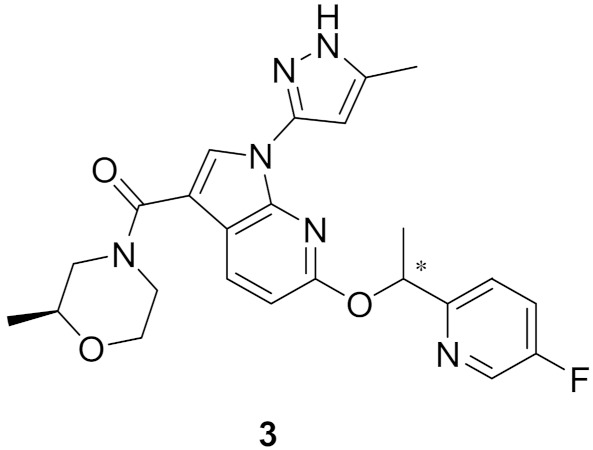	2.9	Reduction of phosphorylation of ALK in hippocampus in a dose-dependent manner at 30 mg/kg and higher. Inhibited phosphorylation in prefrontal cortex at 100 mg/kg.
ASK1	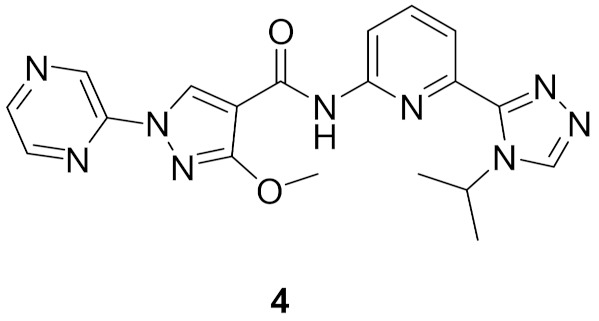	-	Good CNS penetration. Weak potency against hERG, CYP3A4, and CYP2C9.
Aurora	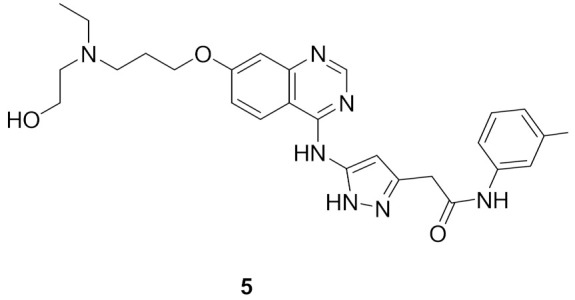 (Barasertib, AZD1152)	0.37 (Aurora B)	Passed phase I clinical trials in Japanese and Western volunteers suffering from advanced acute myeloid leukemia.
	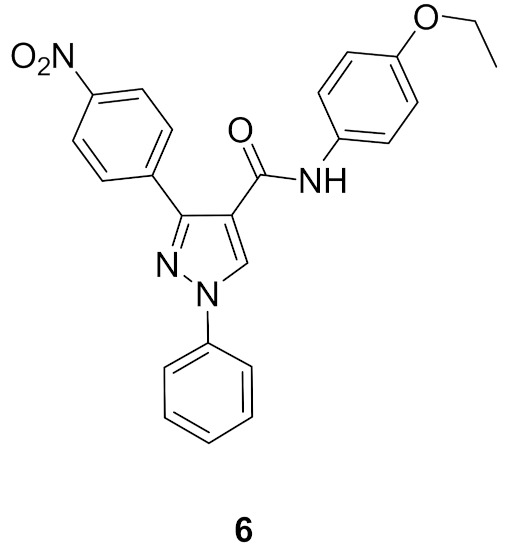	160 (Aurora A)	IC_50_ values against HCT116 colon cancer and MCF7 breast cancer cell lines are 0.39 and 0.46 µM, respectively.
	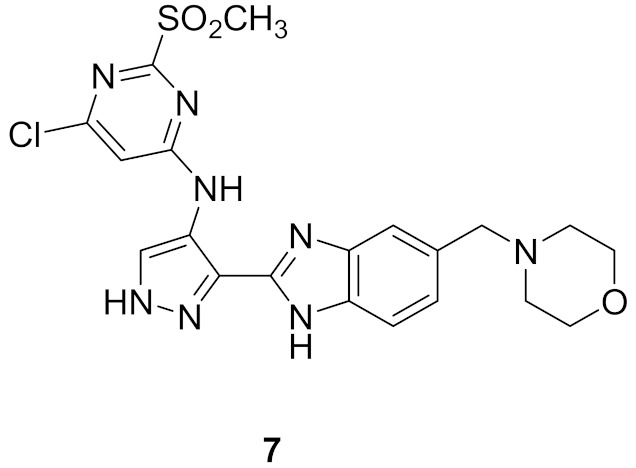	28.9 (Aurora A) 2.2 (Aurora B)	IC_50_ values against U937 (leukemia), K562 (leukemia), A549 (lung), LoVo (colon), and HT29 (colon) cancer cell lines are 5.106, 5.003, 0.487, 0.789, and 0.381 µM, respectively.
	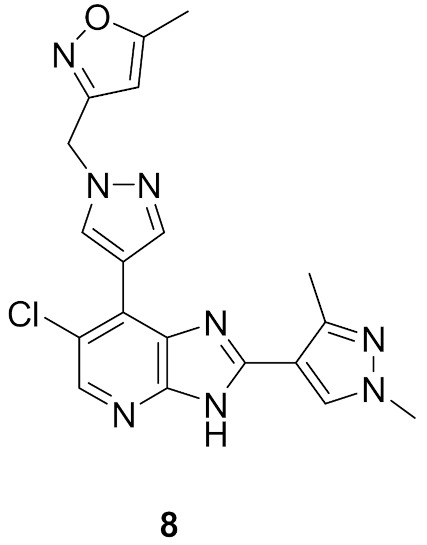	35 (Aurora A) 75 (Aurora B)	Antiproliferative activity against SW620 and HCT116 colon cancer cell lines (IC_50_ = 0.35 and 0.34 µM, respectively).
	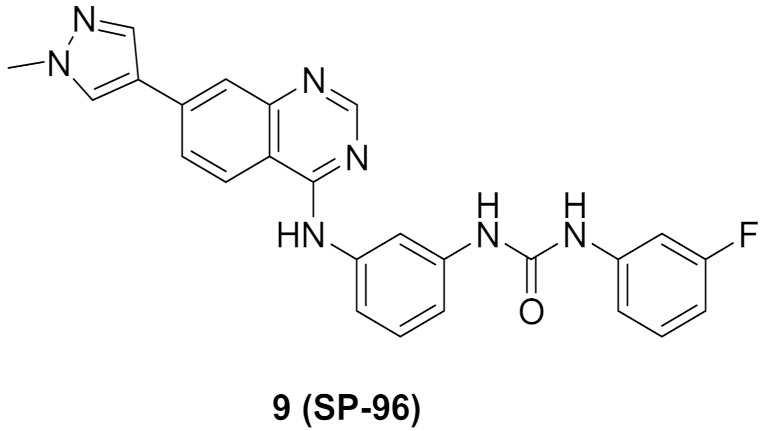	0.316 (Aurora B)	Antiproliferative activity against MDA-MB-468 with IC_50_ value equal to 107 nM.
BCR-ABL	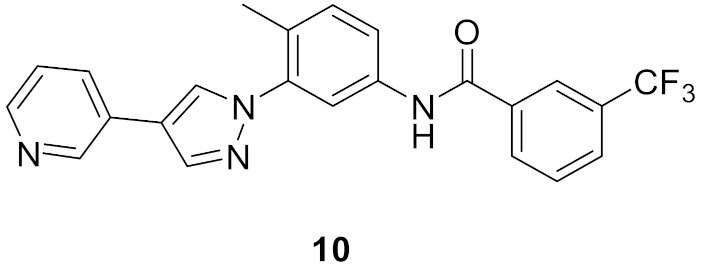	14.2	Antiproliferative activity against the K562 leukemia cell line with an IC_50_ value equal to 0.27 µM.
	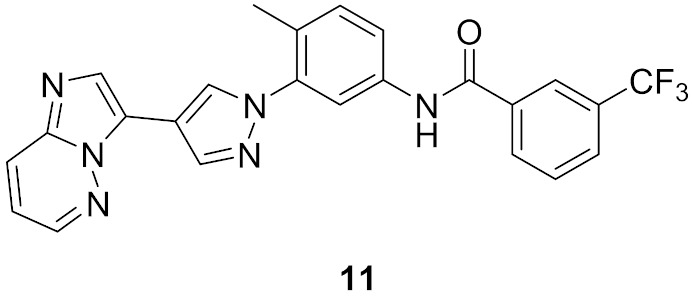	8.5	Antiproliferative activity against the K562 leukemia cell line with an IC_50_ value less than 2 nM.
	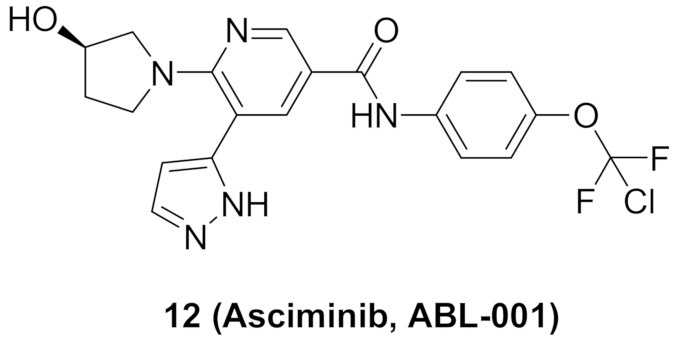	0.5	IC_50_ = 25 nM against ABL (T315I). Clinical candidate for CML.
Calcium-dependent kinase	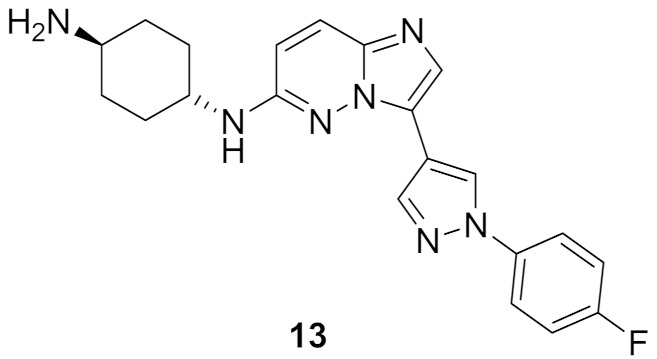	56	Anti-parasitic activity against *Plasmodium falciparum* with an IC_50_ value of 0.262 µM.
	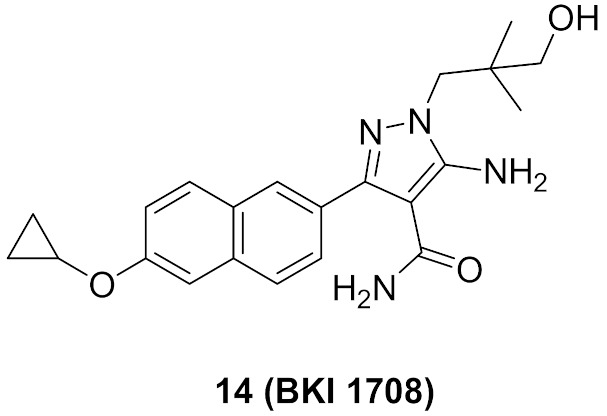	0.7	Anti-parasitic activity against *Cryptosporidium parvum* with an EC_50_ value of 0.41 µM.
	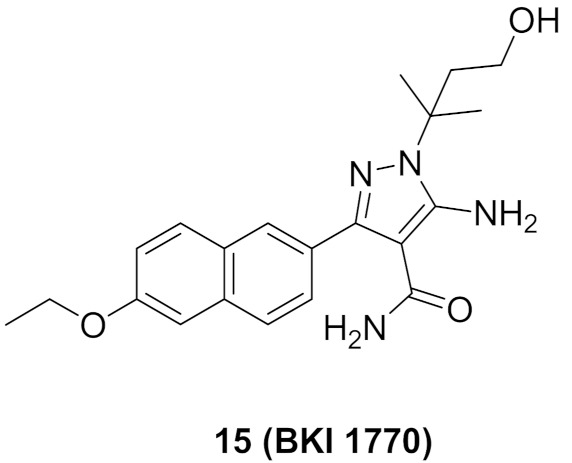	2.5	Anti-parasitic activity against *Cryptosporidium parvum* with an EC_50_ value of 0.51 µM.
Checkpoint kinase 2	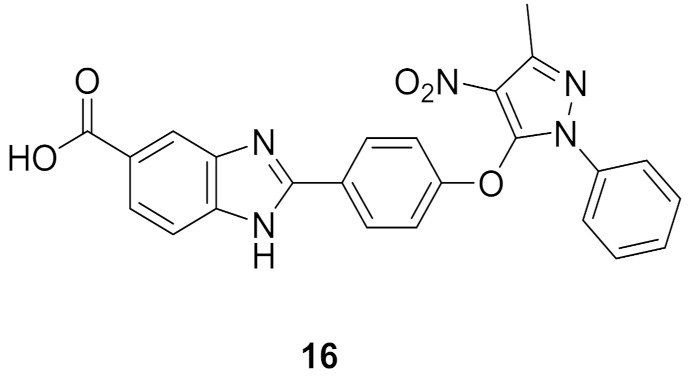	48.4	Antiproliferative activity against HepG2 (hepatocellular carcinoma), HeLa (cervical), and MCF7 (breast) cancer cell lines.
	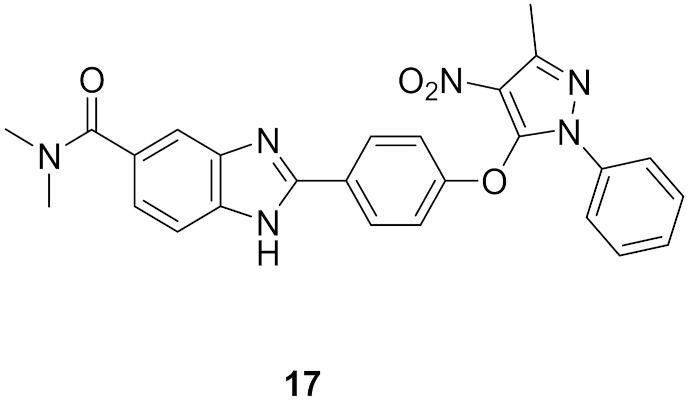	17.9	Antiproliferative activity against HepG2 (hepatocellular carcinoma), HeLa (cervical), and MCF7 (breast) cancer cell lines (IC_50_ = 10.8, 11.8, and 10.4 µM, respectively).
	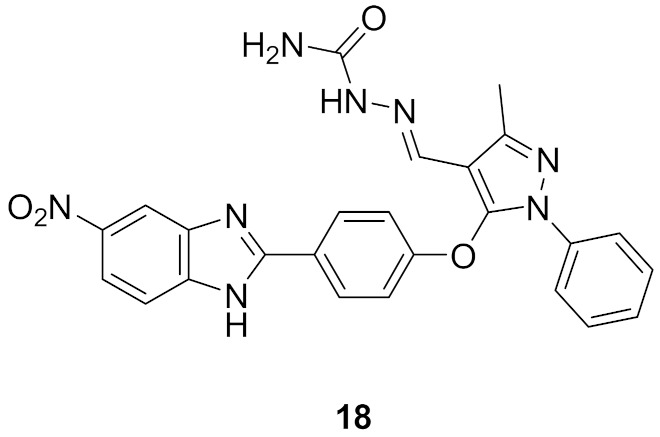	41.64	Modest potency against HepG2, HeLa, and MCF7 cell lines with 2-digit micromolar IC_50_ values.
Cyclin-dependent kinases	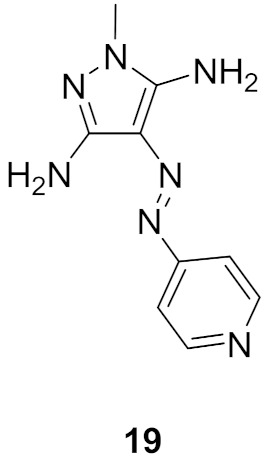	420 (CDK4)	Modest antiproliferative activity against K562, MCF7, and RPMI-8226 cancer cell lines. Induced apoptosis in RPMI-8226 cells.
	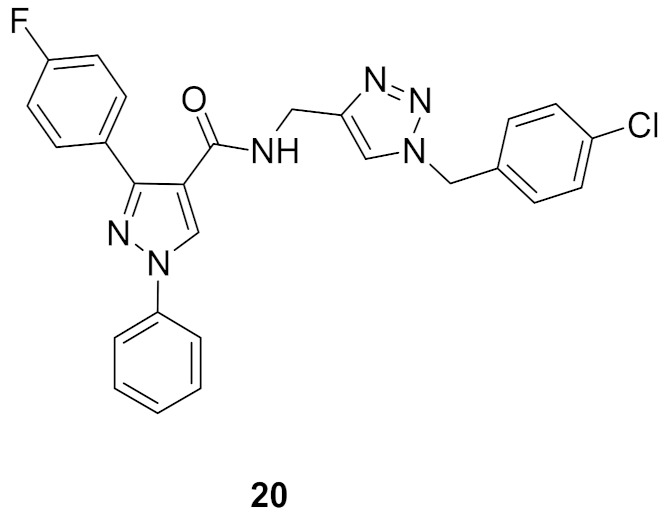	-	IC_50_ values against MCF7 cells (IC_50_ = 0.13 µM), MIAPaCa pancreatic cancer cell line (IC_50_ = 0.28 µM), and HeLa cervical cancer cell line (IC_50_ = 0.21 µM).
	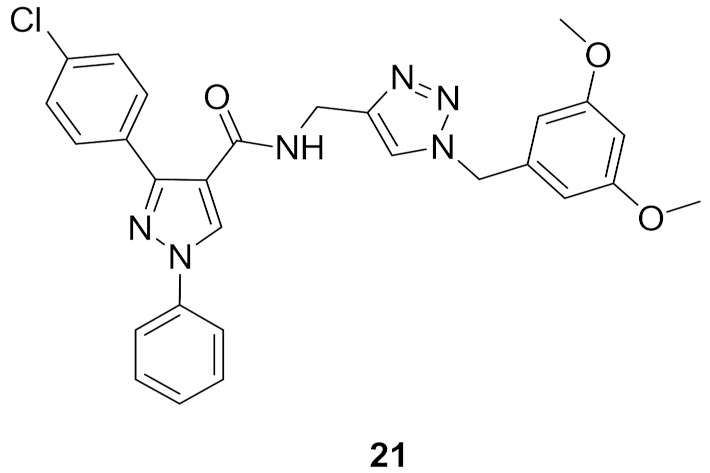	-	IC_50_ values against MCF7 cells (IC_50_ = 0.15 µM), MIAPaCa pancreatic cancer cell line (IC_50_ = 0.34 µM), and HeLa cervical cancer cell line (IC_50_ = 0 0.73 µM).
	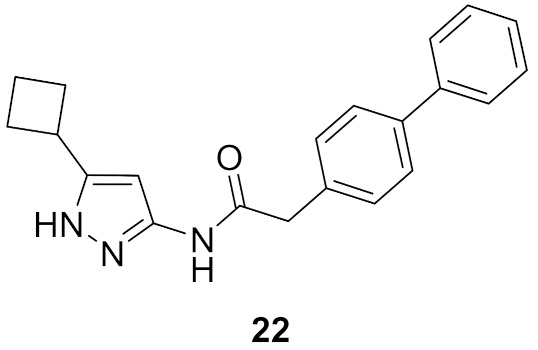	24 (CDK2) 23 (CDK5)	Induction of apoptosis in the MiaPaCa2 pancreatic cancer cell line.
	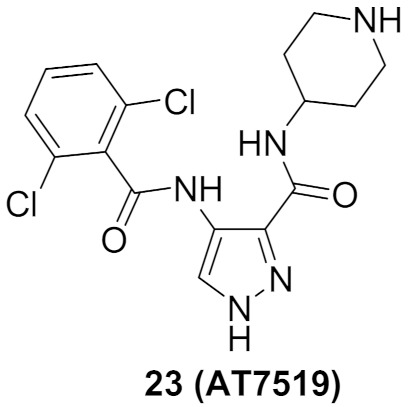	10–210 (multiple CDK inhibitor)	Induction of apoptosis in colon cancer and multiple myeloma cells.
	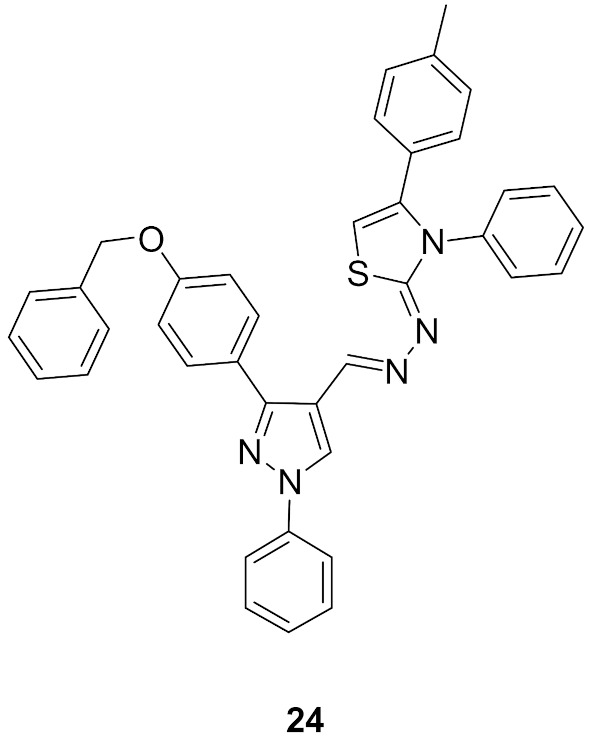	2380 (CDK1)	Antiproliferative activity against hepatocellular carcinoma (HepG2, Huh7, and SNU-475), colon cancer (HCT116), and renal cancer (UO-31) cell lines (IC_50_ = 0.05, 0.065, 1.93, 1.68, and 1.85 µM, respectively).
	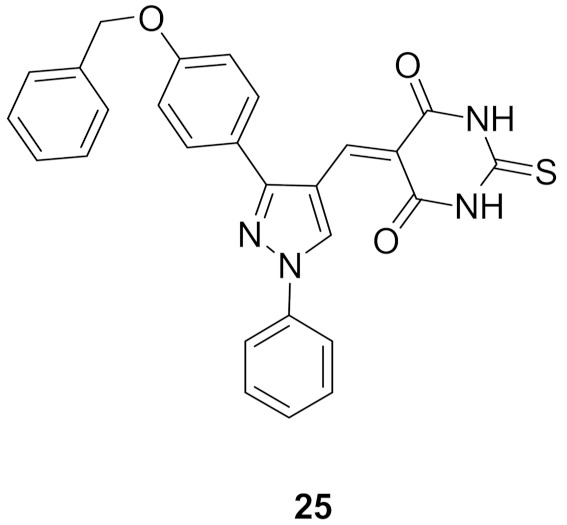	1520 (CDK1)	Antiproliferative activity against hepatocellular carcinoma (HepG2, Huh7, and SNU-475), colon cancer (HCT116), and renal cancer (UO-31) cell lines (IC_50_ = 0.028, 1.83, 1.70, 0.035, and 2.24 µM, respectively).
	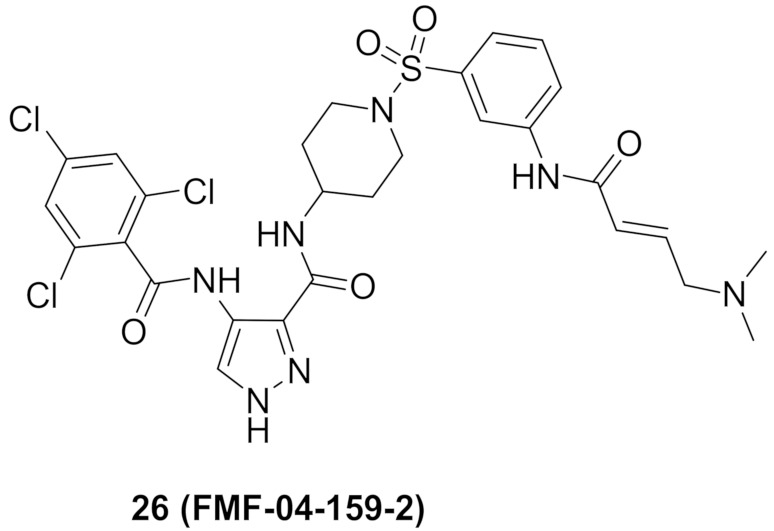	88 (CDK14)	Antiproliferative activity against HCT116 colorectal cancer cell line (IC_50_ = 1.14 µM).
	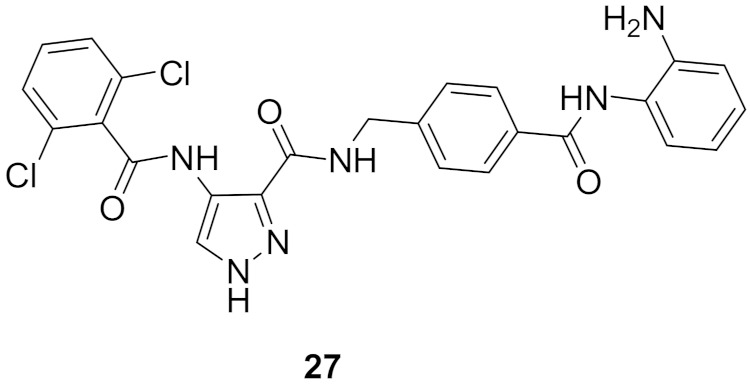	8.63 (CDK1) 0.30 (CDK2)	Inhibitory effect against HDAC1, HDAC2, and HDAC3 (IC_50_ = 6.40, 0.25, and 45.0 nM, respectively). Antiproliferative activity against HCT116 colorectal cancer cell line (IC_50_ = 0.71 µM).
	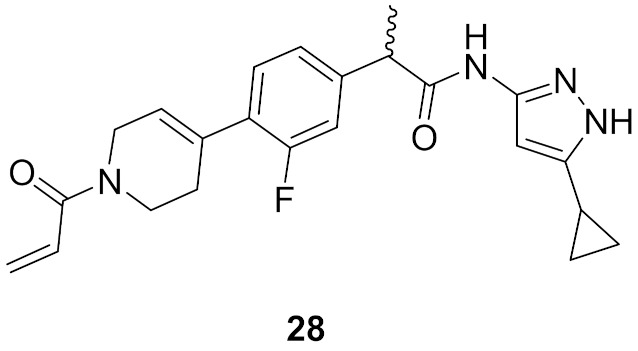	9 (CDK12) 5.8 (CDK13)	-
EGFR	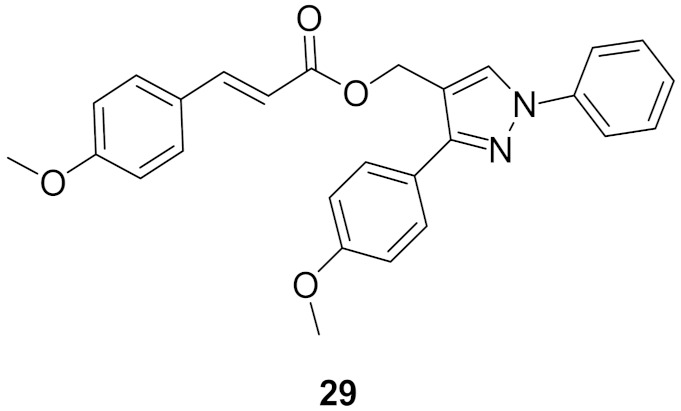	210	Antiproliferative effect against MCF-7 breast cancer cell line (IC_50_ = 0.30 μM).
FGFR	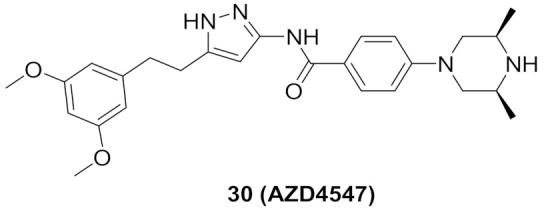	0.2 (FGFR1) 2.5 (FGFR2) 1.8 (FGFR3) 165 (FGFR4)	Orally bioavailable, clinical candidate for lymphoma, glioma, lung, breast, gastric, and esophageal types of cancer.
	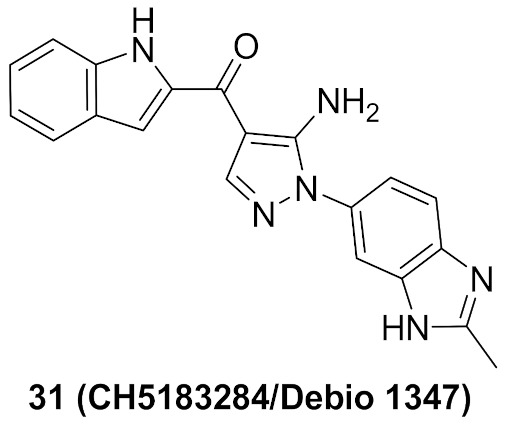	9.3 (FGFR1) 7.6 (FGFR2) 22 (FGFR3) 290 (FGFR4)	Antiproliferative IC_50_ values against gastric SNU-16 and colon HCT116 cancer cell lines are 17 nM and 5.9 µM, respectively.
	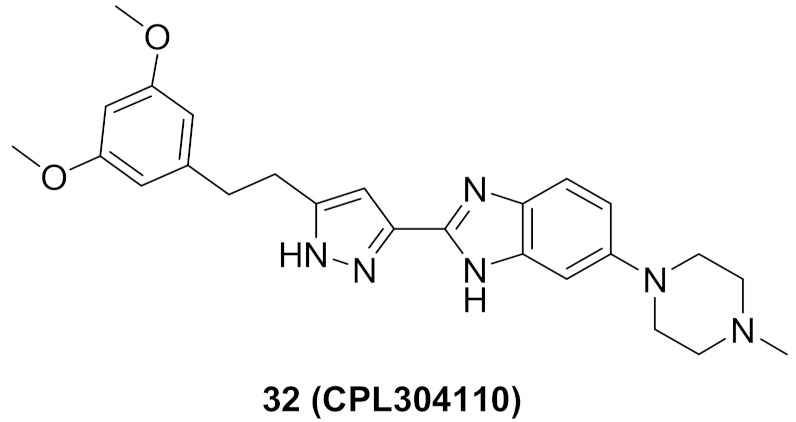	0.75 (FGFR1) 0.5 (FGFR2) 3.05 (FGFR3) 87.9 (FGFR4)	Antiproliferative activity against FGFR-2-amplified SNU-16 gastric cancer cell line (IC_50_ = 85.64 nM).
IKK	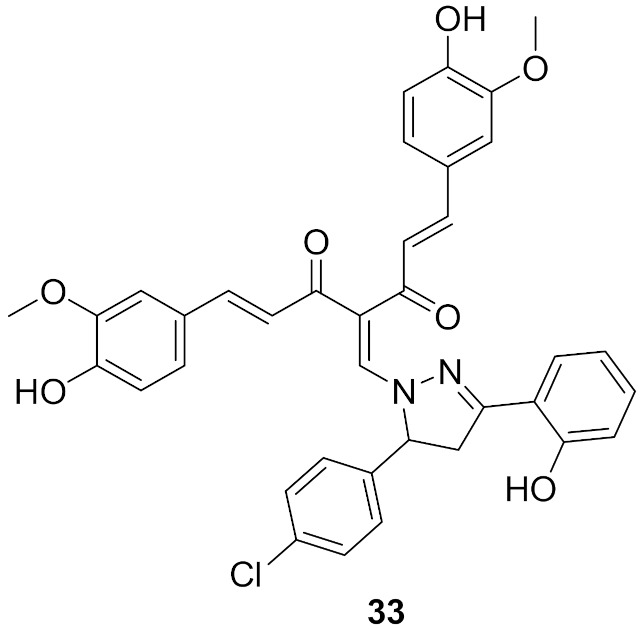	-	Antiproliferative activity against HeLa cervical cancer cell line (IC_50_ = 14.2 μg/mL).
IRAK4	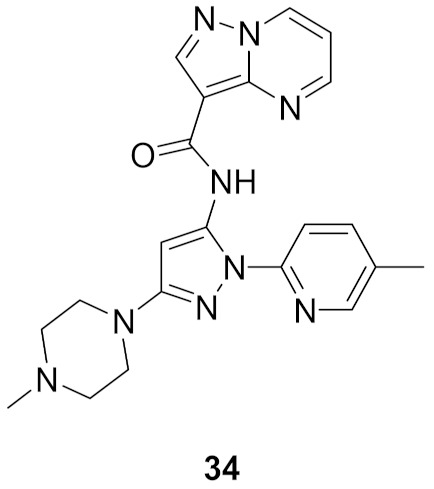	5	Strong potency (IC_50_ = 83 nM) against lipopolysaccharide-induced THP1-XBlue cells.
	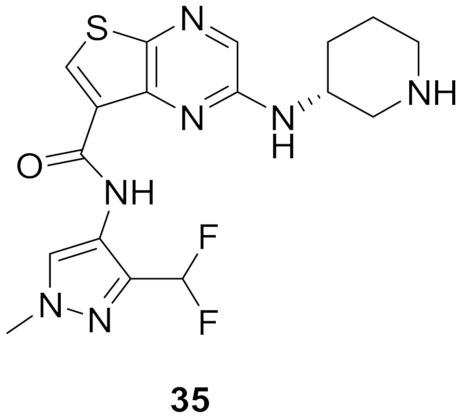	0.4	Good permeability (25 × 10^−6^ cm/s) in MDCK cells.
	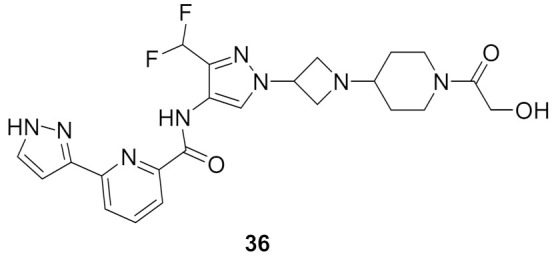	0.51	-
ITK	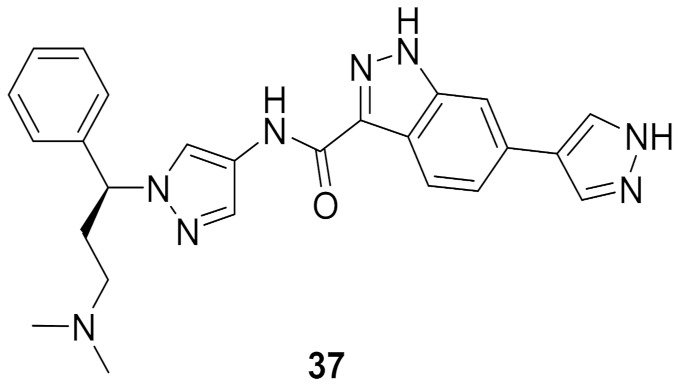	Ki = 0.1 nM	Multi-kinase inhibitor.
	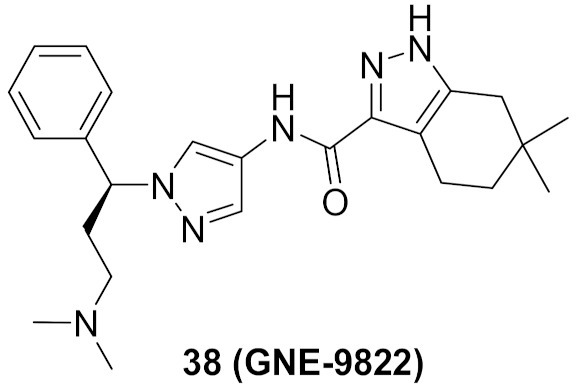	Ki = 0.5 nM	Higher kinase selectivity, permeability, and oral bioavailability than compound 37.
	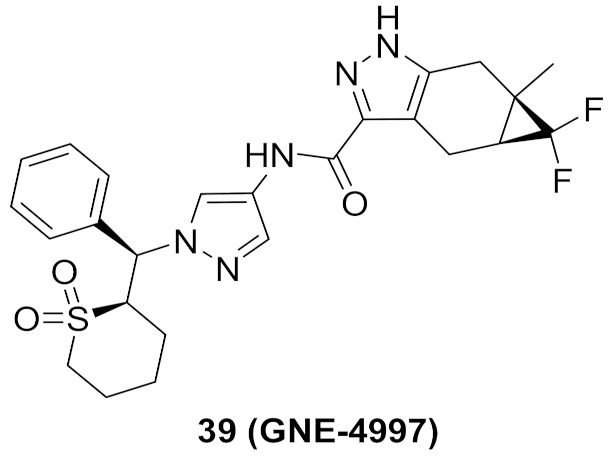	Ki = 0.09 nM	Improved potency, selectivity, and less toxicity.
JAK	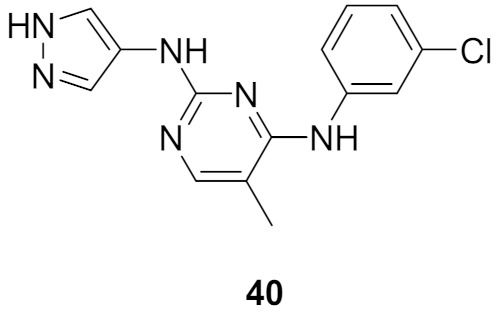	3.4 (JAK1) 2.2 (JAK2) 3.5 (JAK3)	Antiproliferative activity: IC_50_ against PC-3 IC_50_ = 1.08 μM, MCF-7 IC_50_ = 1.33 μM, HEL IC_50_ = 1.08 μM, K562 IC_50_ = 0.77 μM, MOLT4 IC_50_ = 1.61 μM.
	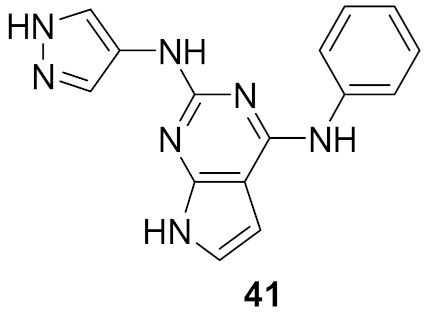	-	Antiproliferative activity against HEL (IC_50_ = 0.35 μM) and K562 (IC_50_ = 0.37 μM).
	* 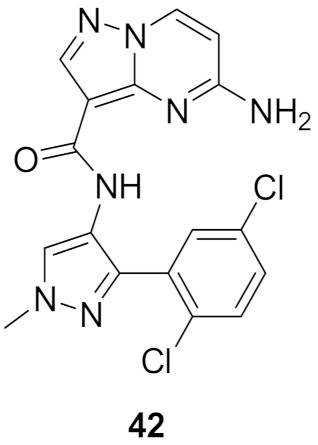 *	Ki = 2.5 nM (JAK2)	Potent inhibitory activity in a JAK2-driven SET2 cell-based assay (IC_50_ = 131 nM). Low potential for reversible inhibition of five major human CYP450 isozymes, and good in vitro permeability profile.
	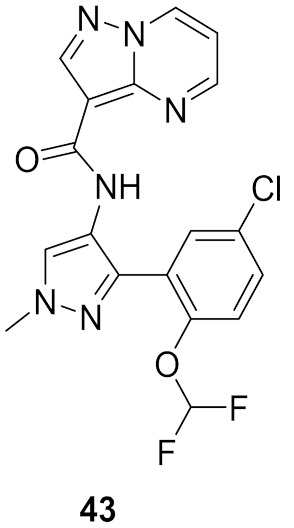	Ki = 0.21 nM (JAK1) Ki = 0.088 nM (JAK2)	IC_50_ of 4.7 nM in IL-13 stimulated BEAS-2B cells.
	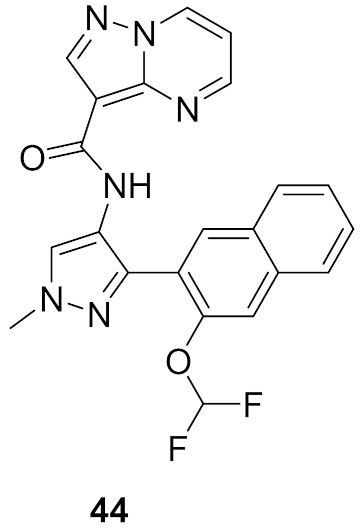	Ki = 0.31 nM (JAK1) Ki = 0.14 nM (JAK2)	IC_50_ of 6.4 nM in the IL-13-pSTAT6 cell-based assay.
	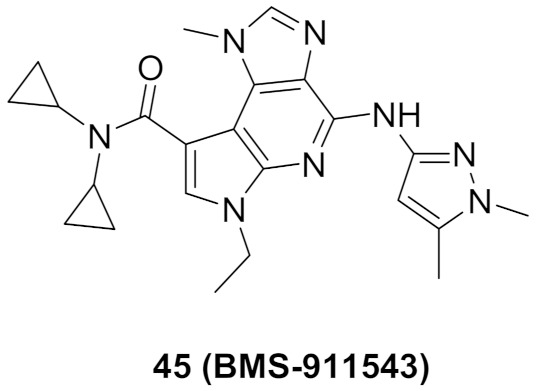	75(JAK1) 1.1 (JAK2) 360 (JAK3)	In vivo reduction of reticulocytes and subsequent reductions in red blood cell mass as well as a decrease in platelets.
JNK	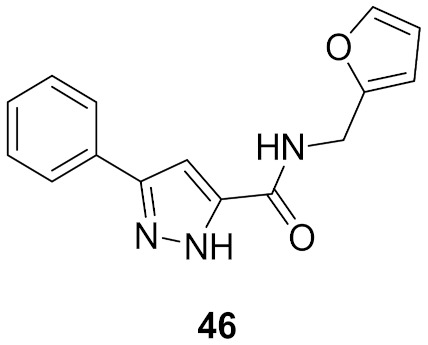	2800 (JNK1)	In vivo anti-inflammatory activity against carrageenan-induced paw edema model in rats.
	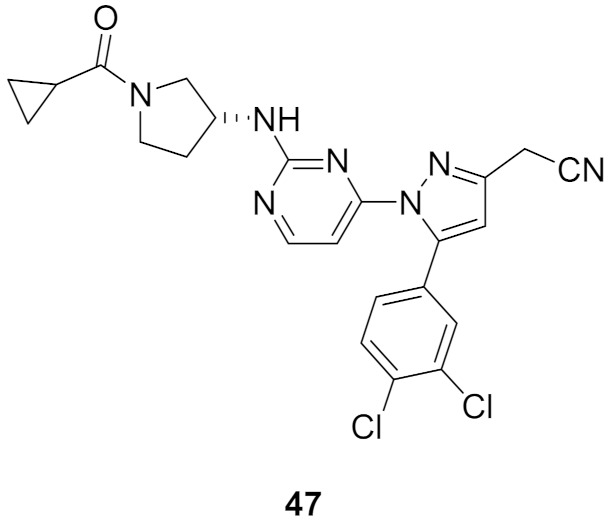	227 (JNK3)	-
LRRK	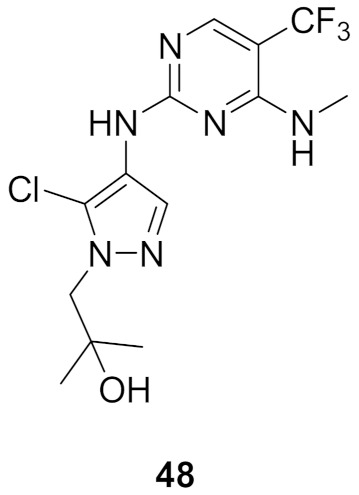	9 (LRRK2)	-
	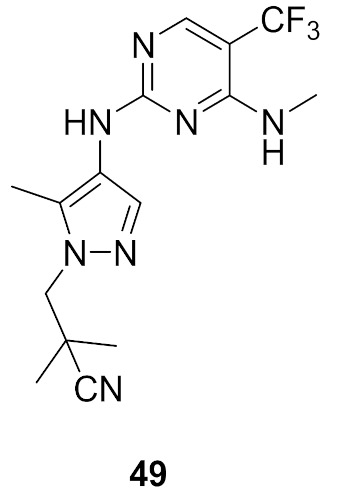 (GNE-0877)	0.7 (LRRK2)	Improved human hepatocyte stability, brain exposure, and lower ability to inhibit or induce CYP compared to compound **48**.
	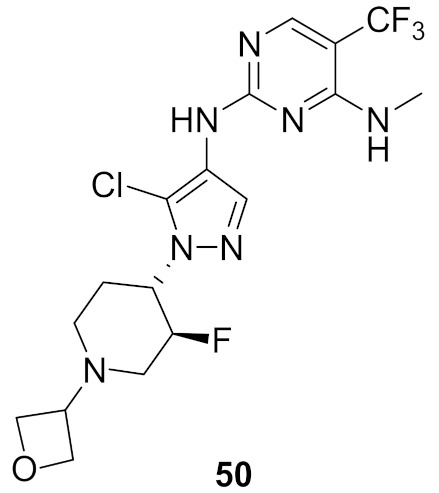 (GNE-9605)	2 (LRRK2)	Improved human hepatocyte stability, brain exposure, and lower ability to inhibit or induce CYP compared to compound **48**.
	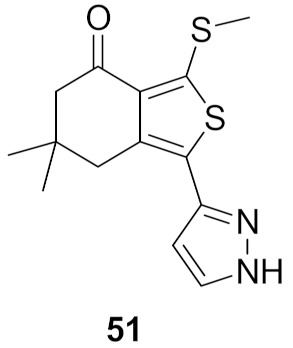	-	-
	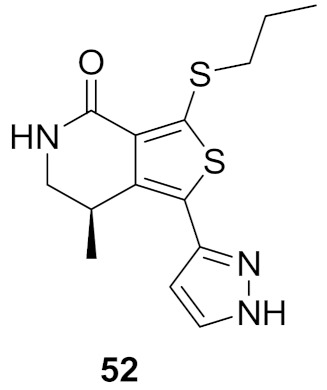	K_i_ = 84 nM (wild-type LRRK2) and 39 nM (G2019S mutant type LRRK2)	98% oral bioavailability.
LsrK	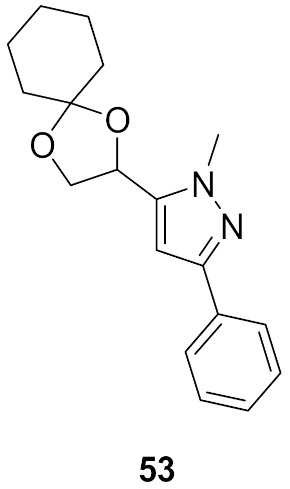	119,000	-
MEK/ERK	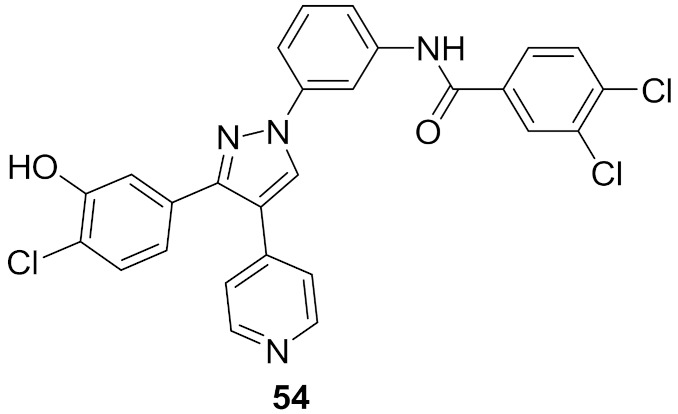	-	Antiproliferative activity against the A375P melanoma cell line (IC_50_ = 6.7 µM).
	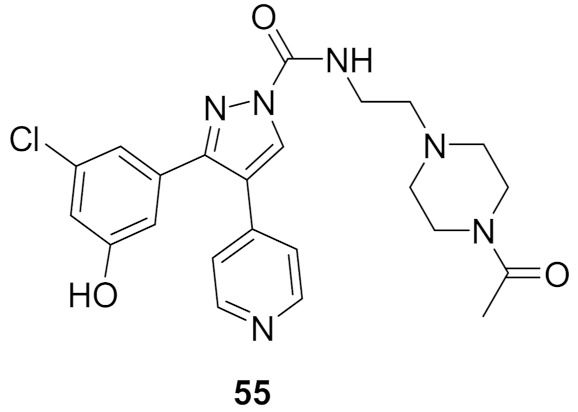	-	Antiproliferative activity against the MDA-MB-435 melanoma cell line (IC_50_ = 2.7 µM). Kinase inhibition was confirmed by Western blotting. IC_50_ = 0.30 µM against COX-2.
	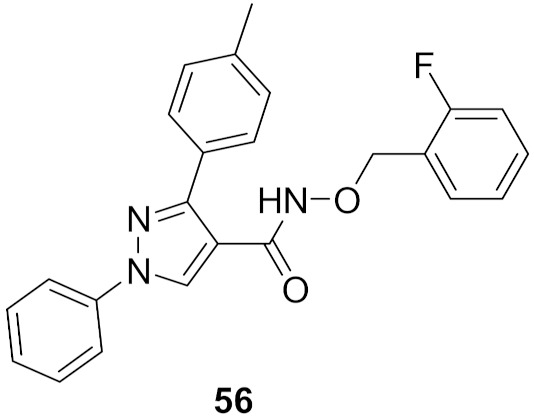	-	IC_50_ = 91 nM against recombinant proteins of the RAF-MEK-ERK cascade. GI_50_ of 1.18, 2.11, and 0.26 μM against HeLa, MCF-7, and A549 cell lines, respectively.
P38α/MAPK14	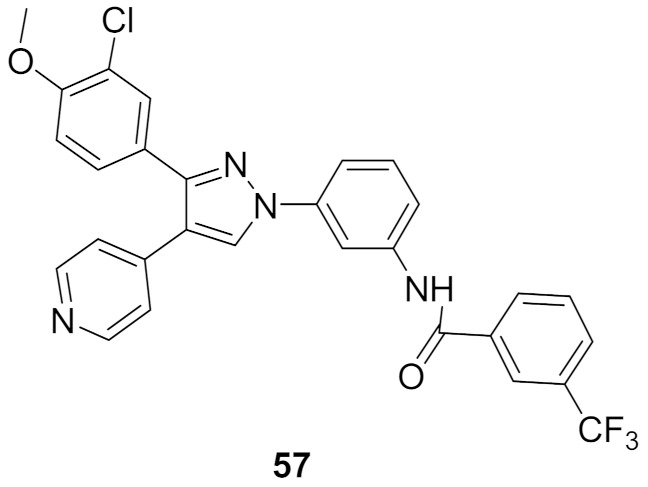	22	Inhibition of TNF-α production in lipopolysaccharide-stimulated THP-1 human cells. In vivo anti-inflammatory activity.
	* 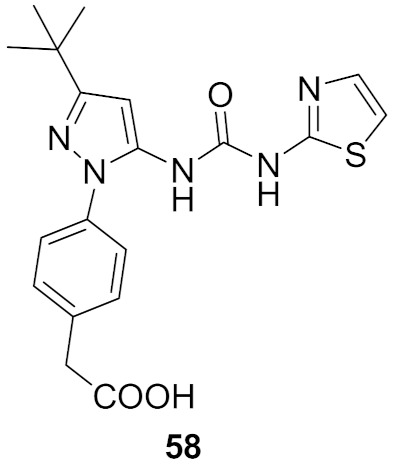 *	135	Poor cellular permeability due to its highly charged carboxylate group. Its ethyl ester analogue could inhibit phosphorylation of MK2 in HeLa cells (IC_50_ value = 6 µM) but its IC_50_ value against p38α is 639 nM.
	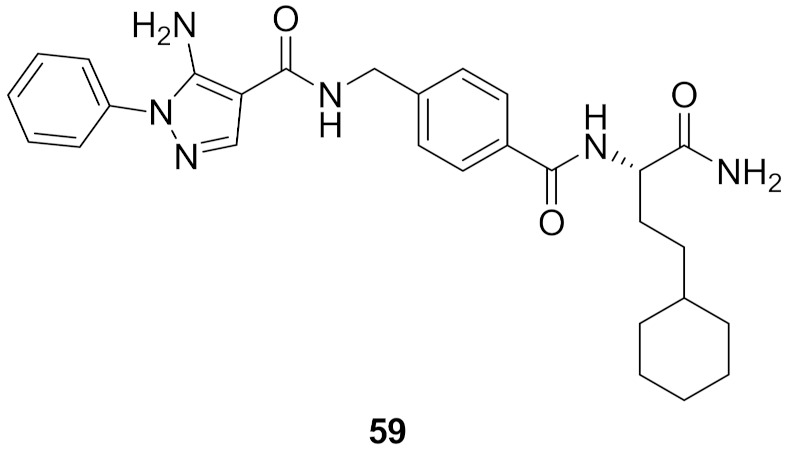 (VPC00628)	7	High selectivity against p38α and p38β.
	* 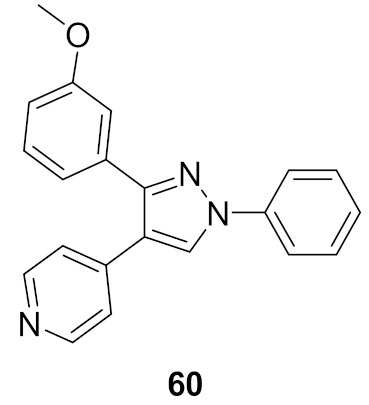 *	515	Antiproliferative activity against RPMI-8226 and K-562 leukemia cell lines in addition to the MDA-MB-468 breast cancer cell line (IC_50_ values are 1.71, 3.42, and 6.70 µM, respectively).
PDK4	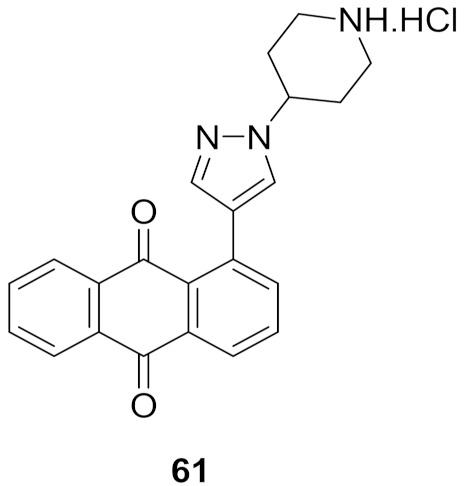	84	Enhanced glucose tolerance in a diet-induced obesity model in mice. Alleviated the allergic reactions in a passive cutaneous anaphylaxis model in mice.
Pim	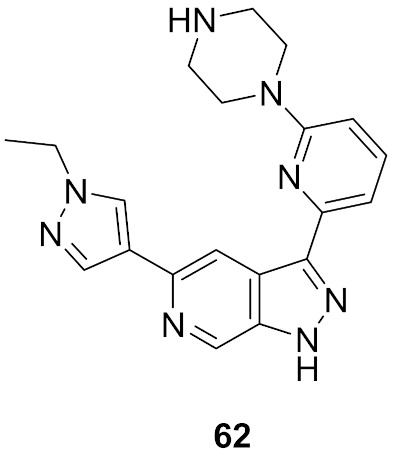	Ki = 0.073 nM (Pim1), 0.473 (Pim2), and 0.041 (Pim3)	Antiproliferative activity against MM1.s myeloma cell line (IC_50_ = 0.64 µM).
	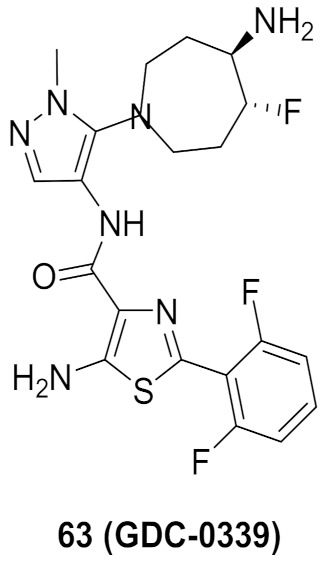	Ki = 0.03 nM (Pim1), 0.1 (Pim2), and 0.02 (Pim3)	Promising in vivo activity against MM1.s and RPMI 8226 mice models of multiple myeloma.
RAF	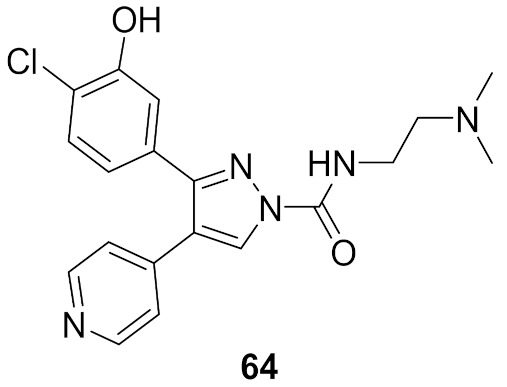	-	Antiproliferative activity against the A375P melanoma cell line (IC_50_ = 4.5 µM).
	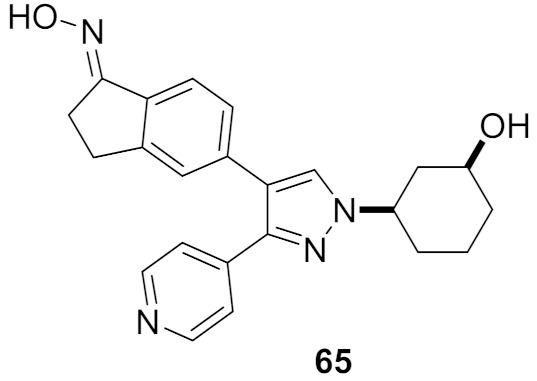	0.04 (wild-type B-RAF)	Its *trans* isomer (with the hydroxyl group behind the plane) is less potent against the kinase (IC_50_ = 0.09 nM).
	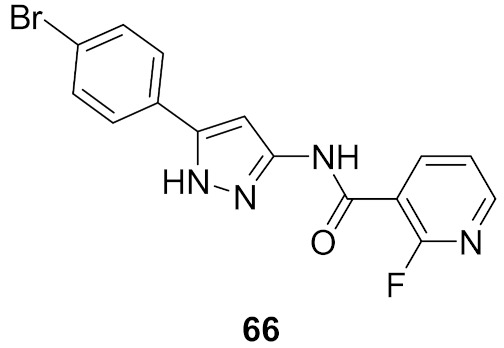	330 (V600E-B-RAF)	Antiproliferative activity against WM266.4 and A375 melanoma cell lines with IC_50_ values of 2.63 and 3.16 µM, respectively
	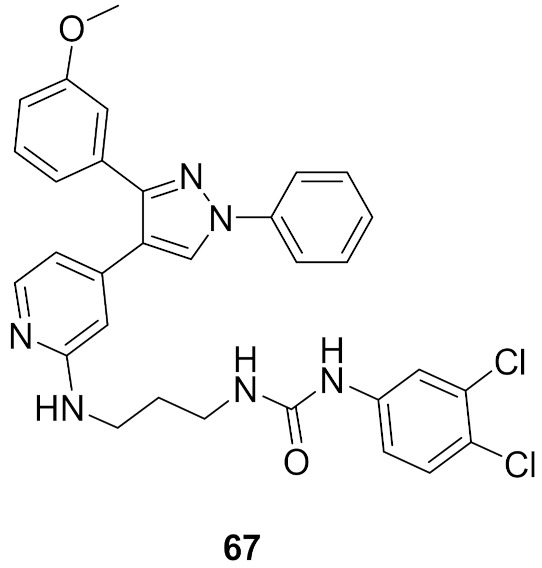	770 (V600E-B-RAF) and 1500 (RAF1)	One-digit micromolar IC_50_ values against different cancer cell lines.
	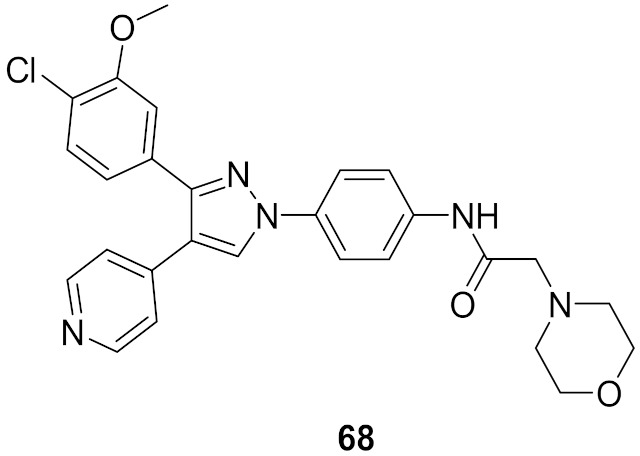	2.98 (V600E-B-RAF)	Antiproliferative activity against the A375 melanoma cells (IC_50_ = 1.82 µM).
	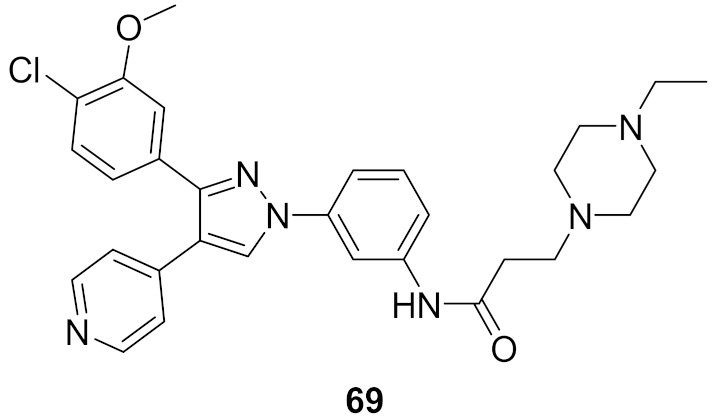	99.17% inhibition at 10 µM (V600E-B-RAF)	IC_50_ values within sub-micromolar range (0.27–0.92 µM) against nine cancer cell lines of nine cancer types. Against the A375 melanoma cell line, its IC_50_ value is 0.82 µM.
	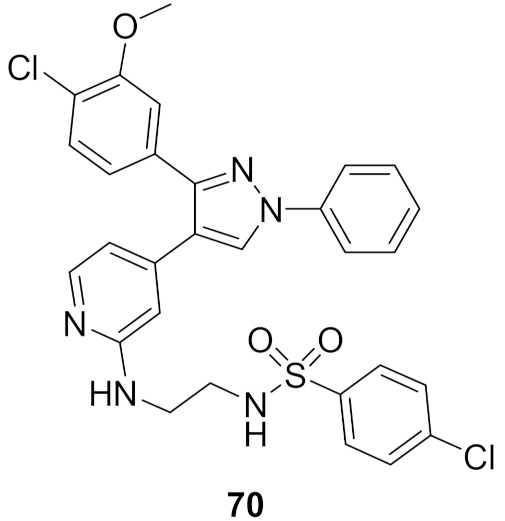	-	78.04%, 74.47%, and 72.46% inhibition at 10 µM concentration against RAF1, V600E-B-RAF, and V600K-B-RAF kinases, respectively.
	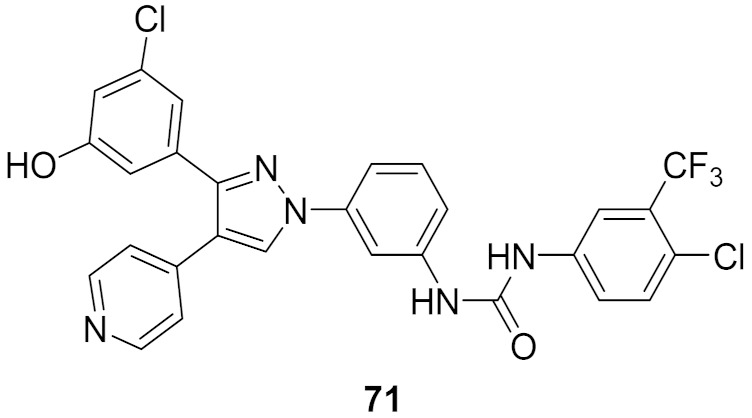	7 (V600E-B-RAF)	Mean IC_50_ value against the NCI nine subpanels was within the range of 1.98–3.26 µM.
	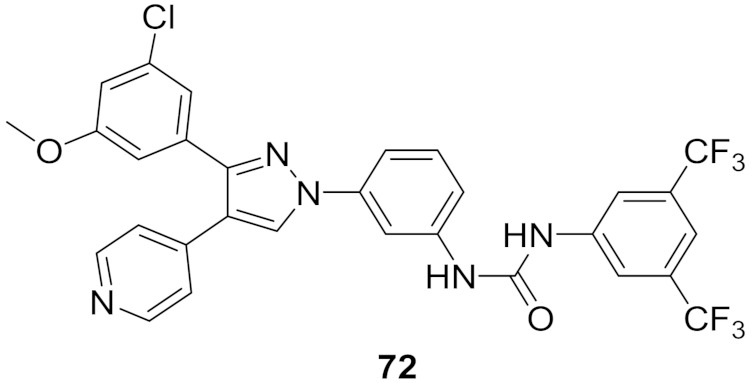	390 (V600E-B-RAF)	IC_50_ values are within the submicromolar range against most of the tested cell lines (NCI-60 panel). Induced apoptosis in the RPMI-8226 leukemia cell line with an EC_50_ of 1.52 µM.
ROS	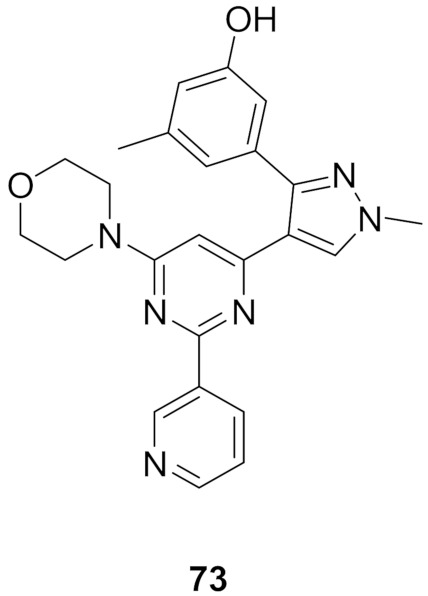	13.6	-
Src	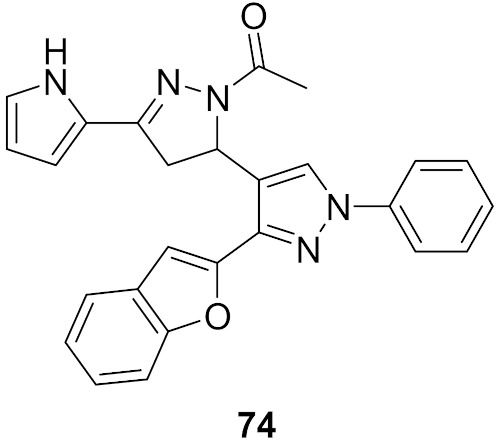	59% inhibition at 10 µM concentration	Antiproliferative activity against CCRF-CEM and MOLT-4 leukemia cell lines (IC_50_ = 1.00 µM against both of them).
TGFβ/ALK	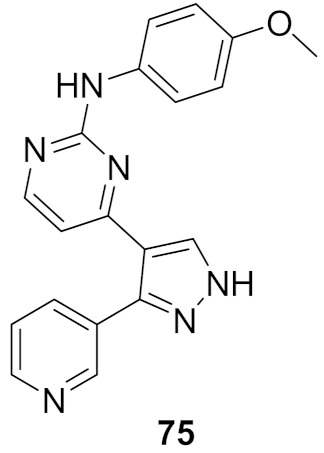	684 (R206H mutated ALK2)	Promising lead compound for treatment of fibrodysplasia ossificans progressive.
	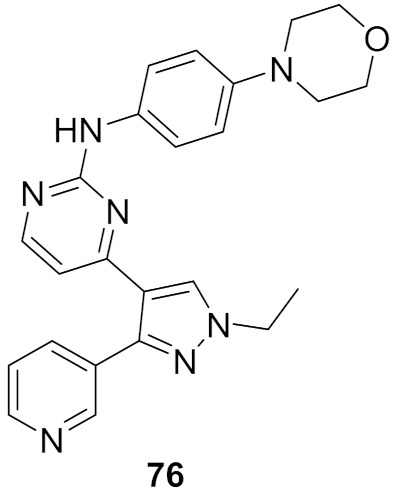	25.6 (R206H mutated ALK2)	Good permeability and in vivo pharmacokinetic properties.
	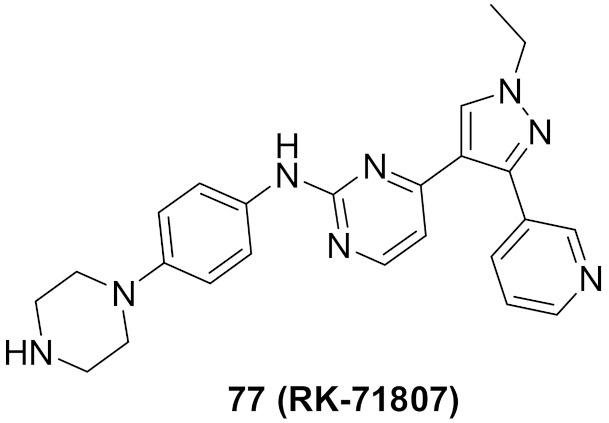	9.4	Promising lead compound for treatment of fibrodysplasia ossificans progressive. Improved aqueous solubility compared to compound 75.
	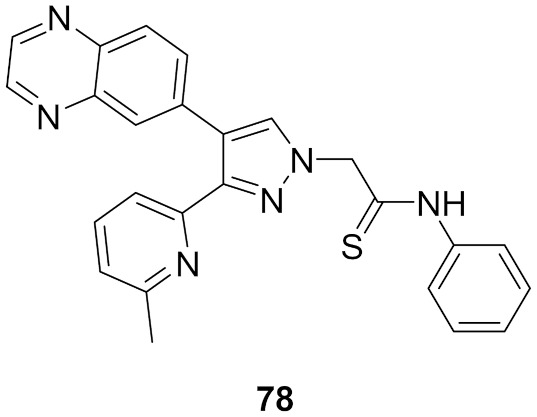	13 (TGFβ type 1/ALK5)	Inhibited luciferase activity by 80% at 0.1 μM. In-cell kinase inhibition.
	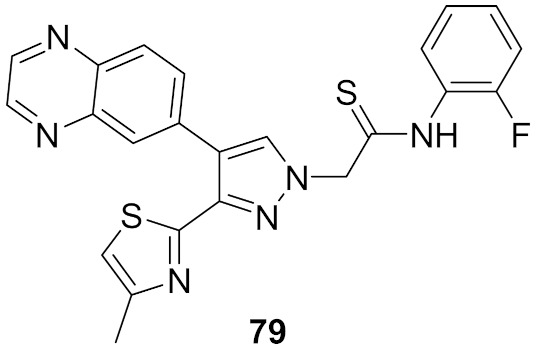	280 (TGFβ type 1/ALK5)	>35-fold more selective against ALK5 compared to p38α MAPK.
	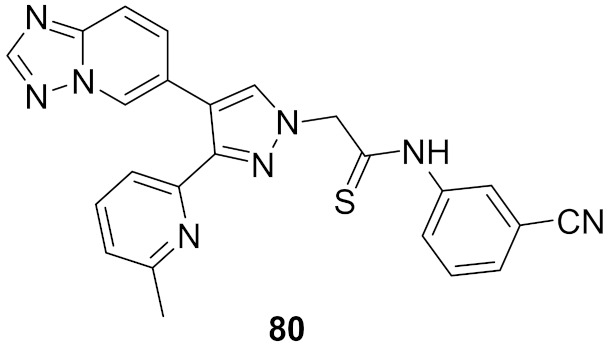	18 (TGFβ type 1/ALK5)	In-cell kinase inhibition.
	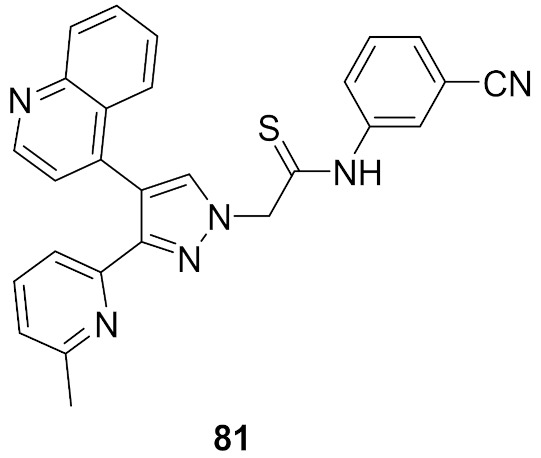	69 (TGFβ type 1/ALK5)	Inhibitory effects against the p38α kinase (IC_50_ = 104 nM).
	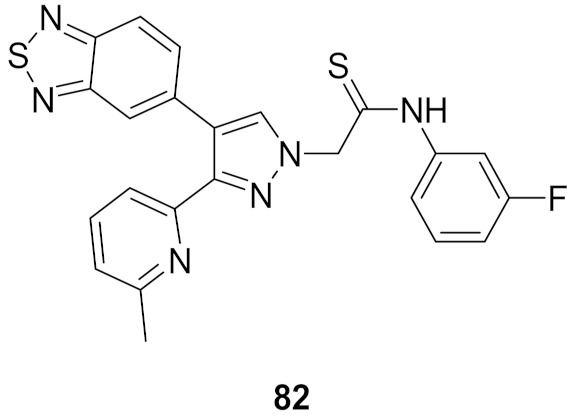	30 (TGFβ type 1/ALK5)	Potential inhibitor of collagen I and α-SMA protein and mRNA expressions in TGFβ-induced LX-2 human hepatic stellate cells.
Trk	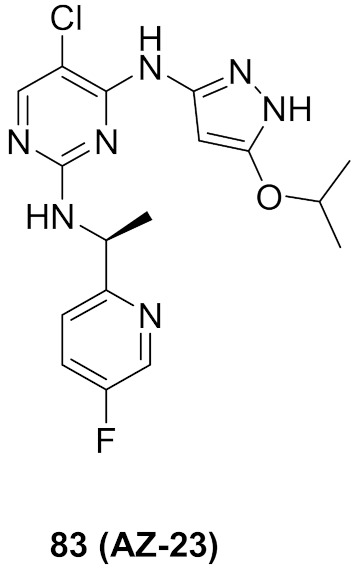	2 (TrkA) 8 (TrkB)	-
	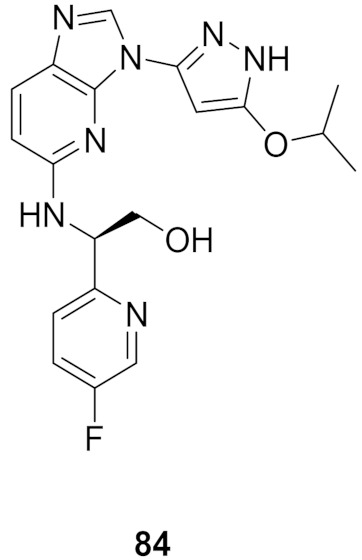	-	Potent inhibitor of TrkA with an IC_50_ value of 0.5 nM in a cellular assay. 29% oral bioavailability. High aqueous solubility and safety against hERG.
	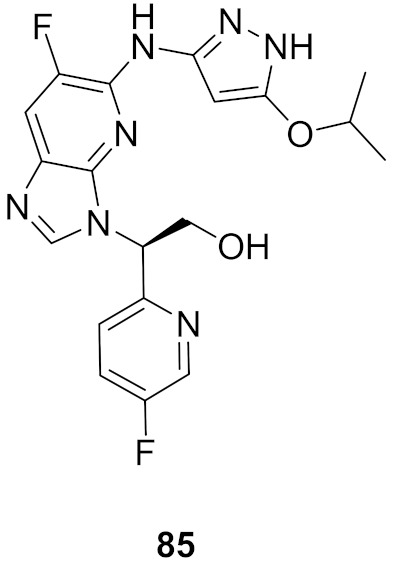	-	Potent inhibitor of TrkA with an IC_50_ value of 0.5 nM in a cellular assay.54% oral bioavailability. High aqueous solubility and safety against hERG.
	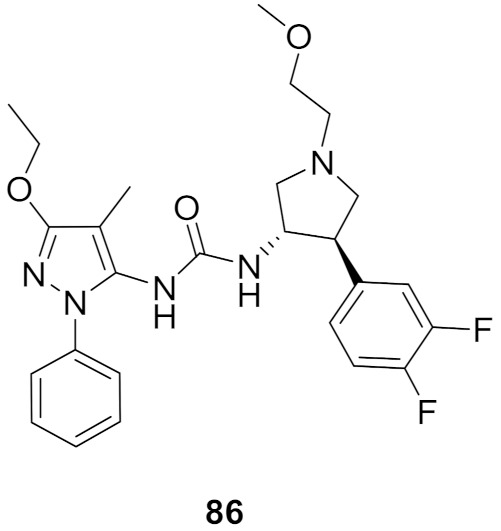	2.7 (TrkA)	Higher selectivity against TrkA than TrkB and TrkC.
	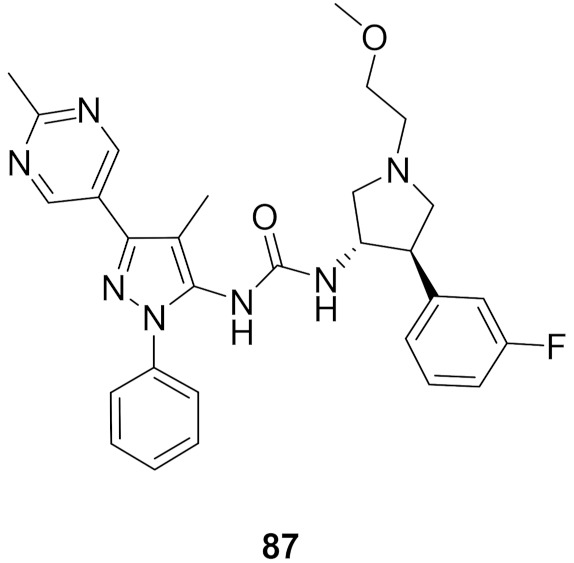	-	Rapid association rate with the TrkA crystal structure, thus binds to the inactive conformation of the kinase (i.e., type II TrkA inhibitor).
	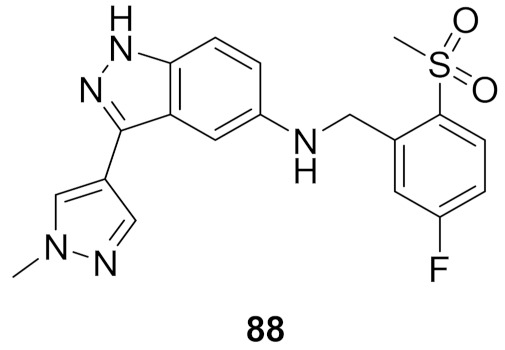	0.2 (TrkA)	In vivo activity in CFA-induced thermal hypersensitivity model.
VEGFR	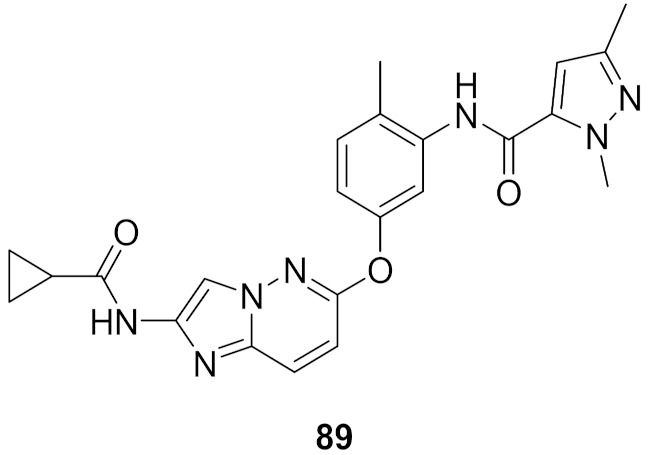	0.95 (VEGFR2)	Decreased the proliferation of VEGF stimulated HUVEC with an IC_50_ of 0.30 nM. In vivo anticancer activity in a mouse xenograft model of human lung adenocarcinoma A549 cells.
	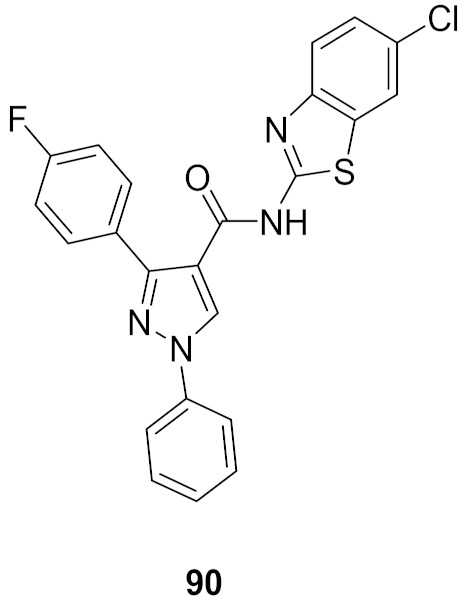	97 (VEGFR2)	Antiproliferative activity against HT-29 colon cell line (IC_50_ = 3.32 μM), PC-3 prostate cells (IC_50_ = 3.17 μM), A549 lung cells (IC_50_ = 3.87 μM), and U87MG glioblastoma cells (IC_50_ = 6.77 μM).
	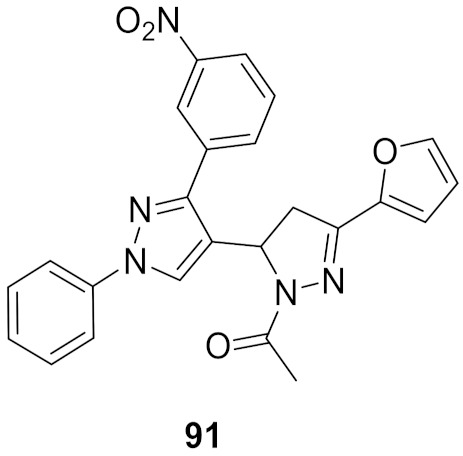	-	In-cell kinase inhibition. Antiproliferative activity against the MCF-7 cell line (IC_50_ = 18.35 µM).
Multikinase inhibitors	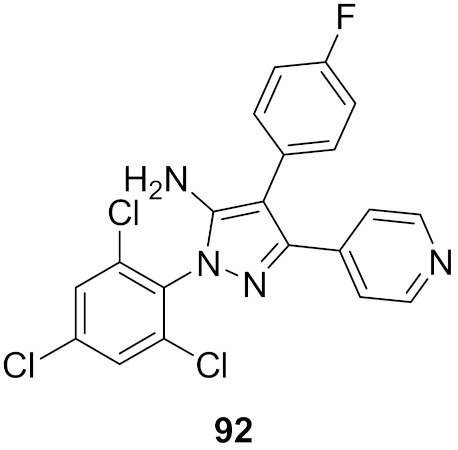	Inhibitor of VEGFR2, Src, B-RAF (wild-type), V600E-B-RAF, EGFR (wild-type), and L858R-EGFR with IC_50_ values of 34, 399, 270, 592, 113, and 31 nM, respectively.	
	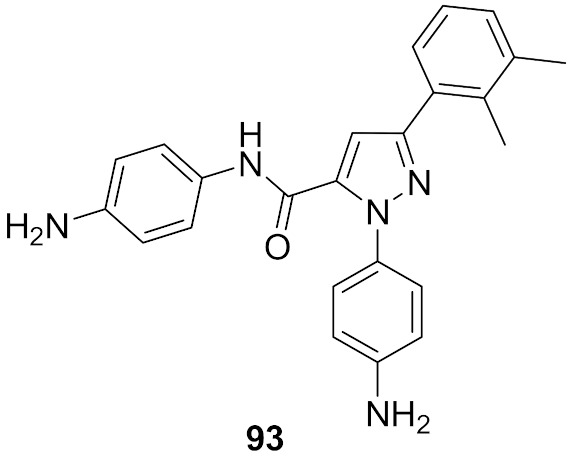	Multi-kinase inhibitory effects against AKT2, GSK-3β, PI3K, EGFR, IGFR, CDK2, Aurora A, and MAPK.	Antiproliferative activity against SNU449 hepatocellular carcinoma cell line.
	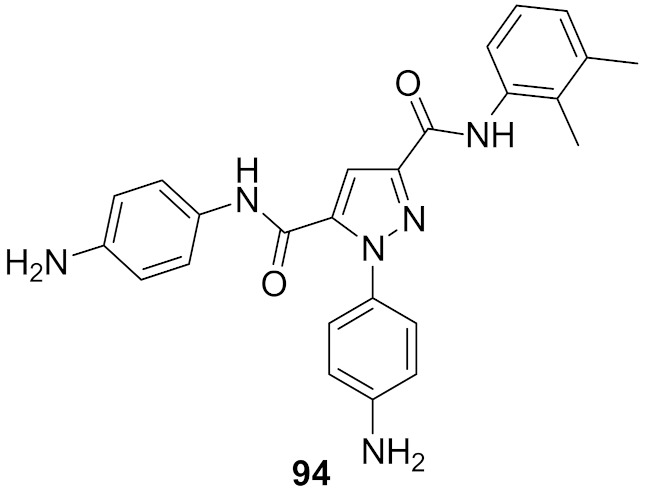	Multikinase inhibitory effects against AKT2, GSK-3β, PI3K, EGFR, IGFR, CDK2, Aurora A, and MAPK.	Antiproliferative activity against SNU449 hepatocellular carcinoma cell line.
	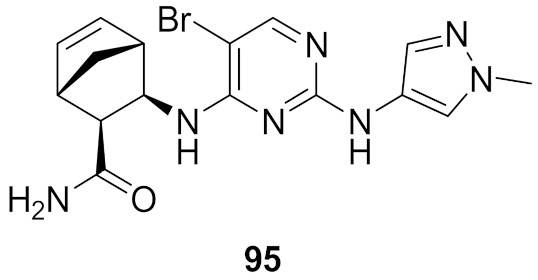	Dual KDR/Aurora B activity	Narrow therapeutic index.
	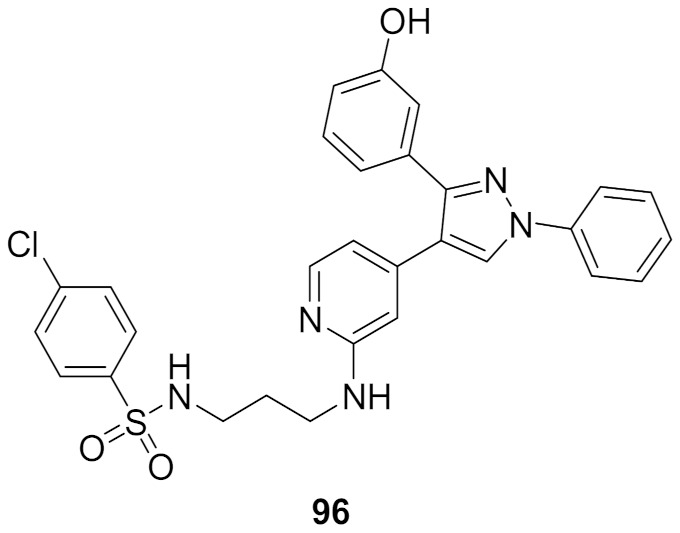	Inhibitory effect against B-RAF (wild-type), V600E-B-RAF, p38α, JNK1, and JNK2 kinases (inhibition % values at 10 µM concentration are 72.56%, 93.67%, 86.54%, 99.05%, and 98.49%, respectively).	Antiproliferative activity against the A498 renal carcinoma cell line (IC_50_ = 0.33 µM). JNK1 and JNK2 are the most sensitive among them (IC_50_ = 350 and 360 nM, respectively).
	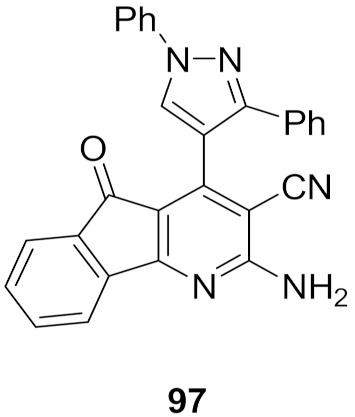	>94% inhibition of AKT1, AKT2, V600E-B-RAF, EGFR, p38α, and PDGFRβ at 100 µM.	Antiproliferative activity against MCF7 breast cancer cell line (IC_50_ = 6.53 µM).
	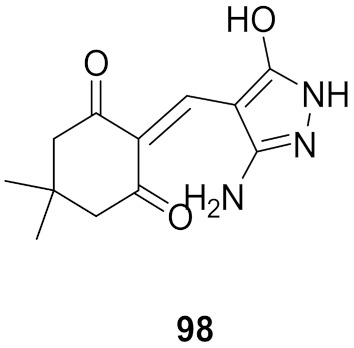	c-Kit, FLT-3, VEGFR-2, EGFR, PDGFR, and Pim-1 kinases (IC_50_ 260–610 nM).	Antiproliferative activity against A549 (lung), H460 (lung), HT29 (colon), MKN-45 (gastric), U87MG (glioma), and SMMC-77217721 (hepatic) cancer cell lines (IC_50_ values from 0.29–0.42 µM).
	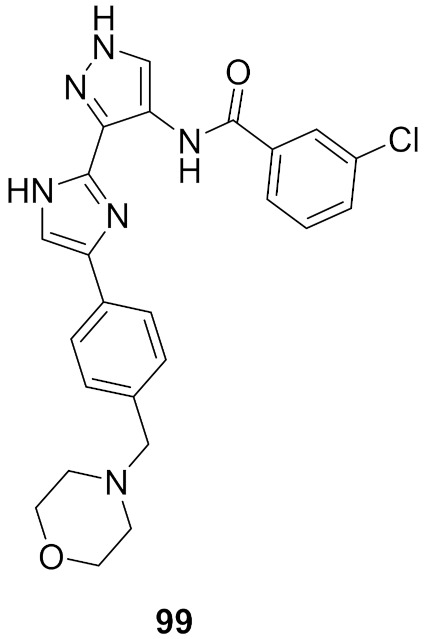	JAK2, JAK3, Aurora A, and Aurora B (IC_50_ = 166, 57, 939, 583 nM, respectively).	Antiproliferative activity against K562 leukemia cell line and HCT116 colon cancer cell line (IC_50_ = 6.726 and 15.054 µM, respectively).
	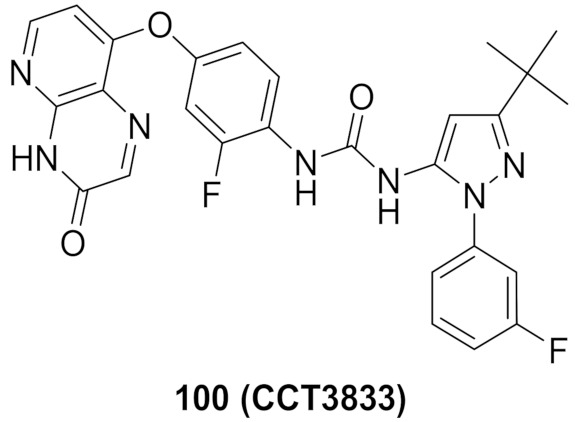	Inhibited B-RAF, C-RAF, and Src kinases both in vitro and in vivo.	In vivo activity and phase I clinical trials in volunteers with solid tumors. Increased the progression-free survival in a patient suffering from KRAS (G12V) spindle cell sarcoma.

## Data Availability

Not applicable.

## Abbreviations

ALK5, Activin receptor-Like Kinase 5; ALS, amyotrophic lateral sclerosis; ASK1, Apoptosis signal-regulating kinase 1; ATP, adenosine triphosphate; CDK, cyclin-dependent kinase; CFA, complete Freund’s adjuvant; Chk2, checkpoint kinase 2; CML, chronic myeloid leukemia; COX-1, Cyclooxygenase-1; COX-2, Cyclooxygenase-2; EGFR, Epidermal Growth Factor Receptor; ELISA, enzyme-linked immunosorbent assay; ERK, Extracellular signal-regulated kinase; FDA, Food and Drug Administration; FGFR, fibroblast growth factor receptor; GI_50_, concentration at which 50% growth inhibition is observed; HDAC, histone deacetylase; HEL, human erythroleukemia; HER-2, human epidermal growth factor receptor 2; hPBMCs, human peripheral blood mononuclear cells; HUVEC, human umbilical vein endothelial cells; IKKβ; inhibitor of nuclear factor kappa-B kinase subunit β; IL, interleukin; IRAK4, interleukin-1 receptor associated kinase 4; ITK, Interleukin-2 inducible T-cell kinase; JAK, Janus kinase; JNK, c-Jun *N*-terminal kinases; KD, kinase domain; LRRK2, leucine-rich repeat kinase 2; MAPKs, mitogen-activated protein kinases; MDCK, Madin–Darby canine kidney; MDR, multidrug resistance; MM, molecular mechanics; MS, multiple sclerosis; NCI, National Cancer Institute; PDK4, pyruvate dehydrogenase kinase 4; QM, quantum mechanics; QS, quorum sensing; QSAR, quantitative structure-activity relationship; SAR, structure-activity relationship; TDI, time-dependent inhibition; TNF-α, tumor-necrosis factor-α; VEGFR, vascular endothelial growth factor receptors.
